# Trends and Disparities in Diet Quality Among US Adults by Supplemental Nutrition Assistance Program Participation Status

**DOI:** 10.1001/jamanetworkopen.2018.0237

**Published:** 2018-06-15

**Authors:** Fang Fang Zhang, Junxiu Liu, Colin D. Rehm, Parke Wilde, Jerold R. Mande, Dariush Mozaffarian

**Affiliations:** 1 Friedman School of Nutrition Science and Policy, Tufts University, Boston, Massachusetts; 2 Department of Epidemiology and Population Health, Albert Einstein College of Medicine, Bronx, New York

## Abstract

**Importance:**

Unhealthful diet is a top contributor to chronic diseases in the United States. There are growing concerns about disparities in diet among US adults, especially for those who participate in the Supplemental Nutrition Assistance Program (SNAP), the largest federal food assistance program. It remains unclear how these disparities may have changed over time.

**Objective:**

To assess whether disparities in key food groups and nutrients according to participation and eligibility for SNAP have persisted, improved, or worsened over time among US adults.

**Design, Setting, and Participants:**

This survey study examined a nationally representative sample of 38 696 adults aged 20 years or older: 6162 SNAP participants, 6692 income-eligible nonparticipants, and 25 842 higher-income individuals from 8 cycles of the National Health and Nutrition Examination Survey (1999-2014). Data analysis was conducted between January 1, 2017, and December 31, 2017.

**Exposures:**

Survey-weighted, energy-adjusted diet by SNAP participation status.

**Main Outcomes and Measures:**

Mean diet scores and proportions of US adults meeting poor, intermediate, or ideal diet scores based on the American Heart Association (AHA) 2020 Strategic Impact Goals for diet, including 8 components (fruits and vegetables; whole grains; fish and shellfish; sugar-sweetened beverages; sodium; nuts, seeds, and legumes; processed meats; and saturated fat).

**Results:**

The survey included 38 696 respondents (20 062 female [51.9%]; 18 386 non-Hispanic white [69.8%]; mean [SD] age, 46.8 [14.8] years). Participants of SNAP were younger (mean [SD] age, 41.4 [15.6] years) than income-eligible nonparticipants (mean [SD] age, 44.9 [19.6] years) or higher-income individuals (mean [SD] age, 47.8 [13.6] years); more likely to be female (3552 of 6162 [58.6%] vs 3504 of 6692 [54.8%] and 13 006 of 25 842 [50.4%], respectively); and less likely to be non-Hispanic white (2062 of 6162 [48.2%] vs 2594 of 6692 [56.0%] and 13 712 of 25 842 [75.8%], respectively). From surveys conducted in 2003 and 2004 to those conducted in 2013 and 2014, SNAP participants had less improvement in AHA diet scores than both income-eligible nonparticipants and higher-income individuals (change in mean score = 0.57 [95% CI, −2.18 to 0.33] vs 2.56 [95% CI, 0.36-4.76] and 3.84 [95% CI, 2.39-5.29], respectively; *P* = .04 for interaction). Disparities persisted for most foods and nutrients and worsened for processed meats, added sugars, and nuts and seeds. In 2013 to 2014, a higher proportion of SNAP participants had poor diet scores compared with income-eligible nonparticipants and higher-income individuals (461 of 950 [53.5%] vs 247 of 690 [38.0%] and 773 of 2797 [28.7%]; *P* < .001 for difference), and a lower proportion had intermediate diet scores (477 of 950 [45.3%] vs 428 of 690 [59.8%] and 1933 of 2797 [68.7%]; *P* < .001 for difference). The proportion of participants with ideal diet scores was low in all 3 groups (12 of 950 [1.3%] vs 15 of 690 [2.2%] and 91 of 2797 [2.6%]; *P* = .26 for difference).

**Conclusions and Relevance:**

Dietary disparities persisted or worsened for most dietary components among US adults. Despite improvement in some dietary components, SNAP participants still do not meet the AHA goals for a healthful diet.

## Key Points

**Question:**

Have disparities in diet quality of US adults according to participation and eligibility for food assistance persisted, improved, or worsened over the past 15 years?

**Findings:**

This survey study found that despite an overall improvement in diet quality among US adults between 1999 and 2014, disparities persisted or worsened for most dietary components when comparing participants of the Supplemental Nutrition Assistance Program (SNAP) with income-eligible nonparticipants and higher-income individuals. For nearly all dietary components, SNAP participants do not meet recommendations for a healthful diet.

**Meaning:**

Evidence-based nutrition policies are needed to reduce diet-related health disparities in the United States.

## Teaser

This national survey study uses NHANES data to characterize trends in diet quality among US adults between 1999 and 2014 and differences in those trends among Supplemental Nutrition Assistance Program (SNAP) participants, income-eligible nonparticipants, and higher-income individuals.

## Introduction

Unhealthful diet is one of the top contributors to poor health in the United States,^1^ and disparities in diet quality by socioeconomic status can contribute to the nation’s health disparities. While overall diet quality has modestly improved in the past decade,^2^ there are growing concerns that socioeconomic disparities persist in diet quality of US adults. Low-income individuals have not experienced the same improvements in diet quality as high-income individuals,^2,3^ and for consumption of several foods, this disparity may have widened over time.^3^

Among different dietary programs for low-income households, the Supplemental Nutrition Assistance Program (SNAP) is by far the largest and most important safety net program, providing monthly benefits to approximately 1 in 7 US individuals and representing more than half of the annual budget of the US Department of Agriculture (USDA).^4,5^ Participants in SNAP experience significantly higher all-cause, cardiovascular, and diabetes mortality compared with other American adults.^6,7,8^ Prior studies have shown that disparities exist in diet quality between SNAP participants and higher-income individuals.^9^ However, potential trends in these dietary disparities over time remain unclear.

We assessed whether disparities for overall diet quality and for key foods and nutrients persisted, improved, or worsened over time among adult SNAP participants from 1999 to 2014 compared with both SNAP-eligible nonparticipants and higher-income American adults.

## Methods

### Study Design and Population

We used data from US adults aged 20 years or older completing at least 1 valid 24-hour diet recall, as determined by the National Center for Health Statistics (NCHS), during 8 cycles of the National Health and Nutrition Examination Survey (NHANES) from 1999 through 2014. The response rates of the NHANES ranged from 71% to 85% over the study period.^10^ We used NHANES sampling weights in all analyses, which account for the complex survey design (eg, oversampling of minorities), survey nonresponse, poststratification, and whether 1 or 2 days of diet recalls were completed.^11^ During the study period, 89.0% (38 979 of 43 793) of the NHANES respondents aged 20 years or older provided a single valid diet recall, among whom 69.9% (27 258 of 38 979) also provided a second valid recall. The dietary sampling weights additionally account for the dietary interview–specific nonresponse and day of the week for dietary intake interviews.^11^ All analyses incorporated these survey weights and provided nationally representative estimates of dietary intake. Data analyses were conducted between January 1, 2017, and December 31, 2017. The NCHS Research Ethics Review Board approved NHANES, and all participants provided written informed consent. The Tufts University institutional review board exempted the study from review and waived requirements for patient informed consent.

### SNAP Participation

Individuals were classified as SNAP participants based on reported household participation in SNAP at any point during the past 12 months. Individuals were classified as income-eligible nonparticipants if they did not participate but had a monthly family gross income of less than or equal to 130% of the poverty guideline (ie, family income to poverty ratio ≤1.30, the major eligibility criterion for SNAP), and as higher-income individuals if they had a family income to poverty ratio greater than 1.30.^12^

### American Heart Association Diet Score

As a summary indicator of a healthful diet, a diet score was constructed based on the American Heart Association (AHA) 2020 Strategic Impact Goals for diet,^1^ which have been validated as independently associated with cardiovascular and metabolic outcomes in multiple populations.^13^ The 8 dietary components were fruits and vegetables; fish and shellfish; whole grains; sugar-sweetened beverages (SSBs); sodium; nuts, seeds, and legumes; processed meat; and saturated fat. As previously detailed,^2^ a continuous score (ie, AHA total score) was constructed by summing all 8 components. Based on the AHA 2020 goals for diet, the proportions of US adults with a poor diet (score <32.0, corresponding to <40.0% adherence to the AHA 2020 goals), an intermediate diet (score = 32.0-63.9, corresponding to 40.0%-79.9% adherence), or an ideal diet (score ≥64.0, corresponding to ≥80.0% adherence) were estimated.

### Food Groups and Nutrients

In addition to the 8 AHA dietary components, we evaluated individual food groups and nutrients linked to major health outcomes as well as those of current interest to policy makers or the general public. The USDA Food Patterns Equivalents Database and MyPyramid Equivalents Database, which disaggregate mixed foods into their component parts, were harmonized and used to assess trends in consumption of major food groups.^2^ Food groups (eg, vegetables) were further disaggregated into subgroups (eg, dark green vegetables vs white potatoes) to evaluate trends by subtype. Nutrients were estimated based on cycle-specific versions of the USDA Food and Nutrient Database for Dietary Studies. Intake of all food groups and nutrients was energy adjusted using the residual method to evaluate trends independent of changes in total energy intake, which could relate to nondietary changes such as physical activity, and to minimize measurement error in dietary estimates.

### Statistical Analysis

We estimated the nationally representative population mean intake for key food groups and nutrients for each NHANES cycle among SNAP participants, income-eligible nonparticipants, and higher-income individuals, incorporating the weights from the complex NHANES sample design to account for different sampling probabilities and participation rates. To assess overall diet quality, we calculated AHA diet scores and estimated the proportion of participants meeting criteria for poor, intermediate, and ideal diets. Because such binary estimates are not based on stratum means but on distributions of dietary intake above or below a certain threshold, which are not comparable between single and multiple diet recalls, we restricted all analyses on proportions and corresponding AHA diet scores to participants with 2 nonconsecutive diet recalls (2003 onward).

We assessed the trends in dietary intake and AHA diet scores by treating the 2-year survey cycle as a continuous variable in survey-weighted linear regression models. We also evaluated changes in dietary intake by computing the difference in mean intake between the earliest (1999-2000) and latest (2013-2014) cycles. To assess whether dietary disparities persisted, improved, or worsened over time, we used a survey-weighted Wald test for an interaction term between 2-year survey cycle and SNAP participation status and for an interaction term between change in mean intake and SNAP participation status. To determine the degree to which the observed dietary disparity trends were driven by demographic shifts, we performed sensitivity analyses adjusting for trends in demographic variables (age, sex, race/ethnicity, education, and income).

All statistical analyses were 2-sided and significance was considered at an α level of .05. Stata statistical software version 14 (StataCorp) was used for all analysis. We followed the American Association for Public Opinion Research (AAPOR) reporting guideline for this study.

## Results

The survey included 38 696 respondents (20 062 female [51.9%]; 18 386 non-Hispanic white [69.8%]; mean [SD] age, 46.8 [14.8] years). Survey-weighted characteristics of SNAP participants, income-eligible nonparticipants, and higher-income individuals are presented in Table 1, representing the characteristics of approximately 25.5 million adult SNAP participants, 26.9 million income-eligible nonparticipants, and 158.7 million higher-income US adults. Participants of SNAP were younger (mean [SD] age, 41.4 [15.6] years) than income-eligible nonparticipants (mean [SD] age, 44.9 [19.6] years) or higher-income individuals (mean [SD] age, 47.8 [13.6] years); more likely to be female (3552 of 6162 [58.6%] vs 3504 of 6692 [54.8%] and 13 006 of 25 842 [50.4%], respectively); and less likely to be non-Hispanic white (2062 of 6162 [48.2%] vs 2594 of 6692 [56.0%] and 13 712 of 25 842 [75.8%], respectively). Trends in SNAP participation and accompanying demographic shifts are shown in eTable 1 in the Supplement. From 2003 to 2014, the mean AHA diet score (maximum of 80) among SNAP participants did not significantly change (from 31.5 [95% CI, 29.2-33.8] to 32.1 [95% CI, 30.6-33.6]; *P* = .11 for trend), whereas the mean AHA diet score significantly improved in both income-eligible nonparticipants (from 34.2 [95% CI, 32.6-35.9] to 36.8 [95% CI, 35.3-38.3]; *P* = .004 for trend) and higher-income individuals (from 35.8 [95% CI, 34.6-36.9] to 39.6 [95% CI, 38.7-40.5]; *P* < .001 for trend) (Table 2). The mean change in AHA diet score was 0.57 (95% CI, −2.18 to 0.33) among SNAP participants, 2.56 (95% CI, 0.36-4.76) among income-eligible nonparticipants, and 3.84 (95% CI, 2.39-5.29) among higher-income individuals (*P* = .04 for interaction).

**Table 1.** Sociodemographic Characteristics of US Adults by SNAP Participation Status, 1999-2014

Sociodemographic Characteristics	Weighted Survey Results, % (95% CI)		
	SNAP Participants (n = 6162)	Income-Eligible Nonparticipants (n = 6692)	Higher-Income Individuals (n = 25 842)
Weighted sample size^a^	25 492 952	26 886 509	158 720 405
Age, mean (95% CI), y	41.4 (40.7-42.0)	44.9 (43.5-46.2)	47.8 (47.4-48.2)
Age group, y			
20-34	41.0 (38.7-26.3)	36.7 (33.2-40.4)	25.3 (24.2-26.3)
35-49	28.9 (27.0-30.8)	25.5 (23.6-27.4)	30.0 (28.9-31.1)
50-64	20.9 (19.4-22.5)	17.8 (16.0-19.9)	26.6 (25.7-27.6)
≥65	9.3 (8.4-10.3)	20.0 (18.3-21.8)	18.1 (17.4-18.9)
Sex			
Male	41.4 (40.0-42.9)	45.2 (43.8-46.6)	49.6 (48.9-50.2)
Female	58.6 (57.1-60.0)	54.8 (53.4-56.2)	50.4 (49.8-51.1)
Race/ethnicity			
Non-Hispanic white	48.2 (43.3-53.0)	56.0 (51.7-60.2)	75.8 (73.8-77.6)
Non-Hispanic black	25.6 (22.5-28.9)	12.5 (10.6-14.8)	8.7 (7.7-9.8)
Mexican American	11.8 (9.4-14.7)	15.8 (13.4-18.5)	5.9 (5.1-6.9)
Other Hispanic	9.3 (7.0-12.2)	8.8 (6.7-11.4)	3.8 (3.1-4.5)
Other or mixed race	5.2 (4.1-6.4)	7.0 (5.9-8.3)	5.8 (5.2-6.5)
Education level			
<High school	44.9 (38.3-51.7)	42.6 (37.8-47.4)	12.2 (10.6-14.0)
High school graduate or GED	27.0 (19.3-36.4)	27.0 (23.0-31.5)	24.1 (22.2-26.1)
Some college	23.3 (15.9-32.8)	22.3 (18.3-26.9)	31.2 (28.5-34.0)
≥College	4.8 (2.4-9.3)	8.1 (5.3-12.2)	32.5 (28.7-36.6)
Family income to poverty ratio^b^			
<1.30	75.5 (62.1-85.2)	100	0
1.30-1.84	12.1 (6.4-21.7)	0	12.4 (10.6-14.5)
1.85-2.99	5.2 (3.3-8.0)	0	22.4 (19.4-25.7)
≥3.00	7.3 (3.1-16.1)	0	65.2 (61.1-69.1)

Abbreviations: GED, general equivalency diploma; SNAP, Supplemental Nutrition Assistance Program.

^a^ Weighted sample size was estimated using National Health and Nutrition Examination Survey dietary weights that account for the complex survey design (including oversampling), survey nonresponse, and poststratification. The weighted sample size represents US adults who are SNAP participants, income-eligible nonparticipants, and higher-income individuals.

^b^ Family income to poverty ratio represents the ratio of family income to the federal poverty threshold, adjusting for household size. For reference, the federal threshold in 2014 for a family of 4 was $23 850 per year. A family of 4 earning $44 123 per year would have a ratio of 1.85. A lower ratio indicates a lower level of income.

**Table 2.** Trends in Dietary Components of the American Heart Association 2020 Strategic Impact Goals by SNAP Participation Status, 2003-2014^a^

Dietary Components and AHA Diet Score Point Value^b^	AHA Intake Goals (Range)	Survey-Weighted Mean Score (95% CI)^a^						*P* Value for Trend	Change From 2003-2014, Mean (95% CI)
		2003-2004 (n = 4066)	2005-2006 (n = 4030)	2007-2008 (n = 4654)	2009-2010 (n = 4996)	2011-2012 (n = 4288)	2013-2014 (n = 4437)		
Total AHA diet score (0-80 points)									
SNAP participants		31.5 (29.2 to 33.8)	30.8 (28.6-32.9)	31.1 (29.5-32.8)	32.8 (31.2-34.5)	33.8 (32.7-34.8)	32.1 (30.6-33.6)	.11	0.57 (−2.18 to 0.33)
Income-eligible nonparticipants		34.2 (32.6-35.9)	34.7 (33.1-36.3)	34.9 (32.7-37.1)	36.3 (34.8-37.9)	36.5 (35.2-37.7)	36.8 (35.3-38.3)	.004	2.56 (0.36 to 4.76)
Higher-income individuals		35.8 (34.6-36.9)	36.4 (35.3-37.4)	36.7 (35.5-37.8)	38.7 (37.9-39.4)	40.0 (38.5-41.5)	39.6 (38.7-40.5)	<.001	3.84 (2.39 to 5.29)
*P* value for interaction								.02^c^	.04^d^
Fruits and vegetables (10 of 80 points)^e^	≥4.5 (0 to ≥4.5 cups/d)								
SNAP participants		3.9 (3.5-4.2)	3.9 (3.5-4.4)	3.9 (3.5-4.3)	4.0 (3.7-4.3)	4.0 (3.8-4.3)	3.7 (3.4-4.0)	.71	−0.18 (−0.63 to 0.28)
Income-eligible nonparticipants		4.6 (4.2-5.1)	4.9 (4.6-5.1)	4.5 (4.2-4.8)	4.7 (4.5-4.9)	4.9 (4.6-5.1)	4.6 (4.2-5.0)	.93	−0.03 (−0.63 to 0.58)
Higher-income individuals		5.2 (4.9-5.5)	5.2 (4.9-5.4)	5.2 (4.9-5.4)	5.4 (5.3-5.5)	5.4 (5.1-5.7)	5.2 (5.0-5.4)	.36	−0.02 (−0.34 to 0.29)
*P* value for interaction								.63^c^	.80^d^
Whole grains (10 of 80 points)	≥3 (0 to ≥3 oz equivalent/d)								
SNAP participants		1.4 (1.0-1.9)	1.5 (1.2-1.9)	1.5 (1.3-1.8)	2.0 (1.7-2.2)	2.4 (2.0-2.8)	2.2 (2.0-2.4)	<.001	0.77 (0.32 to 1.22)
Income-eligible nonparticipants		1.9 (1.5-2.3)	2.1 (1.8-2.5)	2.0 (1.7-2.2)	2.5 (2.1-2.8)	2.7 (2.2-3.1)	2.5 (2.1-2.9)	.004	0.60 (0.03 to 1.18)
Higher-income individuals		2.2 (2.0-2.4)	2.6 (2.4-2.7)	2.6 (2.4-2.8)	3.0 (2.9-3.2)	3.3 (3.1-3.5)	3.2 (3.1-3.4)	<.001	1.02 (0.79 to 1.24)
*P* value for interaction								.38^c^	.25^d^
Fish and shellfish (10 of 80 points)	≥2 (0 to ≥2 oz/d)								
SNAP participants		2.1 (1.6-2.7)	2.0 (1.4-2.6)	1.9 (1.6-2.2)	2.4 (1.8-3.0)	2.1 (1.7-2.6)	1.9 (1.4-2.5)	.93	−0.19 (−0.94 to 0.56)
Income-eligible nonparticipants		2.3 (1.8-2.7)	2.1 (1.7-2.6)	2.0 (1.5-2.5)	2.6 (2.0-3.2)	2.1 (1.7-2.5)	2.2 (1.8-2.6)	.94	−0.10 (−0.71 to 0.51)
Higher-income individuals		2.6 (2.3-3.0)	2.7 (2.5-3.0)	2.6 (2.3-2.9)	2.8 (2.5-3.1)	2.7 (2.3-3.1)	2.7 (2.4-3.1)	.80	0.08 (−0.40 to 0.56)
*P* value for interaction								.93^c^	.65^d^
Sugar-sweetened beverages (10 of 80 points)	≤36 (≤36 to >16 fl oz/wk)								
SNAP participants		3.8 (3.2-4.4)	4.6 (3.8-5.3)	4.3 (3.7-4.9)	4.6 (4.1-5.0)	5.3 (4.8-5.8)	4.8 (4.1-5.5)	.009	0.94 (0.05 to 1.83)
Income-eligible nonparticipants		4.7 (4.1-5.2)	5.5 (4.8-6.2)	5.3 (4.8-5.8)	5.63 (5.11-6.16)	6.0 (5.5-6.4)	6.6 (5.9-7.2)	<.001	1.92 (1.10 to 2.74)
Higher-income individuals		6.0 (5.6-6.4)	6.6 (6.3-6.9)	6.6 (6.3-6.9)	7.1 (6.9-7.3)	7.1 (6.7-7.6)	7.5 (7.1-7.8)	<.001	1.42 (0.89 to 1.94)
*P* value for interaction								.42^c^	.25^d^
Sodium (10 of 80 points)	≤1500 (≤1500 to >4500 mg/d)								
SNAP participants		4.4 (4.0-4.7)	4.1 (3.6-4.7)	3.9 (3.7-4.1)	3.9 (3.7-4.2)	3.9 (3.7-4.2)	4.0 (3.6-4.3)	.18	−0.37 (−0.86 to 0.12)
Income-eligible nonparticipants		3.9 (3.4-4.4)	3.9 (3.5-4.2)	3.8 (3.6-4.1)	3.7 (3.5-4.0)	3.6 (3.4-3.8)	3.8 (3.4-4.1)	.33	−0.13 (−0.73 to 0.47)
Higher-income individuals		3.7 (3.5-3.8)	3.4 (3.2-3.5)	3.4 (3.3-3.5)	3.6 (3.4-3.7)	3.8 (3.7-3.9)	3.7 (3.6-3.9)	.009	0.05 (−0.18 to 0.28)
*P* value for interaction								.02^c^	.04^d^
Nuts, seeds, and legumes (10 of 80 points)	≥4 (0 to ≥4 servings/wk)								
SNAP participants		3.7 (3.3-4.2)	3.6 (2.8-4.4)	3.6 (2.9-4.2)	3.9 (3.5-4.4)	3.9 (3.4-4.5)	4.0 (3.6-4.5)	.20	0.30 (−0.34 to 0.93)
Income-eligible nonparticipants		4.6 (4.1-5.1)	4.1 (3.6-4.5)	4.5 (4.0-5.1)	4.4 (3.9-4.9)	4.6 (4.0-5.2)	4.8 (4.3-5.4)	.20	0.27 (−0.47 to 1.00)
Higher-income individuals		4.4 (4.2-4.7)	4.7 (4.3-5.0)	4.7 (4.4-5.1)	4.8 (4.6-5.1)	5.5 (5.2-5.8)	5.4 (5.1-5.7)	<.001	0.98 (0.61 to 1.36)
*P* value for interaction								.08^c^	.07^d^
Processed meat (10 of 80 points)	≤0.5 (≤0.5 to >1.76 oz/d)								
SNAP participants		7.2 (6.8-7.6)	6.3 (5.5-7.0)	6.6 (6.2-7.1)	6.5 (6.1-7.0)	6.4 (6.0-6.9)	6.2 (5.8-6.7)	.03	−0.94 (−1.55 to −0.34)
Income-eligible nonparticipants		7.1 (6.6-7.6)	6.9 (6.6-7.3)	6.9 (6.3-7.5)	6.9 (6.5-7.3)	6.9 (6.5-7.3)	6.8 (6.4-7.3)	.48	−0.25 (−0.91 to 0.42)
Higher-income individuals		6.6 (6.5-6.8)	6.4 (6.1-6.8)	6.6 (6.4-6.9)	6.6 (6.3-6.9)	6.7 (6.4-7.0)	6.9 (6.6-7.1)	.10	0.20 (−0.12 to 0.52)
*P* value for interaction								.004^c^	.001^d^
Saturated fat (10 of 80 points)	≤7% (≤7% to >15%)								
SNAP participants		5.0 (4.3-5.7)	4.8 (4.3-5.2)	5.5 (5.1-6.0)	5.5 (5.3-5.8)	5.5 (5.2-5.9)	5.3 (4.9-5.6)	.18	0.25 (−0.48 to 0.98)
Income-eligible nonparticipants		5.2 (4.8-5.7)	5.3 (5.0-5.6)	5.8 (5.4-6.3)	5.8 (5.5-6.2)	5.7 (5.3-6.2)	5.5 (5.2-5.8)	.10	0.28 (−0.24 to 0.80)
Higher-income individuals		5.0 (4.7-5.2)	4.8 (4.6-5.0)	5.0 (4.7-5.2)	5.3 (5.1-5.5)	5.5 (5.2-5.7)	5.1 (4.9-5.3)	.005	0.11 (−0.21 to 0.43)
*P* value for interaction								.99^c^	.79^d^

Abbreviations: AHA, American Heart Association; SNAP, Supplemental Nutrition Assistance Program.

^a^ All dietary variables were adjusted for energy to 2000 kcal/d using the residual method prior to analysis. Each AHA dietary component was evaluated based on a continuous scoring system. Intake of each dietary component was scored from 0 to 10 (beneficial components) or from 10 to 0 (harmful components). For beneficial components, individuals with zero intake received the lowest score (0). For harmful components, the lowest score (0) was assigned to a higher level, approximately equivalent to the 80th to 90th percentile of intake among US adults, and rounded to practical values (eg, 4500 mg/d of sodium, one 50-g serving per day of processed meat, two 8-oz servings per day of sugar-sweetened beverages, and 15% energy from saturated fat). Intermediate dietary intake was scored linearly between 0 and 10. For example, an adult consuming 3000 mg/d of sodium would receive 5 sodium points (ie, his or her sodium consumption was halfway between 1500 mg/d and the maximum value of 4500 mg/d).

^b^ The total AHA diet score is the sum of the scores for 8 dietary components (fruits and vegetables; whole grains; fish and shellfish; sugar-sweetened beverages; sodium; nuts, seeds, and legumes; processed meat; and saturated fat).

^c^ *P* value for interaction for potential heterogeneous trends in diet scores by SNAP participation status.

^d^ *P* value for interaction for potential heterogeneous changes in diet scores from 2003 to 2014 by SNAP participation status.

^e^ According to the AHA 2020 goals, up to 3 cups per week (0.42 cups per day) of starchy vegetables (eg, potatoes, peas, corn) could be included; this maximum was incorporated into the analysis, with higher intake not contributing toward the score. Consumption of 100% fruit juice could also be included; however, its contribution was not capped in the original AHA 2020 goals and does not contribute to the score.

Among higher-income individuals, the proportion with an ideal diet score increased from 1.4% (95% CI, 0.8%-2.3%) to 2.6% (95% CI, 2.0%-3.4%) (*P* = .01 for trend) and the proportion with an intermediate diet score increased from 59.1% (95% CI, 55.4%-62.7%) to 68.7% (95% CI, 66.3%-71.0%) (*P *< .001 for trend), while the proportion with a poor diet score decreased from 39.5% (95% CI, 35.9%-43.3%) to 28.7% (95% CI, 26.4%-31.1%) (*P *< .001 for trend) (Table 3 and Figure). In contrast, no significant changes in the proportions having ideal, intermediate, and poor diet scores were detected among SNAP participants and income-eligible nonparticipants. The change in proportions with a poor diet score were −0.5% (95% CI, −12.4% to 11.4%) among SNAP participants, −4.9% (95% CI, −11.6% to 1.9%) among income-eligible nonparticipants, and −10.8% (95% CI, −15.2% to −6.5%) among higher-income individuals (*P* = .06 for interaction). The corresponding changes in proportions with an intermediate diet score were −0.4% (95% CI, −12.2% to 11.3%) among SNAP participants, 3.5% (95% CI, −3.3% to 10.3%) among income-eligible nonparticipants, and 9.6% (95% CI, 5.3%-13.9%) among higher-income individuals (*P* = .05 for interaction). The corresponding changes in proportions with an ideal diet score were 0.9% (95% CI, −0.4% to 2.3%) among SNAP participants, 1.4% (95% CI, −0.4% to 3.2%) among income-eligible nonparticipants, and 1.2% (95% CI, 0.3%-2.2%) among higher-income individuals (*P* = .61 for interaction).

**Table 3.** Trends in Percentage of US Adults Having Poor, Intermediate, and Ideal Diet by SNAP Participation Status, 2003-2014^a^

Diet Scores	Weighted Survey Results, % (95% CI)						*P* Value for Trend	Change in % From 2003-2004 to 2013-2014 (95% CI)
	2003-2004 (n = 4066)	2005-2006 (n = 4030)	2007-2008 (n = 4654)	2009-2010 (n = 4996)	2011-2012 (n = 4288)	2013-2014 (n = 4437)		
**Poor Diet (AHA Diet Score <32.0)**								
SNAP participants	53.9 (43.8-63.8)	55.6 (46.0-64.8)	56.8 (50.8-62.6)	47.7 (42.4-53.0)	45.4 (41.1-49.6)	53.5 (46.9-59.9)	.26	−0.5 (−12.4 to 11.4)
Income-eligible nonparticipants	42.9 (38.2-47.6)	39.4 (32.3-46.9)	41.9 (34.5-49.8)	37.7 (32.8-43.0)	37.2 (31.4-43.5)	38.0 (33.2-43.0)	.13	−4.9 (−11.6 to 1.9)
Higher-income individuals	39.5 (35.9-43.3)	39.6 (35.8-43.6)	36.7 (31.9-41.7)	32.5 (30.0-35.1)	27.4 (24.2-30.9)	28.7 (26.4-31.1)	<.001	−10.8 (−15.2 to −6.5)
*P* value for interaction							.006^b^	.06^c^
**Intermediate Diet (AHA Diet Score = 32.0-63.9)**								
SNAP participants	45.7 (35.9-55.8)	44.1 (34.9-53.7)	42.9 (37.1-49.0)	51.7 (46.7-56.7)	53.7 (49.6-57.8)	45.3 (39.0-51.7)	.35	−0.4 (−12.2 to 11.3)
Income-eligible nonparticipants	56.3 (51.5-61.0)	60.5 (53.0-67.6)	55.9 (49.1-62.6)	61.1 (56.1-65.8)	62.0 (56.0-67.6)	59.8 (54.8-64.6)	.23	3.5 (−3.3 to 10.3)
Higher-income individuals	59.1 (55.4-62.7)	58.6 (54.7-62.3)	61.9 (57.0-66.7)	65.0 (62.4-67.5)	70.1 (67.0-72.9)	68.7 (66.3-71.0)	<.001	9.6 (5.3-13.9)
*P* value for interaction							.008^b^	.05^c^
**Ideal Diet (AHA Diet Score ≥64.0)**								
SNAP participants	0.3 (0-2.2)	0.3 (0-2.1)	0.3 (0.1-0.9)	0.6 (0.2-1.8)	0.9 (0.5-1.7)	1.3 (0.5-3.3)	.07	0.9 (−0.4 to 2.3)
Income-eligible nonparticipants	0.8 (0.3-2.7)	0.1 (0-0.4)	2.1 (0.9-4.9)	1.2 (0.5-3.0)	0.8 (0.3-1.7)	2.2 (1.1-4.4)	.11	1.4 (−0.4 to 3.2)
Higher-income individuals	1.4 (0.8-2.3)	1.8 (1.1-2.8)	1.4 (0.9-2.0)	2.5 (1.8-3.5)	2.5 (1.6-3.9)	2.6 (2.0-3.4)	.01	1.2 (0.3-2.2)
*P* value for interaction							.38^b^	.61^c^

Abbreviations: AHA, American Heart Association; SNAP, Supplemental Nutrition Assistance Program.

^a^ For each continuous score, cut points were defined to correspond to the AHA binary dietary component scoring system. A poor diet was defined as being less than 40% adherence (<32 points). An intermediate diet was defined as adherence to 40% to 79.9% (32.0-63.9 points). An ideal diet was defined as 80% adherence or greater (≥64 points).

^b^ *P* value for interaction for potential heterogeneous trends in percentage of respondents having ideal, intermediate, and poor diet by SNAP participation status.

^c^ *P* value for interaction for potential heterogeneous changes in percentage of respondents having ideal, intermediate, and poor diet by SNAP participation status.

**Figure.**
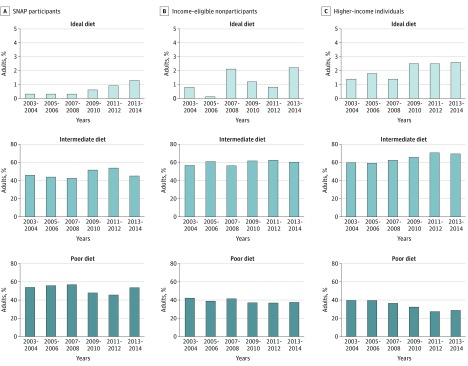
Trends in Ideal, Intermediate, and Poor Diet Among US Adults by Supplemental Nutrition Assistance Program (SNAP) Participation Status Based on American Heart Association Diet Score, 2003 to 2014 From 1999 to 2014, overall diet quality of SNAP participants did not significantly improve, in contrast to both income-eligible nonparticipants and higher-income individuals.

Among the 8 AHA dietary components, disparities worsened for processed meat among SNAP participants. The mean change in processed meat score was −0.94 (95% CI, −1.55 to −0.34), corresponding to a reduced proportion of individuals meeting the recommended intake over time, compared with no change in processed meat score among income-eligible nonparticipants (−0.25; 95% CI, −0.91 to 0.42) and higher-income individuals (0.20; 95% CI, −0.12 to 0.52) (*P* = .001 for interaction) (Table 2). Among other key food groups, dietary disparities also worsened for nuts and seeds, fish and shellfish, and added sugars. For example, SNAP participants experienced the smallest reductions in added sugars (mean change, −4.0 servings per day; 95% CI, −7.3 to −0.7 servings per day) compared with income-eligible nonparticipants (−7.6 servings per day; 95% CI, −10.4 to −4.9 servings per day) and higher-income individuals (−6.0 servings per day; 95% CI, −7.3 to −4.7 servings per day) (*P* = .05 for interaction). Increasing trends of nut and seed and fish and shellfish consumption were apparent among income-eligible nonparticipants and higher-income individuals but not among SNAP participants (eTable 2 and eFigure 1 in the Supplement). Consistent differences for sodium intake were not evident. Disparities appeared to improve for certain nutrients, such as saturated fat, cholesterol, and polyunsaturated fat, but persisted for most food groups and nutrients (eTable 2 and eFigures 1 and 2 in the Supplement). After adjusting for differences in age, sex, race/ethnicity, education, and income over time, diet-related disparities by SNAP participation status were not materially altered for most dietary components (eTables 3, 4, 5, and 6 in the Supplement).

Despite some improvement in diet quality by 2014, SNAP participants still had lower diet quality than income-eligible nonparticipants and higher-income individuals. In 2014, mean (SD) AHA diet scores were 32.1 (11.7) among SNAP participants, 36.8 (12.3) among income-eligible nonparticipants, and 39.6 (10.4) among higher-income individuals (*P* < .001 for difference) (Table 2). Similarly, SNAP participants had a significantly higher proportion with a poor diet score than income-eligible nonparticipants and higher-income individuals (461 of 950 [53.5%] vs 247 of 690 [38.0%] and 773 of 2797 [28.7%]; *P* < .001 for difference) and a significantly lower proportion with an intermediate diet score (477 of 950 [45.3%] vs 428 of 690 [59.8%] and 1933 of 2797 [68.7%]; *P* < .001 for difference). The proportion of participants with an ideal diet score was low in all 3 groups (12 of 950 [1.3%] vs 15 of 690 [2.2%] and 91 of 2797 [2.6%]; *P* = .26 for difference) (Table 3). In terms of AHA diet components, SNAP participants had the lowest consumption of fruits and vegetables; whole grains; fish and shellfish; and nuts, seeds, and legumes and the highest consumption of SSBs, although SNAP participants also had the lowest sodium consumption (Table 4).

**Table 4.** Mean Intake of Key Food Groups and Nutrients Among US Adults by SNAP Participation Status, 2013-2014^a^

Key Food Groups and Nutrients	Survey-Weighted Mean Intake (95% CI)			*P* Value
	SNAP Participants (n = 1108)	Income-Eligible Nonparticipants (n = 799)	Higher-Income Individuals (n = 3086)	
AHA dietary components				
Fruits and vegetables, servings/d	1.31 (1.12-1.50)	1.63 (1.43-1.83)	2.04 (1.93-2.15)	<.001
Whole grains, servings/d	0.66 (0.58-0.73)	0.78 (0.63-0.93)	1.00 (0.96 1.04)	<.001
Fish and shellfish, servings/d	0.16 (0.11-0.22)	0.14 (0.11-0.17)	0.20 (0.16-0.24)	.001
Sugar-sweetened beverages, servings/d	1.87 (1.54-2.20)	1.16 (0.96-1.35)	0.80 (0.70-0.89)	<.001
Sodium, mg/d	3100 (3023-3177)	3239 (3153-3325)	3330 (3274-3385)	<.001
Nuts, seeds, and legumes, servings/d	0.41 (0.29-0.53)	0.53 (0.42-0.64)	0.86 (0.80-0.93)	<.001
Processed meat, servings/d	0.31 (0.24-0.38)	0.26 (0.22-0.29)	0.26 (0.23-0.29)	.30
Saturated fat, % of energy	10.9 (10.58-11.18)	10.9 (10.66-11.18)	11.1 (10.94-11.33)	.18
Other key food groups and nutrients				
Total fruit, servings/d	0.73 (0.63-0.82)	0.88 (0.74-1.02)	0.99 (0.92-1.05)	<.001
Whole fruits	0.47 (0.38-0.57)	0.60 (0.50-0.71)	0.77 (0.70-0.84)	<.001
100% Fruit juice	0.28 (0.23-0.33)	0.32 (0.24-0.39)	0.23 (0.21-0.25)	.02
Total vegetables, servings/d	1.13 (1.01-1.26)	1.33 (1.20-1.45)	1.59 (1.52-1.65)	<.001
Dark green vegetables	0.10 (0.06-0.13)	0.12 (0.09-0.14)	0.19 (0.17-0.22)	<.001
Tomatoes	0.21 (0.19-0.23)	0.27 (0.23-0.30)	0.28 (0.26-0.29)	<.001
Other red and orange vegetables	0.07 (0.05-0.09)	0.08 (0.06-0.10)	0.11 (0.10-0.13)	.02
White potatoes	0.30 (0.26-0.33)	0.30 (0.25-0.34)	0.32 (0.29-0.34)	.58
Other starchy vegetables	0.06 (0.05-0.08)	0.06 (0.04-0.09)	0.07 (0.06-0.08)	.63
Other vegetables	0.37 (0.31-0.44)	0.47 (0.42-0.53)	0.58 (0.54-0.61)	<.001
Vegetables excluding potatoes and starchy	0.77 (0.67-0.88)	0.97 (0.86-1.07)	1.20 (1.13-1.27)	<.001
Nuts and seeds, servings/d	0.35 (0.23-0.48)	0.46 (0.35-0.57)	0.82 (0.75-0.88)	<.001
Legumes, servings/d	0.11 (0.09-0.12)	0.14 (0.13-0.16)	0.09 (0.08-0.10)	<.001
Refined grains, servings/d	4.92 (4.65-5.18)	5.34 (5.06-5.63)	4.96 (4.82-5.11)	.04
Unprocessed red meats, servings/d	0.40 (0.36-0.44)	0.39 (0.35-0.43)	0.42 (0.40-0.44)	.32
Poultry, servings/d	0.42 (0.37-0.47)	0.43 (0.35-0.51)	0.43 (0.40-0.46)	.96
Total dairy, servings/d	1.39 (1.29-1.48)	1.39 (1.26-1.51)	1.46 (1.40-1.51)	.27
Milk	0.60 (0.53-0.67)	0.58 (0.49-0.68)	0.63 (0.59-0.67)	.55
Cheese	0.69 (0.63-0.75)	0.68 (0.59-0.76)	0.69 (0.65-0.73)	.91
Yogurt	0.04 (0.02-0.05)	0.07 (0.04-0.11)	0.08(0.07-0.09)	<.001
Nutrients				
Total fat, % of energy	33.1 (32.6-33.6)	33.4 (32.8-33.9)	34.9 (34.5-35.3)	<.001
Saturated fatty acids, % of energy	10.9 (10.6-11.2)	10.9 (10.7-11.2)	11.1 (10.9-11.3)	.18
Monounsaturated fatty acids, % of energy	13.4 (12.7-14.2)	13.6 (12.9-14.2)	13.6 (13.2-14.0)	.90
Polyunsaturated fatty acids, % of energy	7.6 (7.3-7.9)	7.7 (7.4-8.0)	8.2 (8.1-8.4)	<.001
Seafood ω-3 fatty acids, mg/d	103 (72-134)	79 (63-96)	122 (107-136)	.009
Plant ω-3 fatty acids, mg/d	146 (140-151)	149 (142-155)	166 (161-170)	<.001
Polyunsaturated to saturated fat ratio	0.77 (0.73-0.81)	0.76 (0.73-0.79)	0.80 (0.79-0.82)	.05
Protein, % of energy	15.5 (14.9-16.2)	16.2 (15.5-16.8)	16.6 (16.3-16.9)	<.001
Carbohydrate, % of energy	50.1 (49.3-51.0)	49.3 (48.4-50.2)	46.8 (46.3-47.3)	<.001
Cholesterol, g/d	267 (252-282)	269 (247-290)	278 (271-285)	.39
Fiber, g/d	13.5 (12.5-14.5)	15.7 (14.8-16.7)	17.0 (16.7-17.4)	<.001
Added sugars, tsp equivalent/d	19.9 (18.3-21.6)	15.8 (14.4-17.2)	13.6 (13.1-14.1)	<.001
Potassium, mg/d	2199 (2108-2291)	2373 (2279-2466)	2597 (2551-2642)	<.001
Magnesium, mg/d	248 (233-263)	277 (265-289)	296 (291-301)	<.001
Calcium, mg/d	839 (805-874)	881 (837-925)	911 (894-929)	.004

Abbreviations: AHA, American Heart Association; SNAP, Supplemental Nutrition Assistance Program.

^a^ Most means were adjusted for energy to 2000 kcal/d using the residual method. The means for total fat, saturated fat, monounsaturated fat, polyunsaturated fat, protein, and carbohydrate were adjusted as percentage of total energy.

Similar trends in diet quality were found between SNAP participants with higher vs lower levels of income: neither group had improved AHA diet scores over time. After adjusting for differences in age, sex, and race/ethnicity over time, the mean change in total AHA diet score from 2003 to 2014 was 0.32 (95% CI, −1.95 to 2.58; *P* = .36 for trend) among lower-income SNAP participants (family income to poverty ratio ≤1.30) and −1.00 (95% CI, −4.60 to 2.60; *P* = .33 for trend) among higher-income SNAP participants (family income to poverty ratio >1.30). In contrast, both income-eligible nonparticipants and higher-income individuals had significant improvements in diet quality score over time. The adjusted mean change in total AHA diet score was 2.16 (95% CI, 0.01-4.30; *P* = .008 for trend) and 3.33 (95% CI, 1.86-4.80; *P* < .001 for trend), respectively (*P* = .02 for interaction) (eTable 7 in the Supplement). Further adjustment for education did not alter results. Dietary trends were also similar among subgroups of SNAP participants by age, sex, race/ethnicity, and education (eTables 8, 9, 10, and 11 in the Supplement).

## Discussion

The Supplemental Nutrition Assistance Program served 42.1 million people in fiscal year 2017, with an annual budget of $70 billion, exceeding the budgets of the National Institutes of Health, Centers for Disease Control and Prevention, US Food and Drug Administration, Health Resources and Services Administration, and Agency for Healthcare Research and Quality combined. While SNAP provides essential financial assistance to alleviate hunger and food insecurity in low-income families,^14^ less emphasis has been given to improving diet quality and healthfulness. To our knowledge, the time trends in diet disparities of multiple food groups and nutrients associated with chronic diseases have not been previously evaluated by SNAP participation status. Our results demonstrate that from 1999 to 2014, overall diet quality of SNAP participants did not significantly improve, in contrast to improvements among both income-eligible nonparticipants and higher-income individuals. Consistent with this, disparities in most individual dietary components persisted or worsened over time.

Some improvements were seen in individual dietary components among SNAP participants. These included increased whole grains, whole fruits, and dark green vegetables and decreased SSBs. These encouraging trends could be attributable to public health policies and nutrition education that influence all consumers, including SNAP participants, such as the 5 A Day national campaign,^15^ increasing public support for tax initiatives on SSBs,^16^ and new federal dietary recommendations on whole grains in 2000 and 2005.^17^ Advocacy groups such as the AHA have also launched nutrition campaigns,^18^ while the food industry has increased the number of whole-grain products 20-fold over 10 years.^19^

Despite these positive trends, we found that SNAP participants experienced the smallest improvements in these food categories and also experienced increasing disparities in processed meats, added sugars, nuts and seeds, and fish and shellfish. Our findings highlight the need to investigate and address the reasons for these worsening disparities. For example, access to healthful foods and beverages by low-income families participating in SNAP may remain limited.^20^ Our findings should not be interpreted as a causal effect of participating in SNAP, however. It is possible that dietary trends in this group could have been even worse without participation in SNAP. Nevertheless, our findings underscore the need for robust new strategies to improve diet quality and reduce dietary disparities in the United States.

Nutrition knowledge, often associated with education and income, may partly mediate disparities in dietary intake.^21,22,23^ The USDA SNAP-Ed program provides grants to states for nutrition education and obesity prevention for SNAP participants and other income-eligible individuals, yet represents only about 0.5% of the overall SNAP budget, or about $10 per participant per year. This program could be expanded to address diet-related disparities through not only education but also policy and system interventions that would be rigorously evaluated for effectiveness.

Given higher costs of healthier food,^24^ bipartisan panels have recommended economic incentives for healthier eating among SNAP participants.^25,26^ For example, the Healthy Incentives Pilot randomized trial demonstrated a 26% increase in fruit and vegetable consumption among SNAP participants receiving a 30% subsidy on such purchases.^27^ However, expansion of such programs remains limited, with only about $15 million per year (about $0.36 per participant per year) invested in this program over the last 2 years.^28^ A recent modeling analysis suggested that a 30% subsidy for purchases of fruits and vegetables provided to all SNAP participants could reduce cardiovascular disease disparities between SNAP participants and SNAP-ineligible individuals by approximately 8%.^29^

Revisions of food items eligible for SNAP purchases have also been recommended.^25,26^ A related federal nutrition assistance program, the Special Supplemental Nutrition Program for Women, Infants, and Children (WIC), has been revised to limit eligibility of unhealthy foods and encourage consumption of fruits, vegetables, and whole grains through various measures, including vouchers.^30^ These revisions significantly improved the diet quality of children participating in WIC.^31,32,33^

We found that SNAP participants consumed more SSBs than other US adults, a finding consistent with prior reports^34^ and purchasing data.^35^ About three-quarters of SSB grocery purchases by SNAP participants are paid for with SNAP dollars (with the rest paid for with participants’ other food dollars),^36^ representing 9% of all SNAP spending^37^ ($6 billion per year^38^) or about 36 million 8-oz servings per day of SSBs purchased by SNAP.^39^ While removing SSBs from SNAP eligibility is controversial because of concerns about government paternalism and industry opposition,^40,41^ most SNAP participants support removing SSBs from eligible purchases if paired with incentives for healthful foods.^42^ As an alternative strategy, partial disincentives on purchases of unhealthful items, rather than complete restrictions, would preserve choice and also provide direct cost savings to SNAP that could be leveraged for subsidizing healthful foods.

Our investigation has several strengths. Eight cycles of nationally representative data provided the most up-to-date evaluation of recent trends. We assessed diet quality scores, proportions meeting cut points, and intakes of key food groups and nutrients, providing a comprehensive portrait of diet quality of US adults by SNAP status.

### Limitations

Potential limitations should also be considered. Self-reported dietary intake is subject to measurement error. However, NHANES incorporated 1 or 2 standardized 24-hour diet recalls per person that were energy adjusted and averaged whenever possible to reduce measurement error.^43,44,45^ Although one or two 24-hour diet recalls per individual may inaccurately estimate habitual long-term intake for a specific individual, this method is excellent for estimating the mean intake of a group or population stratum,^43^ the unit in our analyses. Benefit levels in SNAP can differ between participants and over time, producing partial misclassification of SNAP participation and reducing our ability to detect full differences. There could also be misreporting of SNAP participation, although the proportion of misreporting appeared to be a small fraction.^46^ The SNAP participation rates reported by NHANES respondents were consistent with the participation rates assessed based on the administrative records of the USDA.^47,48,49^ Because the consequence of such measurement error is to bias the results toward the null, our findings may underestimate certain disparities by SNAP status. Lastly, the proportion of SNAP participants among US adults increased from 9% in 1999 to 17% in 2014, which could reflect both changes in economic factors (ie, the Great Recession) and, to a lesser extent, changes in SNAP policies, such as the 2002 and 2008 Farm Acts that improved accessibility and expanded eligibility.^50^ These economic and policy changes moved individuals with greater education and higher income onto SNAP. Such shifts would most likely make the observed dietary disparities smaller, because such individuals would generally have better diet quality than SNAP participants with lower education. The observed dietary disparities among US adults could be even larger.

## Conclusions

Despite an overall improvement in diet quality among all US adults aged 20 years or older between 1999 and 2014, the overall diet quality of SNAP participants remained unchanged, and disparities persisted or worsened for most dietary components compared with income-eligible nonparticipants and higher-income individuals. Our findings highlight the need for evidence-based nutrition policies to help close these gaps and reduce diet-related health disparities in the United States.

## Notes

Supplement. **eTable 1.** Trends in SNAP Participation and Sociodemographic Characteristics of US Adults by SNAP Participation Status, 1999-2014 **eTable 2.** Trends in Dietary Intake of Key Food Groups and Nutrients Among US Adults by SNAP Participation Status, 1999-2014 **eTable 3.** Adjusted Trends in the American Heart Association (AHA) 2020 Diet Score by SNAP Participation Status, 2003-2014 **eTable 4.** Adjusted Trends in AHA Diet Score Among US Adults by SNAP Participation Status, 2003-2014 **eTable 5.** Adjusted Trends in Percentages of Having Poor, Intermediate, and Ideal Diet Among US Adults by SNAP Participation Status, 2003-2014 **eTable 6.** Adjusted Trends in Dietary Intake of Key Food Groups and Nutrients Among US Adults by SNAP Participation Status, 1999-2014 **eTable 7.** Crude and Adjusted Trends in the American Heart Association (AHA) 2020 Diet Score by SNAP Participation Status, 2003-2014 **eTable 8.** Mean Change in Dietary Components of the American Heart Association (AHA) 2020 Strategic Impact Goals Among SNAP Participants From 1999-2002 to 2011-2014, by Age **eTable 9.** Mean Change in Dietary Components of the American Heart Association (AHA) 2020 Strategic Impact Goals Among SNAP Participants From 1999-2002 to 2011-2014, by Gender **eTable 10.** Mean Change in Dietary Components of the American Heart Association (AHA) 2020 Strategic Impact Goals Among SNAP Participants From 1999-2002 to 2011-2014, by Race/Ethnicity **eTable 11.** Mean Change in Dietary Components of the American Heart Association (AHA) 2020 Strategic Impact Goals Among SNAP Participants From 1999-2002 to 2011-2014, by Education **eFigure 1.** Percent Change in Intake of AHA Dietary Components and Other Key Food Groups and Nutrients Among US Adults by SNAP Participation Status From 1999-2000 to 2013-2014 **eFigure 2.** Percent of US Adults Meeting Dietary Recommendations for AHA 2020 Strategic Goals and Other Key Food Groups and Nutrients by SNAP Participation Status, 2013-2014
