# Supplementary material for: Trends and Disparities in Diet Quality Among US Adults by Supplemental Nutrition Assistance Program Participation Status
Source: JAMA Netw Open. 2018 Jun 15;1(2):e180237. doi: 10.1001/jamanetworkopen.2018.0237 (PMC6258006; doi:10.1001/jamanetworkopen.2018.0237)
Supplement: Supplement. — eTable 1. Trends in SNAP Participation and Sociodemographic Characteristics of US Adults by SNAP Participation Status, 1999-2014 eTable 2. Trends in Dietary Intake of Key Food Groups and Nutrients Among US Adults by SNAP Participation Status, 1999-2014 eTable 3. Adjusted Trends in the American Heart Association (AHA) 2020 Diet Score by SNAP Participation Status, 2003-2014 eTable 4. Adjusted Trends in AHA Diet Score Among US Adults by SNAP Participation Status, 2003-2014 eTable 5. Adjusted Trends in Percentages of Having Poor, Intermediate, and Ideal Diet Among US Adults by SNAP Participation Status, 2003-2014 eTable 6. Adjusted Trends in Dietary Intake of Key Food Groups and Nutrients Among US Adults by SNAP Participation Status, 1999-2014 eTable 7. Crude and Adjusted Trends in the American Heart Association (AHA) 2020 Diet Score by SNAP Participation Status, 2003-2014 eTable 8. Mean Change in Dietary Components of the American Heart Association (AHA) 2020 Strategic Impact Goals Among SNAP Participants From 1999-2002 to 2011-2014, by Age eTable 9. Mean Change in Dietary Components of the American Heart Association (AHA) 2020 Strategic Impact Goals Among SNAP Participants From 1999-2002 to 2011-2014, by Gender eTable 10. Mean Change in Dietary Components of the American Heart Association (AHA) 2020 Strategic Impact Goals Among SNAP Participants From 1999-2002 to 2011-2014, by Race/Ethnicity eTable 11. Mean Change in Dietary Components of the American Heart Association (AHA) 2020 Strategic Impact Goals Among SNAP Participants From 1999-2002 to 2011-2014, by Education eFigure 1. Percent Change in Intake of AHA Dietary Components and Other Key Food Groups and Nutrients Among US Adults by SNAP Participation Status From 1999-2000 to 2013-2014 eFigure 2. Percent of US Adults Meeting Dietary Recommendations for AHA 2020 Strategic Goals and Other Key Food Groups and Nutrients by SNAP Participation Status, 2013-2014 [file jamanetwopen-1-e180237-s001.pdf]

Supplementary Online Content

Zhang FF, Liu J, Rehm CD, Wilde P, Mande JR, Mozaffarian D. Trends and disparities in diet quality among US adults by Supplemental Nutrition Assistance Program participation status. *JAMA Netw Open*. 2018;1(2):e180237. doi:10.1001/jamanetworkopen.2018.0237

eTable 1. Trends in SNAP Participation and Sociodemographic Characteristics of US Adults by SNAP Participation Status, 1999-2014

eTable 2. Trends in Dietary Intake of Key Food Groups and Nutrients Among US Adults by SNAP Participation Status, 1999-2014

eTable 3. Adjusted Trends in the American Heart Association (AHA) 2020 Diet Score by SNAP Participation Status, 2003-2014

eTable 4. Adjusted Trends in AHA Diet Score Among US Adults by SNAP Participation Status, 2003-2014

eTable 5. Adjusted Trends in Percentages of Having Poor, Intermediate, and Ideal Diet Among US Adults by SNAP Participation Status, 2003-2014

eTable 6. Adjusted Trends in Dietary Intake of Key Food Groups and Nutrients Among US Adults by SNAP Participation Status, 1999-2014

eTable 7. Crude and Adjusted Trends in the American Heart Association (AHA) 2020 Diet Score by SNAP Participation Status, 2003-2014

eTable 8. Mean Change in Dietary Components of the American Heart Association (AHA) 2020 Strategic Impact Goals Among SNAP Participants From 1999-2002 to 2011-2014, by Age

eTable 9. Mean Change in Dietary Components of the American Heart Association (AHA) 2020 Strategic Impact Goals Among SNAP Participants From 1999-2002 to 2011-2014, by Gender

eTable 10. Mean Change in Dietary Components of the American Heart Association (AHA) 2020 Strategic Impact Goals Among SNAP Participants From 1999-2002 to 2011-2014, by Race/Ethnicity

eTable 11. Mean Change in Dietary Components of the American Heart Association (AHA) 2020 Strategic Impact Goals Among SNAP Participants From 1999-2002 to 2011-2014, by Education

eFigure 1. Percent Change in Intake of AHA Dietary Components and Other Key Food Groups and Nutrients Among US Adults by SNAP Participation Status From 1999-2000 to 2013-2014

eFigure 2. Percent of US Adults Meeting Dietary Recommendations for AHA 2020 Strategic Goals and Other Key Food Groups and Nutrients by SNAP Participation Status, 2013-2014

© 2018 Zhang FF et al. *JAMA Network Open.*

This supplementary material has been provided by the authors to give readers additional information about their work.

eTable 1. Trends in SNAP Participation and Sociodemographic Characteristics of U.S. Adults by SNAP Participation Status, 1999-2014

Characteristics	1999-2000	2001-2002	2003-2004	2005-2006	2007-2008	2009-2010	2011-2012	2013-2014	*P* for trend
				N, Survey-Weighted % (95% CI)					
**SNAP participation status**									
SNAP participants	423<br>8.6 (6.5, 11.4)	417<br>8.1 (6.2, 10.4)	573<br>10.5 (8.2, 13.3)	490<br>8.0 (6.2, 10.3)	858<br>11.5 (9.2, 14.2)	1,158<br>14.1 (11.4, 17.2)	1,135<br>17.4 (13.7, 21.9)	1,108<br>17.3 (14.3, 20.8)	<0.001
Income-eligible nonparticipants	825<br>15.3 (11.5, 20.1)	884<br>14.3 (12.4, 16.6)	784<br>12.9 (10.8, 15.2)	788<br>12.0 (10.3, 14.0)	882<br>11.4 (9.5, 13.6)	945<br>11.1 (9.4, 13.2)	785<br>12.9 (10.8, 15.3)	799<br>12.3 (10.4, 14.6)	0.08
Higher-Income Individuals	2984<br>76.0 (70.7, 80.7)	3,443<br>77.6 (74.9, 80.1)	3,029<br>76.6 (72.1, 80.6)	3,195<br>80.0 (76.9, 82.7)	3,644<br>77.1 (73.1, 80.7)	3,607<br>74.8 (71.8, 77.5)	2,854<br>69.7 (64.3, 74.7)	3,086<br>70.3 (65.0, 75.2)	0.003
				Survey-Weighted % (95% CI)					
**Age,** *y*, Mean (95% CI)									
SNAP participants	41.4<br>(39.0, 43.8)	39.0<br>(36.8, 41.2)	40.3<br>(38.4, 42.3)	39.3<br>(37.2, 41.3)	40.4<br>(38.7, 42.1)	41.8<br>(41.0, 42.6)	42.9<br>(41.3, 44.6)	42.5<br>(40.8, 44.2)	0.003
Income-eligible nonparticipants	44.0<br>(40.8, 47.2)	45.1<br>(43.2, 47.0)	45.1<br>(43.3, 46.8)	47.7<br>(44.9, 50.4)	45.1<br>(41.7, 48.4)	43.8<br>(41.1, 46.5)	43.6<br>(35.6, 51.6)	44.8<br>(42.9, 46.8)	0.80
Higher-Income Individuals	45.8<br>(44.7, 46.8)	46.3<br>(45.1, 47.5)	47.6<br>(46.5, 48.8)	47.5<br>(46.1, 48.9)	47.9<br>(47.0, 48.8)	48.5<br>(47.5, 49.5)	49.2<br>(47.6, 50.8)	49.2<br>(48.2, 50.3)	<0.001
**% Male**									
SNAP participants	36.6<br>(30.8, 42.4)	36.3<br>(30.8, 41.8)	36.5<br>(32.7, 40.3)	36.9<br>(32.0, 41.7)	39.6<br>(35.4, 43.8)	42.7<br>(40.4, 45.1)	46.6<br>(42.9, 50.3)	45.2<br>(41.7, 48.6)	<0.001
Income-eligible nonparticipants	42.4<br>(38.4, 46.6)	43.1<br>(38.2, 48.1)	45.2<br>(41.4,49.2)	46.9<br>(42.4, 51.4)	42.4<br>(38.8, 46.1)	46.6<br>(43.2, 50.0)	47.4<br>(43.9, 51.0)	47.5<br>(44.4, 50.7)	0.02
Higher-Income Individuals	50.1<br>(47.7, 52.5)	50.4<br>(48.7, 52.1)	50<br>(48.1,51.8)	49.2<br>(47.6, 50.8)	48.9<br>(47.6, 50.1)	49.4<br>(47.7, 51.0)	49.4<br>(47.0, 51.8)	49.5<br>(47.6, 51.3)	0.40
**% Non-Hispanic White**									
SNAP participants	45.8<br>(36.1, 55.6)	47.2<br>(36.0, 58.5)	51.6<br>(35.3, 67.9)	48.1<br>(36.8, 59.4)	46.1<br>(28.4, 63.8)	46.5<br>(34.2, 58.8)	48.5<br>(35.3, 61.8)	49.9<br>(39.6, 60.2)	0.81
Income-eligible nonparticipants	58.2<br>(45.4, 70.0)	55.7<br>(45.1, 65.8)	60<br>(44.5,73.8)	57.6<br>(48.7, 66.0)	56.7<br>(43.8, 68.7)	50.1<br>(41.9, 58.4)	57<br>(43.8, 69.3)	52.0<br>(40.2, 63.5)	0.42
Higher-Income Individuals	75.9<br>(72.0, 79.4)	77.8<br>(73.5, 81.5)	78.1<br>(72.1,83.2)	77.7<br>(72.0, 82.4)	75.8<br>(69.3, 81.3)	75.8<br>(69.2, 81.3)	72.8<br>(65.9, 78.8)	72.2<br>(66.4, 77.3)	0.06
**% Non-Hispanic Black**									
SNAP participants	19.6<br>(10.4, 28.8)	29.1<br>(17.1, 41.1)	28.7<br>(20.0, 37.4)	33.2<br>(24.0, 42.3)	26.9<br>(15.3, 38.5)	24.4<br>(19.4, 29.5)	25.2<br>(15.9, 34.6)	22.0<br>(15.8, 28.2)	0.48
Income-eligible nonparticipants	12.0<br>(5.99, 22.6)	14.8<br>(10.6, 20.4)	12.0<br>(8.37, 16.9)	13.7<br>(8.58, 21.1)	10.9<br>(6.96, 16.7)	13.6<br>(10.6, 17.2)	10.7<br>(5.24, 20.7)	12.6<br>(8.44, 18.5)	0.73
Higher-Income Individuals	9.52<br>(7.28, 12.4)	8.33<br>(5.70, 12.0)	8.63<br>(6.01,12.2)	9.06<br>(6.20, 13.05)	8.97<br>(6.15, 12.9)	8.60<br>(6.86, 10.74)	8.16<br>(5.40, 12.15)	8.58<br>(6.43, 11.38)	0.69
**% Hispanic**									
SNAP participants	9.6<br>(2.50, 16.6)	6.6<br>(2.1, 11.2)	12.2<br>(3.10, 21.3)	10.6<br>(5.00, 16.2)	14.9<br>(6.10, 23.6)	14.8<br>(6.40, 23.1)	9.4<br>(2.10, 16.6)	13.4<br>(8.5, 18.3)	0.35
Income-eligible	10.8	13.5	13.6	20.4	18.5	18.2	14.3	18.2	0.13

© 2018 Zhang FF et al. *JAMA Network Open.*

nonparticipants	(6.50, 17.6)	(9.2, 19.4)	(6.80, 25.6)	(15.4, 26.4)	(11.4, 28.7)	(11.0, 28.4)	(8.50, 23.1)	(12.0, 26.7)	
Higher-Income	5.09	6.06	6.17	5.54	5.92	5.78	6.47	6.30	0.58
Individuals	(3.42, 7.52)	(4.53, 8.06)	(3.66, 10.2)	(4.22, 7.23)	(4.68, 7.47)	(3.50, 9.39)	(3.79, 10.8)	(3.89, 10.1)	
**% High school or less than high school education**									
SNAP	78.4	71.9	70.3	65.8	76.8	71.9	58	62.3	<0.001
participants	(71.0, 84.3)	(63.5, 79.0)	(62.8, 76.8)	(58.7, 72.3)	(72.2, 80.8)	(67.1, 76.3)	(51.9, 63.8)	(58.8, 65.7)	
Income-eligible	66.3	69.6	62.6	64.6	66.7	59.5	51.5	52	0.002
nonparticipants	(56.4, 74.9)	(65.5, 73.4)	(55.1, 69.4)	(56.9, 71.6)	(57.6, 74.6)	(51.6, 66.9)	(36.7, 66.0)	(43.8, 60.1)	
Higher-Income	43.7	36.3	37.9	36.6	38.3	32.7	27.7	28.1	<0.001
Individuals	(39.6, 47.9)	(33.3, 39.4)	(34.5, 41.5)	(32.5, 40.9)	(33.9, 42.8)	(29.8, 35.6)	(22.4, 33.7)	(24.9, 31.5)	
**% Some college, college or above education**									
SNAP	21.6	28.1	29.7	34.2	23.2	28.1	42	37.7	<0.001
participants	(15.7, 29.0)	(21.0, 36.5)	(23.2, 37.2)	(27.7, 41.3)	(19.2, 27.8)	(23.7, 32.9)	(36.2, 48.1)	(34.3, 41.2)	
Income-eligible	33.7	30.4	37.4	35.4	33.3	40.5	48.5	48	0.002
nonparticipants	(25.1, 43.6)	(26.6, 34.5)	(30.6, 44.9)	(28.4, 43.1)	(25.4, 42.4)	(33.1, 48.4)	(34.0, 63.3)	(39.9, 56.2)	
Higher-Income	56.3	63.7	62.1	63.4	61.7	67.3	72.3	71.9	<0.001
Individuals	(52.1, 60.4)	(60.6, 66.7)	(58.5, 65.5)	(59.1, 67.5)	(57.2, 66.1)	(64.4, 70.2)	(66.3, 77.6)	(68.5, 75.1)	
**Family income to poverty ratio (FIPR)**[a], mean (95% CI)									
SNAP	1.07	1.11	1.06	1.46	0.96	1.12	1.25	1.21	0.11
participants	(0.86, 1.29)	(0.92, 1.30)	(0.94, 1.18)	(1.22, 1.68)	(0.86, 1.06)	(0.86, 1.38)	(1.13, 1.37)	(1.11, 1.32)	
Income-eligible	0.83	0.81	0.90	0.86	0.85	0.81	0.80	0.92	0.46
nonparticipants	(0.77, 0.90)	(0.78, 0.85)	(0.84, 0.96)	(0.83, 0.90)	(0.80, 0.89)	(0.78, 0.85)	(0.69, 0.92)	(0.88, 0.97)	
Higher-Income	3.59	3.66	3.60	3.60	3.66	3.69	3.71	3.69	0.16
Individuals	(3.42, 3.76)	(3.56, 3.76)	(3.50, 3.70)	(3.50, 3.70)	(3.52, 3.79)	(3.60, 3.77)	(3.56, 3.86)	(3.57, 3.81)	
**% Lower-income (FIPR <1.30)**									
SNAP	74.7	75.5	73.2	62.6	79.8	73.0	64.7	69.2	0.16
participants	(60.8, 88.5)	(64.6, 86.3)	(64.5, 82.0)	(52.3, 72.9)	(73.7, 85.9)	(64.1, 81.8)	(57.0, 72.3)	(63.7, 74.7)	
Income-eligible nonparticipants	1	1	1	1	1	1	1	1	
Higher-Income Individuals	0	0	0	0	0	0	0	0	
**% Higher-income (FIPR ≥1.30)**									
SNAP	25.3	24.5	26.8	37.4	20.2	27	35.3	30.8	0.16
participants	(14.0, 41.3)	(15.3, 36.9)	(19.0, 36.4)	(27.8, 48.1)	(14.8, 26.9)	(19.1, 36.7)	(28.1, 43.3)	(25.6, 36.5)	
Income-eligible nonparticipants	0	0	0	0	0	0	0	0	
Higher-Income Individuals	1	1	1	1	1	1	1	1	

Abbreviations: SNAP: Supplemental Nutrition Assistance Program; FIPR: Family income to poverty ratio

[a.] Family income to poverty ratio represents the ratio of family income to the federal poverty threshold, adjusting for household size. For reference, the federal threshold in 2014 for a family of 4 was $23,850/y. A family of 4 earning $44,123/y would have a ratio of 1.85. A lower ratio indicates a lower level of income.

© 2018 Zhang FF et al. *JAMA Network Open.*

eTable 2. Trends in Dietary Intake of Key Food Groups and Nutrients Among U.S. Adults by SNAP Participation Status, 1999-2014

Dietary Intake	Survey-Weighted Mean Intake (95% CI)[a]								P for Trend	Mean Change From 1999-2000 To 2013-2014 (95% CI)
	1999-2000 (n=423)	2001-2002 (n=417)	2003-2004 (n=573)	2005-2006 (n=490)	2007-2008 (n=858)	2009-2010 (n=1158)	2011-2012 (n=1135)	2013-2014 (n=1108)		
**Total fruits, servings/d**										
SNAP participants	0.75 (0.59, 0.91)	0.79 (0.63, 0.95)	0.84 (0.68,1.00)	0.83 (0.67, 1.00)	0.76 (0.63, 0.90)	0.91 (0.81, 1.01)	0.90 (0.80, 0.99)	0.73 (0.64, 0.82)	0.79	-0.02 (-0.20, 0.16)
Income-eligible nonparticipants	1.00 (0.78, 1.22)	0.87 (0.70, 1.04)	0.93 (0.78, 1.08)	1.01 (0.95, 1.06)	0.93 (0.81 ,1.04)	1.01 (0.91, 1.11)	1.03 (0.93, 1.13)	0.88 (0.75, 1.01)	0.91	-0.12 (-0.37, 0.14)
Higher-Income Individuals	1.02 (0.88, 1.15)	1.13 (1.04, 1.22)	1.03 (0.91, 1.15)	1.00 (0.93,1.08)	1.05 (0.95, 1.15)	1.12 (1.08 ,1.17)	1.09 (1.00, 1.17)	0.99 (0.92, 1.05)	0.85	-0.03 (-0.18, 0.12)
									0.91[b]	0.71[b]
**Whole fruits, servings/d**										
SNAP participants	0.35 (0.23, 0.47)	0.38 (0.31, 0.44)	0.40 (0.33, 0.48)	0.39 (0.28, 0.49)	0.46 (0.36, 0.55)	0.51 (0.45, 0.58)	0.53 (0.45, 0.62)	0.47 (0.38, 0.56)	0.002	0.12 (-0.02, 0.27)
Income-eligible nonparticipants	0.57 (0.43, 0.70)	0.54 (0.41, 0.67)	0.57 (0.46, 0.68)	0.64 (0.60, 0.69)	0.59 (0.50, 0.67)	0.64 (0.56 ,0.72)	0.66 (0.58, 0.74)	0.60 (0.50, 0.70)	0.16	0.04 (-0.12, 0.2)
Higher-Income Individuals	0.62 (0.54, 0.71)	0.67 (0.63, 0.72)	0.66 (0.58, 0.73)	0.66 (0.60, 0.72)	0.75 (0.68, 0.82)	0.83 (0.80, 0.86)	0.81 (0.74 ,0.89)	0.77 (0.71, 0.83)	<0.001	0.15 (0.04, 0.25)
									0.22[b]	0.47[b]
**100% fruit juices, servings/d**										
SNAP participants	0.41 (0.33, 0.49)	0.50 (0.36, 0.65)	0.46 (0.37, 0.55)	0.68 (0.21, 1.14)	0.36 (0.26, 0.46)	0.41 (0.37, 0.45)	0.42 (0.34, 0.50)	0.28 (0.23, 0.32)	0.002	-0.13 (-0.23, -0.04)
Income-eligible nonparticipants	0.51 (0.35, 0.66)	0.38 (0.29, 0.46)	0.38 (0.31, 0.46)	0.36 (0.33, 0.40)	0.39 (0.32, 0.46)	0.42 (0.35, 0.48)	0.38 (0.27, 0.49)	0.32 (0.25, 0.39)	0.08	-0.19 (-0.36, -0.02)
Higher-Income Individuals	0.42 (0.35, 0.49)	0.43 (0.38, 0.47)	0.38 (0.33, 0.44)	0.36 (0.32, 0.39)	0.32 (0.28, 0.36)	0.32 (0.28, 0.35)	0.27 (0.23, 0.32)	0.23 (0.21, 0.25)	<0.001	-0.19 (-0.26, -0.12)
									0.30[b]	0.46[b]
**Total vegetables, servings/d**										
SNAP participants	1.37 (1.11, 1.63)	1.29 (1.15, 1.43)	1.34 (1.21, 1.48)	1.33 (1.18, 1.49)	1.34 (1.21, 1.47)	1.24 (1.18, 1.30)	1.32 (1.24, 1.41)	1.13 (1.02, 1.25)	0.05	-0.23 (-0.51, 0.05)
Income-eligible nonparticipants	1.41 (1.27, 1.54)	1.46 (1.30, 1.63)	1.53 (1.45, 1.61)	1.53 (1.37, 1.69)	1.46 (1.35, 1.57)	1.47 (1.38, 1.55)	1.52 (1.45, 1.59)	1.33 (1.21, 1.44)	0.55	-0.08 (-0.26, 0.1)
Higher-Income Individuals	1.68 (1.60, 1.76)	1.64 (1.57, 1.71)	1.68 (1.64, 1.72)	1.68 (1.60, 1.75)	1.67 (1.61, 1.73)	1.71 (1.64, 1.77)	1.73 (1.64, 1.81)	1.59 (1.53, 1.65)	0.80	-0.09 (-0.19, 0.01)
									0.22[b]	0.63[b]
**Dark-green vegetables, servings/d**										
SNAP participants	0.06 (0.03, 0.08)	0.05 (0.03, 0.08)	0.05 (0.03, 0.07)	0.11 (0.05, 0.17)	0.07 (0.06, 0.09)	0.09 (0.07, 0.10)	0.09 (0.06, 0.12)	0.10 (0.06, 0.13)	0.006	0.04 (0.00, 0.08)
Income-eligible nonparticipants	0.05 (0.03, 0.07)	0.07 (0.05, 0.10)	0.09 (0.06, 0.11)	0.10 (0.07, 0.13)	0.09 (0.06, 0.12)	0.10 (0.06, 0.13)	0.12 (0.08, 0.16)	0.12 (0.09, 0.14)	<0.001	0.06 (0.03, 0.09)
Higher-Income Individuals	0.14 (0.10, 0.17)	0.13 (0.11, 0.16)	0.13 (0.11, 0.14)	0.15 (0.13, 0.17)	0.15 (0.13, 0.17)	0.16 (0.15, 0.17)	0.18 (0.15, 0.22)	0.19 (0.17, 0.21)	<0.001	0.06 (0.01, 0.10)
									0.62[b]	0.57[b]
**Tomatoes, servings/d**										

© 2018 Zhang FF et al. *JAMA Network Open.*

SNAP participants	0.23 (0.17, 0.28)	0.32 (0.25, 0.38)	0.31 (0.26, 0.37)	0.26 (0.22, 0.29)	0.27 (0.24, 0.30)	0.25 (0.23, 0.28)	0.27 (0.24, 0.30)	0.21 (0.19, 0.23)	0.02	-0.02 (-0.07, 0.04)
Income-eligible nonparticipants	0.26 (0.21, 0.32)	0.33 (0.27, 0.40)	0.35 (0.29, 0.42)	0.32 (0.28, 0.36)	0.28 (0.26, 0.30)	0.28 (0.25, 0.31)	0.31 (0.29, 0.33)	0.27 (0.23, 0.30)	0.27	0.00 (-0.06, 0.06)
Higher-Income Individuals	0.31 (0.28, 0.34)	0.34 (0.31, 0.38)	0.36 (0.33, 0.40)	0.32 (0.30, 0.35)	0.32 (0.31, 0.34)	0.31 (0.27, 0.34)	0.31 (0.29, 0.33)	0.28 (0.26, 0.29)	<0.001	-0.04 (-0.07, 0.00)
									0.67 [b]	0.43 [b]
**Other red/orange vegetables, servings/d**										
SNAP participants	0.08 (0.04, 0.11)	0.06 (0.04, 0.08)	0.05 (0.03, 0.06)	0.06 (0.04, 0.08)	0.05 (0.04, 0.06)	0.06 (0.05, 0.07)	0.07 (0.05, 0.09)	0.07 (0.05, 0.09)	0.35	-0.01 (-0.05, 0.04)
Income-eligible nonparticipants	0.08 (0.05, 0.10)	0.08 (0.06, 0.09)	0.09 (0.06, 0.11)	0.08 (0.06, 0.10)	0.09 (0.06, 0.12)	0.09 (0.08, 0.10)	0.09 (0.07, 0.12)	0.08 (0.06, 0.10)	0.33	0.00 (-0.03, 0.04)
Higher-Income Individuals	0.09 (0.08, 0.11)	0.09 (0.07, 0.10)	0.08 (0.07, 0.08)	0.09 (0.08, 0.10)	0.09 (0.08, 0.10)	0.11 (0.10, 0.12)	0.12 (0.11, 0.13)	0.11 (0.10, 0.13)	<0.001	0.02 (0.00, 0.04)
									0.10 [b]	0.37 [b]
**White potatoes, servings/d**										
SNAP participants	0.45 (0.36, 0.54)	0.33 (0.25, 0.41)	0.44 (0.38, 0.50)	0.37 (0.28, 0.45)	0.38 (0.33, 0.42)	0.33 (0.30, 0.36)	0.32 (0.28, 0.36)	0.30 (0.26, 0.33)	<0.001	-0.15 (-0.24, -0.06)
Income-eligible nonparticipants	0.38 (0.29, 0.46)	0.36 (0.28, 0.44)	0.40 (0.35, 0.45)	0.35 (0.29, 0.41)	0.35 (0.29, 0.42)	0.33 (0.28, 0.39)	0.35 (0.29, 0.40)	0.30 (0.26, 0.34)	0.06	-0.08 (-0.17, 0.01)
Higher-Income Individuals	0.38 (0.34, 0.43)	0.37 (0.35, 0.39)	0.37 (0.34, 0.41)	0.35 (0.32, 0.39)	0.37 (0.33, 0.40)	0.36 (0.34, 0.38)	0.32 (0.29, 0.34)	0.32 (0.29, 0.34)	<0.001	-0.07 (-0.12, -0.01)
									0.14 [b]	0.21 [b]
**Other starchy vegetables, servings/d**										
SNAP participants	0.09 (0.05, 0.14)	0.09 (0.05, 0.13)	0.07 (0.05, 0.10)	0.08 (0.05, 0.10)	0.09 (0.07, 0.10)	0.09 (0.07, 0.11)	0.10 (0.06 ,0.13)	0.06 (0.05, 0.08)	0.49	-0.03 (-0.07, 0.01)
Income-eligible nonparticipants	0.10 (0.06, 0.14)	0.10 (0.08, 0.13)	0.07 (0.05, 0.09)	0.09 (0.07, 0.11)	0.09 (0.07, 0.11)	0.10 (0.07, 0.13)	0.09 (0.06, 0.11)	0.06 (0.04, 0.08)	0.16	-0.04 (-0.08, 0.01)
Higher-Income Individuals	0.10 (0.08, 0.11)	0.09 (0.08, 0.10)	0.08 (0.07, 0.09)	0.10 (0.08, 0.11)	0.09 (0.08, 0.11)	0.09 (0.07, 0.10)	0.08 (0.07, 0.09)	0.07 (0.06, 0.08)	0.003	-0.03 (-0.04, -0.01)
									0.90 [b]	0.88 [b]
**Vegetables, excluding potatoes/starchy, servings/d**										
SNAP participants	0.82 (0.63, 1.02)	0.87 (0.75, 1.00)	0.83 (0.73, 0.93)	0.89 (0.75, 1.03)	0.88 (0.77, 0.98)	0.82 (0.75, 0.89)	0.91 (0.83, 0.99)	0.77 (0.68, 0.87)	0.66	-0.05 (-0.27, 0.17)
Income-eligible nonparticipants	0.93 (0.83, 1.03)	1.00 (0.85, 1.15)	1.06 (0.95, 1.16)	1.09 (0.96, 1.21)	1.02 (0.92, 1.12)	1.03 (0.96, 1.11)	1.08 (0.97, 1.20)	0.97 (0.87, 1.06)	0.42	0.04 (-0.10, 0.18)
Higher-Income Individuals	1.20 (1.10, 1.29)	1.18 (1.11, 1.24)	1.22 (1.17, 1.27)	1.23 (1.15, 1.30)	1.21 (1.15, 1.28)	1.26 (1.20, 1.32)	1.33 (1.23, 1.42)	1.20 (1.13, 1.27)	0.10	0.00 (-0.11, 0.12)
									0.40 [b]	0.83 [b]
**Total grains, servings/d**										
SNAP participants	5.59 (5.15, 6.04)	6.34 (5.72, 6.96)	6.15 (5.88, 6.41)	5.97 (5.61, 6.32)	6.20 (5.89, 6.50)	6.32 (6.10, 6.54)	6.36 (6.03, 6.68)	5.58 (5.32, 5.83)	0.73	-0.02 (-0.52, 0.49)
Income-eligible nonparticipants	6.28 (5.91, 6.65)	6.75 (6.40, 7.10)	6.61 (6.31, 6.90)	6.39 (6.04, 6.74)	6.37 (6.06, 6.67)	6.63 (6.39, 6.88)	6.80 (6.41, 7.19)	6.13 (5.86, 6.40)	0.77	-0.15 (-0.60, 0.31)
Higher-Income Individuals	6.06 (5.86, 6.27)	6.39 (6.21, 6.58)	6.27 (6.09, 6.44)	6.20 (6.06, 6.33)	6.15 (6.04, 6.25)	6.19 (6.03, 6.35)	6.23 (6.08, 6.37)	5.96 (5.82, 6.10)	0.09	-0.10 (-0.35, 0.14)
**Whole grains, servings/d**										
SNAP participants	0.41 (0.27, 0.55)	0.42 (0.29, 0.56)	0.46 (0.35, 0.57)	0.46 (0.34, 0.59)	0.47 (0.41, 0.54)	0.64 (0.55, 0.73)	0.78 (0.68, 0.89)	0.66 (0.59, 0.73)	<0.001	0.25 (0.10, 0.40)

© 2018 Zhang FF et al. *JAMA Network Open.*

Income-eligible nonparticipants	0.42 (0.33, 0.51)	0.56 (0.42, 0.70)	0.59 (0.47, 0.71)	0.69 (0.57, 0.80)	0.67 (0.53, 0.81)	0.79 (0.68, 0.90)	0.87 (0.76, 0.98)	0.78 (0.65, 0.92)	<0.001	0.37 (0.20, 0.53)
Higher-Income Individuals	0.61 (0.54, 0.68)	0.77 (0.69, 0.84)	0.70 (0.65, 0.75)	0.81 (0.74, 0.87)	0.80 (0.72, 0.88)	0.96 (0.91, 1.01)	1.08 (0.99, 1.16)	1.00 (0.96, 1.04)	<0.001	0.39 (0.31, 0.47)
									0.62 [b]	0.23 [b]
**Refined grains, servings/d**										
SNAP participants	5.18 (4.67, 5.70)	5.92 (5.28, 6.56)	5.69 (5.41, 5.96)	5.50 (5.15, 5.85)	5.72 (5.42, 6.03)	5.68 (5.48, 5.88)	5.57 (5.27, 5.87)	4.92 (4.67, 5.17)	0.05	-0.27 (-0.83, 0.30)
Income-eligible nonparticipants	5.86 (5.51, 6.21)	6.19 (5.85, 6.52)	6.02 (5.69, 6.35)	5.7 (5.40, 6.01)	5.69 (5.40, 5.99)	5.84 (5.58, 6.1)	5.93 (5.61, 6.26)	5.34 (5.08, 5.61)	0.01	-0.52 (-0.95, -0.08)
Higher-Income Individuals	5.45 (5.26, 5.64)	5.62 (5.45, 5.8)	5.57 (5.41, 5.72)	5.39 (5.25, 5.54)	5.34 (5.25, 5.44)	5.23 (5.07, 5.39)	5.15 (5.01, 5.28)	4.96 (4.83, 5.1)	<0.001	-0.49 (-0.72, -0.26)
									0.65 [b]	0.78 [b]
**Nuts and seeds, servings/d**										
SNAP participants	0.21 (0.11, 0.31)	0.29 (0.01, 0.59)	0.33 (0.24, 0.41)	0.36 (0.26, 0.47)	0.32 (0.21, 0.42)	0.34 (0.28, 0.40)	0.37 (0.28, 0.47)	0.35 (0.24, 0.47)	0.17	0.15 (0.001, 0.29)
Income-eligible nonparticipants	0.42 (0.28, 0.56)	0.43 (0.19, 0.68)	0.52 (0.37, 0.67)	0.44 (0.32, 0.56)	0.42 (0.26, 0.59)	0.42 (0.34, 0.50)	0.42 (0.31, 0.52)	0.46 (0.36, 0.56)	0.92	0.04 (-0.13, 0.21)
Higher-Income Individuals	0.46 (0.4, 0.53)	0.51 (0.43, 0.58)	0.57 (0.50, 0.64)	0.63 (0.57, 0.68)	0.60 (0.53, 0.66)	0.69 (0.63, 0.75)	0.82 (0.74, 0.9)	0.82 (0.75, 0.88)	<0.001	0.36 (0.27, 0.44)
									<0.001 [b]	0.004 [b]
**Legumes, servings/d**										
SNAP participants	0.17 (0.05, 0.29)	0.16 (0.08, 0.23)	0.12 (0.09, 0.15)	0.09 (0.06, 0.12)	0.11 (0.08, 0.14)	0.13 (0.10, 0.17)	0.13 (0.09, 0.16)	0.11 (0.09, 0.12)	0.32	-0.06 (-0.18, 0.06)
Income-eligible nonparticipants	0.13 (0.08, 0.17)	0.14 (0.1, 0.18)	0.12 (0.10, 0.14)	0.13 (0.10, 0.16)	0.15 (0.11, 0.19)	0.14 (0.11, 0.18)	0.16 (0.11, 0.21)	0.14 (0.13, 0.16)	0.18	0.02 (-0.02, 0.06)
Higher-Income Individuals	0.10 (0.08, 0.12)	0.10 (0.08, 0.11)	0.09 (0.07, 0.10)	0.09 (0.08, 0.11)	0.10 (0.09, 0.11)	0.10 (0.09, 0.11)	0.11 (0.10, 0.12)	0.09 (0.09, 0.10)	0.49	-0.01 (-0.03, 0.01)
									0.33 [b]	0.40 [b]
**Total meat, serving/d**										
SNAP participants	1.21 (1.03, 1.38)	1.14 (1.02, 1.27)	1.12 (1.01, 1.22)	1.25 (1.15, 1.35)	1.22 (1.14, 1.3)	1.22 (1.15, 1.28)	1.15 (1.08, 1.22)	1.13 (1.07, 1.2)	0.56	-0.08 (-0.26, 0.11)
Income-eligible nonparticipants	1.02 (0.9, 1.14)	1.11 (1.02, 1.21)	1.03 (0.93, 1.14)	1.21 (1.13, 1.30)	1.08 (1.00, 1.16)	1.20 (1.12, 1.28)	1.18 (1.09, 1.28)	1.08 (0.99, 1.16)	0.11	0.05 (-0.09, 0.20)
Higher-Income Individuals	1.15 (1.09, 1.21)	1.10 (1.07, 1.13)	1.18 (1.14, 1.22)	1.19 (1.15, 1.23)	1.19 (1.16, 1.22)	1.19 (1.13, 1.25)	1.13 (1.08, 1.18)	1.11 (1.08, 1.14)	0.70	-0.04 (-0.11, 0.03)
									0.15 [b]	0.32 [b]
**Processed meats, servings/d**										
SNAP participants	0.29 (0.22, 0.36)	0.23 (0.18, 0.27)	0.27 (0.22, 0.31)	0.31 (0.25, 0.37)	0.30 (0.25, 0.36)	0.32 (0.27, 0.37)	0.29 (0.25, 0.33)	0.31 (0.24, 0.38)	0.18	0.02 (-0.08, 0.12)
Income-eligible nonparticipants	0.20 (0.15, 0.25)	0.26 (0.22, 0.31)	0.26 (0.22, 0.30)	0.25 (0.21, 0.29)	0.26 (0.23, 0.30)	0.28 (0.24, 0.32)	0.26 (0.24, 0.29)	0.26 (0.23, 0.29)	0.09	0.06 (0.00, 0.12)
Higher-Income Individuals	0.29 (0.25, 0.33)	0.30 (0.28, 0.33)	0.30 (0.27, 0.33)	0.30 (0.27, 0.33)	0.29 (0.26, 0.31)	0.30 (0.28, 0.32)	0.29 (0.27, 0.31)	0.26 (0.24, 0.29)	0.11	-0.03 (-0.08, 0.02)
									0.004 [b]	0.03 [b]
**Unprocessed red meats, servings/d**										
SNAP participants	0.53 (0.47, 0.59)	0.53 (0.40, 0.66)	0.46 (0.40, 0.53)	0.55 (0.46, 0.65)	0.52 (0.48, 0.56)	0.49 (0.46, 0.51)	0.45 (0.40, 0.50)	0.40 (0.37, 0.44)	0.001	-0.13 (-0.20, -0.06)
Income-eligible	0.48	0.48	0.42	0.53	0.40	0.46	0.49	0.39	0.19	-0.09

© 2018 Zhang FF et al. *JAMA Network Open.*

nonparticipants	(0.39, 0.56)	(0.40, 0.56)	(0.38, 0.47)	(0.48, 0.58)	(0.35, 0.45)	(0.41, 0.5)	(0.40, 0.58)	(0.36, 0.42)		(-0.17, 0.00)
Higher-Income Individuals	0.48 (0.44, 0.53)	0.44 (0.41, 0.47)	0.48 (0.45, 0.51)	0.47 (0.44, 0.5)	0.46 (0.43, 0.48)	0.45 (0.41, 0.49)	0.44 (0.40, 0.48)	0.42 (0.40, 0.44)	0.03	-0.06 (-0.11, -0.02)
									0.23 [b]	0.51 [b]
**Poultry, servings/d**										
SNAP participants	0.39 (0.25, 0.53)	0.39 (0.28, 0.50)	0.39 (0.32, 0.46)	0.39 (0.33, 0.46)	0.40 (0.33, 0.47)	0.41 (0.36, 0.46)	0.41 (0.35, 0.46)	0.42 (0.38, 0.47)	0.45	0.03 (-0.11, 0.18)
Income-eligible nonparticipants	0.35 (0.27, 0.43)	0.37 (0.32, 0.41)	0.35 (0.28, 0.42)	0.43 (0.36, 0.51)	0.42 (0.36, 0.47)	0.46 (0.40, 0.53)	0.43 (0.39, 0.47)	0.43 (0.36, 0.50)	0.009	0.08 (-0.02, 0.19)
Higher-Income Individuals	0.38 (0.34, 0.42)	0.36 (0.33, 0.39)	0.40 (0.36, 0.44)	0.41 (0.38, 0.44)	0.45 (0.41, 0.49)	0.44 (0.39, 0.49)	0.40 (0.36, 0.44)	0.43 (0.40, 0.46)	0.004	0.05 (0.001, 0.10)
									0.48 [b]	0.83 [b]
**Fish and shellfish, servings/d**										
SNAP participants	0.14 (0.06, 0.23)	0.09 (0.04, 0.14)	0.13 (0.09, 0.16)	0.11 (0.08, 0.15)	0.14 (0.10, 0.17)	0.16 (0.13, 0.19)	0.19 (0.15, 0.23)	0.16 (0.11, 0.22)	0.03	0.02 (-0.08, 0.12)
Income-eligible nonparticipants	0.18 (0.12, 0.24)	0.12 (0.09, 0.14)	0.14 (0.1, 0.17)	0.15 (0.12, 0.17)	0.16 (0.12, 0.19)	0.18 (0.14, 0.22)	0.16 (0.12, 0.2)	0.14 (0.11, 0.16)	0.88	-0.04 (-0.11, 0.02)
Higher-Income Individuals	0.16 (0.13, 0.18)	0.16 (0.13, 0.19)	0.17 (0.14, 0.2)	0.20 (0.16, 0.23)	0.17 (0.15, 0.19)	0.20 (0.17, 0.22)	0.19 (0.16, 0.22)	0.20 (0.16, 0.24)	0.02	0.04 (0.001, 0.09)
									0.28 [b]	0.08 [b]
**Eggs, servings/d**										
SNAP participants	0.59 (0.42, 0.75)	0.37 (0.27, 0.48)	0.48 (0.4, 0.56)	0.45 (0.36, 0.54)	0.55 (0.49, 0.61)	0.53 (0.43, 0.62)	0.49 (0.42, 0.57)	0.47 (0.42, 0.52)	0.87	-0.12 (-0.29, 0.05)
Income-eligible nonparticipants	0.44 (0.39, 0.50)	0.46 (0.39, 0.53)	0.51 (0.42, 0.6)	0.53 (0.46, 0.61)	0.49 (0.45, 0.54)	0.52 (0.44, 0.61)	0.47 (0.39, 0.55)	0.50 (0.43, 0.58)	0.34	0.06 (-0.03, 0.15)
Higher-Income Individuals	0.42 (0.38, 0.46)	0.43 (0.40, 0.46)	0.46 (0.42, 0.51)	0.49 (0.46, 0.52)	0.50 (0.47, 0.54)	0.49 (0.45, 0.52)	0.52 (0.49, 0.55)	0.54 (0.5, 0.57)	<0.001	0.12 (0.06, 0.17)
									0.04 [b]	0.06 [b]
**Total dairy, servings/d**										
SNAP participants	1.27 (1.04, 1.50)	1.14 (0.97, 1.3)	1.39 (1.22, 1.56)	1.42 (1.22, 1.63)	1.24 (1.16, 1.32)	1.51 (1.40, 1.62)	1.53 (1.4, 1.65)	1.39 (1.3, 1.48)	0.008	0.12 (-0.13, 0.36)
Income-eligible nonparticipants	1.31 (1.21, 1.40)	1.33 (1.16, 1.49)	1.34 (1.25, 1.43)	1.46 (1.31, 1.60)	1.45 (1.28, 1.62)	1.50 (1.44, 1.57)	1.50 (1.33, 1.67)	1.39 (1.27, 1.50)	0.03	0.08 (-0.07, 0.23)
Higher-Income Individuals	1.53 (1.44, 1.61)	1.50 (1.44, 1.56)	1.46 (1.37, 1.55)	1.52 (1.45, 1.60)	1.51 (1.42, 1.6)	1.62 (1.57, 1.67)	1.47 (1.42, 1.52)	1.46 (1.4, 1.51)	0.72	-0.07 (-0.17, 0.03)
									0.005 [b]	0.18 [b]
**Milk, servings/d**										
SNAP participants	0.84 (0.69, 0.99)	0.64 (0.50, 0.77)	0.85 (0.70, 1.00)	0.82 (0.65, 0.99)	0.69 (0.62, 0.75)	0.86 (0.74, 0.97)	0.72 (0.59, 0.84)	0.60 (0.54, 0.67)	0.02	-0.24 (-0.40, -0.08)
Income-eligible nonparticipants	0.85 (0.75, 0.95)	0.86 (0.73, 1.00)	0.79 (0.67, 0.90)	0.91 (0.77, 1.05)	0.80 (0.71, 0.9)	0.81 (0.73, 0.89)	0.71 (0.59, 0.83)	0.58 (0.50, 0.67)	<0.001	-0.26 (-0.40, -0.13)
Higher-Income Individuals	0.91 (0.83, 0.99)	0.91 (0.85, 0.96)	0.83 (0.76, 0.90)	0.86 (0.78, 0.94)	0.81 (0.73, 0.88)	0.83 (0.79, 0.87)	0.70 (0.65, 0.74)	0.63 (0.60, 0.67)	<0.001	-0.28 (-0.37, -0.19)
									0.26 [b]	0.89 [b]
**Cheese, servings/d**										
SNAP participants	0.43 (0.32, 0.55)	0.48 (0.36, 0.60)	0.53 (0.44, 0.61)	0.56 (0.45, 0.66)	0.50 (0.45, 0.55)	0.61 (0.55, 0.68)	0.73 (0.66, 0.81)	0.69 (0.63, 0.74)	<0.001	0.25 (0.12, 0.38)
Income-eligible nonparticipants	0.42 (0.33, 0.5)	0.40 (0.34, 0.47)	0.50 (0.43, 0.58)	0.47 (0.41, 0.52)	0.53 (0.45, 0.62)	0.6 0 (0.55, 0.65)	0.69 (0.6, 0.79)	0.68 (0.6, 0.75)	<0.001	0.26 (0.15, 0.37)

© 2018 Zhang FF et al. *JAMA Network Open.*

Higher-Income Individuals	0.57 (0.53, 0.61)	0.54 (0.50, 0.58)	0.59 (0.55, 0.63)	0.59 (0.56, 0.63)	0.62 (0.58, 0.65)	0.68 (0.64, 0.72)	0.67 (0.63, 0.71)	0.69 (0.66, 0.73)	<0.001	0.12 (0.07, 0.18)
									0.0012 [b]	0.06 [b]
**Yogurt, servings/d**										
SNAP participants	0.01 (0, 0.02)	0.01 (0.00, 0.03)	0.03 (0.02, 0.04)	0.02 (0.01, 0.03)	0.02 (0.01, 0.04)	0.03 (0.02, 0.04)	0.04 (0.03, 0.05)	0.04 (0.02, 0.05)	<0.001	0.03 (0.01, 0.05)
Income-eligible nonparticipants	0.02 (0.01, 0.04)	0.03 (0.00, 0.05)	0.02 (0.01, 0.03)	0.05 (0.03, 0.07)	0.04 (0.02, 0.06)	0.03 (0.02, 0.04)	0.05 (0.04, 0.06)	0.07 (0.04, 0.10)	<0.001	0.05 (0.02, 0.08)
Higher-Income Individuals	0.05 (0.03, 0.06)	0.06 (0.05, 0.07)	0.04 (0.03, 0.05)	0.05 (0.04, 0.06)	0.06 (0.05, 0.07)	0.08 (0.07, 0.09)	0.08 (0.07, 0.09)	0.08 (0.07, 0.09)	<0.001	0.03 (0.01, 0.05)
									0.32 [b]	0.43 [b]
**Sugar sweetened beverages, servings/d**										
SNAP participants	2.29 (1.78, 2.81)	2.60 (2.2, 3.0)	2.24 (1.92, 2.55)	2.16 (1.75, 2.56)	2.47 (2.02, 2.92)	2.14 (1.95, 2.32)	1.78 (1.59, 1.97)	1.87 (1.56, 2.17)	0.001	-0.43 (-1.02, 0.17)
Income-eligible nonparticipants	2.09 (1.68, 2.5)	1.97 (1.72, 2.22)	1.94 (1.75, 2.12)	1.79 (1.53, 2.04)	1.68 (1.41, 1.95)	1.54 (1.36, 1.73)	1.48 (1.28, 1.68)	1.16 (0.98, 1.34)	<0.001	-0.93 (-1.37, -0.49)
Higher-Income Individuals	1.59 (1.44, 1.74)	1.48 (1.30, 1.66)	1.44 (1.28, 1.60)	1.27 (1.15, 1.39)	1.23 (1.09, 1.37)	1.05 (0.97, 1.13)	1.05 (0.88, 1.22)	0.80 (0.71, 0.88)	<0.001	-0.80 (-0.97, -0.63)
									0.60 [b]	0.25 [b]
**Added sugars, tsp equivalents/d, servings/d**										
SNAP participants	23.9 (21.0, 26.8)	26.9 (22.9, 30.9)	22.0 (19.9, 24.0)	20.3 (18.3, 22.3)	22.5 (20.1, 24.9)	21.0 (19.9, 22.1)	19.0 (17.6, 20.3)	19.9 (18.4, 21.5)	<0.001	-4.0 (-7.3, -0.7)
Income-eligible nonparticipants	23.5 (21.0, 25.9)	21.1 (19.7, 22.6)	20.1 (18.5, 21.6)	18.93 (17.3, 20.6)	20.2 (18.0, 22.3)	17.4 (16.2, 18.5)	16.9 (15.6, 18.2)	15.8 (14.5, 17.1)	<0.001	-7.6 (-10.4, -4.9)
Higher-Income Individuals	19.6 (18.3, 20.8)	18.4 (17.3, 19.6)	16.6 (15.9, 17.3)	15.9 (15.2, 16.6)	16.0 (15.1, 16.9)	15.2 (14.6, 15.8)	15.2 (14.4, 15.9)	13.6 (13.1, 14.1)	<0.001	-6.0 (-7.3, -4.7)
									0.24 [b]	0.05 [b]
**Total fat, % of energy**										
SNAP participants	32.2 (30.9, 33.5)	31.7 (30.4, 33.1)	33.0 (31.9, 34.14)	33.1 (32.2, 34.0)	32.7 (31.8, 33.6)	31.9 (31.3, 32.5)	32.6 (32.0, 33.2)	33.1 (32.6, 33.6)	0.29	0.9 (-0.5, 2.3)
Income-eligible nonparticipants	31.5 (30.2, 32.8)	32.9 (31.9, 33.9)	33.3 (32.2, 34.4)	32.3 (31.7, 32.9)	31.8 (30.8, 32.9)	31.4 (30.9, 31.9)	32.5 (31.7, 33.2)	33.4 (32.9, 33.9)	0.39	1.9 (0.4, 3.3)
Higher-Income Individuals	33.03 (32.2, 33.9)	33.5 (32.9, 34.0)	34.1 (33.4, 34.8)	34.0 (33.5, 34.4)	34.0 (33.6, 34.5)	33.5 (33.0, 33.9)	33.7 (33.1, 34.3)	34.9 (34.5, 35.2)	0.004	1.8 (0.9, 2.7)
									0.53 [b]	0.43 [b]
**Saturated fat, % of energy**										
SNAP participants	11.1 (10.5, 11.6)	10.3 (9.9, 10.9)	11.1 (10.5, 11.7)	11.4 (10.9, 12.0)	10.8 (10.4, 11.1)	10.7 (10.5, 10.8)	10.8 (10.5, 11.1)	10.9 (10.6, 11.16)	0.61	-0.2 (-0.8, 0.4)
Income-eligible nonparticipants	10.3 (9.8, 10.7)	10.4 (10.0, 10.7)	10.9 (10.5, 11.3)	10.8 (10.5, 11.0)	10.5 (9.9, 11.0)	10.2 (9.9, 10.5)	10.6 (10.2, 11.1)	10.9 (10.7, 11.2)	0.14	0.6 (0.1, 1.2)
Higher-Income Individuals	11.0 (10.7, 11.4)	10.6 (10.4, 10.8)	11.2 (11.0, 11.5)	11.3 (11.2, 11.5)	11.2 (11.0, 11.4)	10.8 (10.6, 11.0)	10.7 (10.5, 11.0)	11.1 (11.0, 11.3)	0.96	0.1 (-0.3, 0.5)
									0.25 [b]	0.04 [b]
**Monounsaturated fat, % of energy**										
SNAP participants	18.5 (10.1, 27.0)	16.3 (13.2, 19.4)	14.2 (13.4, 15.0)	14.7 (13.5, 16.0)	16.1 (14.5, 17.6)	13.1 (12.7, 13.5)	13.4 (12.5, 14.2)	13.4 (12.8, 14.1)	0.05	-5.1 (-13.5, 3.3)
Income-eligible nonparticipants	15.7 (14.2, 17.2)	16.2 (14.9, 17.6)	14.8 (13.9, 15.6)	14.0 (13.0, 15.0)	15.0 (13.9, 16.2)	13.6 (12.9, 14.3)	12.8 (11.8, 13.8)	13.6 (13.0, 14.2)	<0.001	-2.1 (-3.7, -0.6)
Higher-Income Individuals	13.9	13.2	13.4	13.6	13.8	13.1	13.0	13.6	0.22	-0.3

© 2018 Zhang FF et al. *JAMA Network Open.*

	(13.2, 14.6)	(12.8, 13.7)	(12.9, 13.9)	(13.1, 14.1)	(13.4, 14.2)	(12.7, 13.5)	(12.5, 13.5)	(13.2, 13.9)		(-1.1, 0.5)
									<0.001 [b]	0.04 [b]
**Polyunsaturated fat, % of energy**										
SNAP participants	6.1 (5.8, 6.4)	6.3 (5.9, 6.6)	6.7 (6.4, 7.0)	6.6 (6.1, 7.1)	6.8 (6.5, 7.1)	6.8 (6.5, 7.1)	7.6 (7.4, 7.8)	7.6 (7.3, 7.9)	<0.001	1.5 (1.1 ,1.9)
Income-eligible nonparticipants	6.9 (6.5, 7.3)	6.9 (6.5, 7.2)	7.1 (6.6, 7.6)	6.8 (6.6, 7.0)	6.9 (6.7, 7.1)	7.0 (6.8, 7.2)	7.7 (7.4, 7.9)	7.7 (7.4, 7.9)	<0.001	0.7 (0.2, 1.2)
Higher-Income Individuals	7.0 (6.8, 7.2)	6.9 (6.8, 7.0)	7.3 (7.1, 7.5)	7.3 (7.1, 7.5)	7.4 (7.3, 7.5)	7.6 (7.4, 7.7)	8.2 (8.0, 8.3)	8.2 (8.1, 8.4)	<0.001	1.3 (1.0, 1.5)
									0.003 [b]	0.06 [b]
**Seafood omega-3 fat, mg/d**										
SNAP participants	123 (66, 179)	88 (52, 125)	96 (73, 119)	119 (73, 166)	107 (81, 134)	103 (85, 120)	114 (94, 135)	103 (74, 132)	0.98	-20 (-82, 43)
Income-eligible nonparticipants	132 (96, 168)	118 (84, 151)	109 (84, 134)	108 (81, 134)	142 (108, 175)	125 (98, 151)	114 (93, 136)	79 (64, 95)	0.09	-53 (-92, -14)
Higher-Income Individuals	123 (107, 139)	131 (114, 148)	144 (121, 168)	168 (141, 195)	162 (146, 178)	131 (113, 149)	117 (99, 135)	122 (108, 135)	0.21	-1 (-22 ,19)
									0.56 [b]	0.06 [b]
**Plant omega-3 fat, mg/d**										
SNAP participants	117 (107, 126)	125 (117, 132)	127 (122, 131)	125 (117, 132)	129 (124, 134)	132 (126, 138)	155 (149, 160)	146 (141, 151)	<0.001	29 (18, 39)
Income-eligible nonparticipants	125 (116, 133)	134 (125, 143)	143 (131, 155)	133 (128, 138)	137 (129, 144)	141 (136, 146)	161 (155, 166)	149 (143, 154)	<0.001	24 (14, 34)
Higher-Income Individuals	138 (133, 143)	137 (135, 139)	145 (140, 149)	144 (140, 149)	144 (141, 148)	153 (150, 156)	170 (165, 174)	166 (161, 170)	<0.001	27 (21, 34)
									0.32 [b]	0.80 [b]
**Polyunsaturated: saturated fat ratio**										
SNAP participants	0.63 (0.58, 0.67)	0.70 (0.66, 0.74)	0.66 (0.63, 0.69)	0.65 (0.59, 0.71)	0.69 (0.66, 0.72)	0.69 (0.66, 0.73)	0.77 (0.74, 0.80)	0.77 (0.73, 0.81)	<0.001	0.14 (0.08, 0.20)
Income-eligible nonparticipants	0.75 (0.71, 0.78)	0.76 (0.71, 0.81)	0.73 (0.7, 0.76)	0.69 (0.66, 0.73)	0.75 (0.68, 0.83)	0.75 (0.73, 0.78)	0.79 (0.75, 0.82)	0.76 (0.73, 0.79)	0.15	0.01 (-0.03, 0.06)
Higher-Income Individuals	0.72 (0.69, 0.75)	0.73 (0.71, 0.76)	0.70 (0.69, 0.72)	0.70 (0.69, 0.72)	0.72 (0.70, 0.74)	0.77 (0.75, 0.79)	0.82 (0.8, 0.84)	0.80 (0.79, 0.82)	<0.001	0.09 (0.05, 0.12)
									<0.001 [b]	0.01 [b]
**Protein, % of energy**										
SNAP participants	15.1 (14.4, 15.9)	14.5 (13.8, 15.2)	14.8 (14.2, 15.4)	15.6 (14.8, 16.4)	15.4 (14.9, 15.8)	15.4 (15.0, 15.7)	15.5 (15.2, 15.9)	15.5 (15.0, 16.1)	0.02	0.4 (-0.5, 1.3)
Income-eligible nonparticipants	14.8 (14.3, 15.3)	15.2 (14.6, 15.7)	14.9 (14.4, 15.4)	16.0 (15.3, 16.8)	15.6 (15.2, 16.0)	16.1 (15.6, 16.5)	16.0 (15.5, 16.5)	16.2 (15.5, 16.8)	<0.001	1.4 (0.6, 2.2)
Higher-Income Individuals	15.5 (15.3, 15.7)	15.3 (15.0, 15.6)	15.9 (15.6, 16.1)	16.2 (15.9, 16.4)	16.1 (15.9, 16.3)	16.2 (15.9, 16.6)	15.9 (15.6, 16.2)	16.6 (16.3, 16.8)	<0.001	1.1 (0.7, 1.4)
									0.29 [b]	0.30 [b]
**Carbohydrate, % of energy**										
SNAP participants	51.4 (49.9, 52.9)	53.8 (52.4, 55.3)	50.9 (49.8, 52.1)	49.3 (47.2, 51.5)	51.0 (49.8, 52.3)	51.5 (50.7, 52.3)	50.4 (49.2, 51.5)	50.1 (49.3, 50.9)	0.008	-1.3 (-3.0, 0.5)
Income-eligible nonparticipants	53.3 (51.9, 54.6)	51.7 (50.6, 52.8)	50.9 (50.1, 51.8)	50.8 (49.9, 51.7)	51.4 (50.4, 52.4)	50.9 (50.4, 51.3)	50.9 (50.2, 51.6)	49.3 (48.4, 50.1)	<0.001	-4.0 (-5.6, -2.4)
Higher-Income Individuals	50.1 (49.3, 50.9)	50.3 (49.4, 51.2)	48.4 (47.7, 49.1)	48.3 (47.8, 48.9)	48.5 (48.0, 49.1)	48.6 (48.1, 49.1)	48.5 (47.7, 49.2)	46.8 (46.3, 47.2)	<0.001	-3.3 (-4.2, -2.4)

© 2018 Zhang FF et al. *JAMA Network Open.*

									0.47 [b]	0.04 [b]
**Cholesterol, mg/d**										
SNAP participants	316<br>(283, 348)	254<br>(226, 281)	281<br>(266, 296)	284<br>(255, 313)	303<br>(288, 318)	276<br>(260, 291)	279<br>(261, 296)	267<br>(253, 281)	0.08	-49<br>(-84, -14)
Income-eligible nonparticipants	271<br>(250, 292)	280<br>(261, 299)	278<br>(256, 299)	292<br>(271, 313)	278<br>(265, 291)	287<br>(264, 310)	271<br>(253, 290)	269<br>(249, 289)	0.72	-3<br>(-31, 26)
Higher-Income Individuals	267<br>(257, 278)	257<br>(249, 266)	277<br>(266, 288)	277<br>(268, 286)	281<br>(271, 291)	266<br>(257, 274)	273<br>(261, 284)	278<br>(271, 284)	0.06	10<br>(-2, 22)
									0.05 [b]	0.01 [b]
**Fiber, g/d**										
SNAP participants	13.7<br>(10.7, 16.7)	13.2<br>(12.3, 14.1)	12.7<br>(11.9, 13.5)	13.1<br>(12.0, 14.2)	13.7<br>(12.7, 14.6)	14.6<br>(13.9, 15.4)	15.6<br>(14.6, 16.6)	13.5<br>(12.6, 14.4)	0.10	-0.2<br>(-3.3, 2.9)
Income-eligible nonparticipants	14.7<br>(13.6, 15.7)	14.8<br>(13.7, 15.9)	15.0<br>(14.1, 16.0)	15.8<br>(15.0, 16.7)	15.7<br>(14.4, 17.0)	16.5<br>(16.1, 17.0)	17.6<br>(16.5, 18.7)	15.7<br>(14.9, 16.6)	<0.001	1.1<br>(-0.2, 2.4)
Higher-Income Individuals	15.35<br>(14.6, 16.1)	15.7<br>(15.2, 16.2)	15.4<br>(14.9, 15.9)	16.0<br>(15.5, 16.5)	16.6<br>(16.0, 17.2)	17.7<br>(17.3, 18.1)	18.1<br>(17.6, 18.7)	17.0<br>(16.7, 17.4)	<0.001	1.7<br>(0.9, 2.5)
									0.24 [b]	0.29 [b]
**Sodium, mg/d**										
SNAP participants	3383<br>(3178, 3587)	3297<br>(3097, 3498)	3280<br>(3164, 3395)	3344<br>(3164, 3525)	3433<br>(3361, 3504)	3361<br>(3304, 3418)	3381<br>(3298, 3464)	3100<br>(3029, 3172)	0.06	-282<br>(-497, -67)
Income-eligible nonparticipants	3285<br>(3145, 3425)	3401<br>(3272, 3530)	3433<br>(3274, 3592)	3428<br>(3278, 3578)	3411<br>(3291, 3531)	3493<br>(3381, 3606)	3533<br>(3422, 3643)	3239<br>(3159, 3319)	0.51	-46<br>(-206, 114)
Higher-Income Individuals	3459<br>(3389, 3529)	3354<br>(3310, 3398)	3484<br>(3432, 3536)	3601<br>(3530, 3672)	3564<br>(3511, 3617)	3509<br>(3461, 3557)	3438<br>(3400, 3476)	3330<br>(3278, 3381)	0.20	-129<br>(-215, -43)
									0.20 [b]	0.32 [b]
**Potassium, mg/d**										
SNAP participants	2392<br>(2205, 2579)	2236<br>(2118, 2353)	2359<br>(2268, 2450)	2515<br>(2400, 2631)	2352<br>(2269, 2436)	2454<br>(2393, 2515)	2452<br>(2382, 2522)	2199<br>(2114, 2284)	0.39	-193<br>(-396 ,11)
Income-eligible nonparticipants	2533<br>(2414, 2652)	2446<br>(2347, 2546)	2501<br>(2425, 2577)	2635<br>(2533, 2738)	2519<br>(2425, 2612)	2613<br>(2543, 2683)	2625<br>(2538, 2711)	2373<br>(2286, 2460)	0.97	-161<br>(-307, -15)
Higher-Income Individuals	2724<br>(2650, 2799)	2675<br>(2621, 2728)	2659<br>(2620, 2699)	2709<br>(2647, 2772)	2687<br>(2631, 2743)	2803<br>(2765, 2840)	2779<br>(2720, 2838)	2597<br>(2554, 2639)	0.93	-128<br>(-213, -43)
									0.66 [b]	0.73 [b]
**Magnesium, mg/d**										
SNAP participants	234<br>(215, 252)	219<br>(209, 230)	231<br>(219, 243)	269<br>(252, 286)	253<br>(241, 264)	270<br>(262, 277)	274<br>(265, 282)	248<br>(234, 261)	<0.001	14<br>(-9, 37)
Income-eligible nonparticipants	253<br>(241, 265)	250<br>(238, 262)	255<br>(243, 267)	282<br>(272, 291)	287<br>(268, 306)	292<br>(284, 300)	289<br>(277, 297)	277<br>(265, 288)	<0.001	24<br>(8, 40)
Higher-Income Individuals	273<br>(264, 282)	271<br>(263, 279)	271<br>(265, 277)	299<br>(293, 304)	297<br>(290, 305)	310<br>(306, 314))	310<br>(302, 317)	296<br>(292, 301)	<0.001	23<br>(13, 33)
									0.82 [b]	0.71 [b]
**Calcium, mg/d**										
SNAP participants	698<br>(633, 763)	671<br>(627, 715)	761<br>(709, 814)	861<br>(781, 941)	794<br>(765, 824)	927<br>(905, 950)	944<br>(895, 992)	839<br>(808, 871)	<0.001	141<br>(69, 213)
Income-eligible nonparticipants	717<br>(685, 748)	744<br>(689, 798)	776<br>(740, 812)	872<br>(827, 917)	903<br>(841, 966)	930<br>(906, 954)	968<br>(900, 1035)	881<br>(840, 921)	<0.001	164<br>(113, 215)
Higher-Income Individuals	806<br>(775, 838)	825<br>(805, 846)	841<br>(812, 870)	921<br>(898, 943)	930<br>(905, 954)	1001<br>(982, 1019)	936<br>(913, 960)	911<br>(895, 927)	<0.001	105<br>(70 ,140)
									0.005 [b]	0.21 [b]

© 2018 Zhang FF et al. *JAMA Network Open.*

Abbreviations: AHA, American Heart Association; SNAP: Supplemental Nutrition Assistance Program

[a] Means were adjusted for energy to 2000 kcal/d using the residual method except for the means of fat, protein and carbohydrate, which were adjusted as percentage (%) of total energy

[b] Survey-weighted P for interaction that assessed whether the trend in mean take or change in mean intake differed by SNAP participation status.

© 2018 Zhang FF et al. *JAMA Network Open.*

eTable 3. Adjusted Trends in the American Heart Association (AHA) 2020 Diet Score by SNAP Participation Status, 2003-2014[a]

Diet Score	Survey-Weighted Mean Score (95% CI) [a]						P for Trend	Mean Change From 2003-2004 to 2013-2014 (95% CI)
	2003-2004 (n=4066)	2005-2006 (n=4030)	2007-2008 (n=4654)	2009-2010 (n=4996)	2011-2012 (n=4288)	2013-2014 (n=4437)		
**Total AHA diet score (unadjusted)**								
SNAP participants	31.5 (29.2, 33.8)	30.8 (28.6, 32.9)	31.1 (29.5, 32.8)	32.8 (31.2, 34.5)	33.8 (32.7, 34.8)	32.1 (30.6, 33.6)	0.11	0.57 (-2.18, 0.33)
Income-eligible nonparticipants	34.2 (32.6, 35.9)	34.7 (33.1, 36.3)	34.9 (32.7, 37.1)	36.3 (34.8, 37.9)	36.5 (35.2, 37.7)	36.8 (35.3, 38.3)	0.004	2.56 (0.36, 4.76)
Higher-income individuals	35.8 (34.6, 36.9)	36.4 (35.3, 37.4)	36.7 (35.5, 37.8)	38.7 (37.9, 39.4)	40.0 (38.5, 41.5)	39.6 (38.7, 40.5)	<0.001	3.84 (2.39, 5.29)
							0.02 [b]	0.04 [c]
**Total AHA diet score (adjusted for differences in age, sex, and race/ethnicity over time)**								
SNAP participants	32.5 (30.4, 34.6)	31.9 (29.8, 34.1)	31.9 (30.4, 33.3)	33.3 (31.8, 34.8)	34.4 (33.4, 35.5)	32.5 (30.9, 34.1)	0.21	0.01 (-2.17, 2.20)
Income-eligible nonparticipants	34.4 (32.8, 35.9)	34.4 (32.9, 36.0)	34.6 (32.2, 37.1)	36.3 (34.7, 38.0)	36.4 (35.0, 37.7)	36.7 (35.2, 38.3)	0.008	2.16 (0.01, 4.30)
Higher-income individuals	35.9 (34.8, 37.1)	36.5 (35.4, 37.5)	36.7 (35.5, 37.8)	38.5 (37.8, 39.2)	39.7 (38.1, 41.2)	39.2 (38.3, 40.2)	<0.001	3.33 (1.86, 4.80)
							0.01 [b]	0.02 [c]
**Total AHA diet score (adjusted for differences in age, sex, race/ethnicity, and education over time)**								
SNAP participants	34.2 (32.2,36.1)	33.4(31.2,35.5)	33.7(32.3,35.1)	35(33.6,36.4)	35.8(34.7,36.8)	33.9(32.4,35.4)	0.35	-0.25 (-2.36, 1.89)
Income-eligible nonparticipants	35.8 (34.3,37.3)	35.6(34.1,37.1)	35.9(33.8,38)	37.1(35.7,38.6)	37.3(36.2,38.4)	37.4(35.9,39)	0.04	1.51 (-0.54, 3.57)
Higher-income individuals	35.9 (34.9,36.8)	36.3(35.3,37.3)	36.5(35.5,37.4)	38.1(37.4,38.7)	38.8(37.3,40.2)	38.3(37.4,39.2)	<0.001	2.48 (1.23, 3.72)
							0.06 [b]	0.048 [c]
**Total AHA diet score (adjusted for differences in age, sex, race/ethnicity, and income over time)**								
SNAP participants	34.8 (32.8, 36.9)	33.9 (31.7,36.1)	34.2(32.6,35.9)	35.7 (34.3,37.0)	36.6(35.4,37.7)	34.5(33,36)	0.27	-0.16 (-2.33, 2.01)
Income-eligible nonparticipants	37.0 (35.4, 38.6)	37.1 (35.5,38.7)	37.3(34.9,39.6)	39.0 (37.3,40.7)	39.1(37.6,40.6)	39.3(37.6,40.9)	<0.01	2.18 (0.04, 4.32)
Higher-income individuals	35.1 (34.0, 36.2)	35.6 (34.5,36.7)	35.8(34.5,37.1)	37.6(36.9,38.3)	38.9(37.3,40.5)	38.3(37.4,39.2)	<0.001	3.17 (1.78, 4.56)
							0.04 [b]	0.01 [c]

Abbreviations: AHA, American Heart Association; SNAP: Supplemental Nutrition Assistance Program

[a]. The total AHA diet score is the sum of the scores for 8 primary dietary components (fruits/vegetables, whole grains, fish/shellfish, sugar-sweetened beverages, and sodium, nuts/seeds/legumes, processed meat, and saturated fat).

[b]. P for interaction assessing potential heterogeneous trends in total AHA diet score by SNAP participation status (SNAP participants, income-eligible nonparticipants, and higher-income individuals)

[c]. P for interaction assessing potential heterogeneous changes in total AHA diet score from 2003-2004 to 2013-2014 by SNAP participation status (SNAP participants, income-eligible nonparticipants, and higher-income individuals)

© 2018 Zhang FF et al. *JAMA Network Open.*

eTable 4. Adjusted Trends in AHA Diet Score Among U.S. Adults by SNAP Participation Status, 2003-2014[a]

Diet Score	Survey-Weighted Mean Score (95% CI)						P for Trend	Mean Change From 2003-2004 To 2013-2014 (95% CI)
	2003-2004 (n=4066)	2005-2006 (n=4030)	2007-2008 (n=4654)	2009-2010 (n=4996)	2011-2012 (n=4288)	2013-2014 (n=4437)		
**Total AHA Diet Score**								
SNAP participants	32.5 (30.4, 34.6)	31.9 (29.8, 34.1)	31.9 (30.4, 33.3)	33.3 (31.8, 34.8)	34.4 (33.4, 35.5)	32.5 (30.9, 34.1)	0.21	0.0 (-2.2, 2.2)
Income-eligible nonparticipants	34.4 (32.8, 35.9)	34.4 (32.9, 36.0)	34.6 (32.2, 37.1)	36.3 (34.7, 38.0)	36.4 (35.0, 37.7)	36.7 (35.2, 38.3)	0.008	2.2 (0.0, 4.3)
Higher-Income Individuals	35.9 (34.8, 37.1)	36.5 (35.4, 37.5)	36.7 (35.5, 37.8)	38.5 (37.8, 39.2)	39.7 (38.1, 41.2)	39.2 (38.3, 40.2)	<0.001	3.3 (1.9, 4.8)
							0.01 [b]	0.02 [b]
**Fruits and vegetables**								
SNAP participants	4.04 (3.71, 4.38)	4.13 (3.69, 4.58)	4.00 (3.62, 4.37)	4.07 (3.79, 4.35)	4.20 (3.94, 4.46)	3.77 (3.46, 4.07)	0.45	-0.25 (-0.67, 0.16)
Income-eligible nonparticipants	4.67 (4.20, 5.14)	4.79 (4.54, 5.04)	4.45 (4.10, 4.80)	4.75 (4.53, 4.98)	4.86 (4.50, 5.22)	4.61 (4.21, 5.01)	0.85	-0.11 (-0.69, 0.48)
Higher-Income Individuals	5.22 (4.96, 5.48)	5.17 (4.96, 5.39)	5.16 (4.90, 5.41)	5.39 (5.25, 5.53)	5.36 (5.07, 5.65)	5.10 (4.93, 5.27)	0.81	-0.11 (-0.43, 0.2)
							0.63 [b]	0.80 [b]
**Whole grains**								
SNAP participants	1.71 (1.22, 2.19)	1.79 (1.47, 2.12)	1.79 (1.59, 1.99)	2.19 (1.89, 2.48)	2.61 (2.25, 2.96)	2.40 (2.18, 2.62)	<0.001	0.68 (0.20, 1.17)
Income-eligible nonparticipants	2.01 (1.59, 2.42)	2.20 (1.83, 2.56)	2.07 (1.74, 2.41)	2.64 (2.33, 2.96)	2.79 (2.47, 3.11)	2.67 (2.26, 3.08)	0.002	0.66 (0.09, 1.24)
Higher-Income Individuals	2.15 (1.96, 2.34)	2.52 (2.36, 2.67)	2.54 (2.30, 2.78)	2.95 (2.77, 3.13)	3.23 (2.98, 3.47)	3.14 (3.02, 3.25)	<0.001	0.99 (0.77, 1.22)
							0.51 [b]	0.30 [b]
**Fish and shellfish**								
SNAP participants	2.16 (1.64, 2.67)	1.93 (1.36, 2.51)	1.88 (1.54, 2.22)	2.34 (1.75, 2.93)	2.06 (1.67, 2.46)	1.90 (1.38, 2.43)	0.90	-0.20 (-0.89, 0.49)
Income-eligible nonparticipants	2.30 (1.80, 2.79)	2.18 (1.73, 2.62)	2.06 (1.54, 2.58)	2.60 (2.02, 3.18)	2.09 (1.63, 2.56)	2.14 (1.76, 2.52)	0.76	-0.14 (-0.72, 0.44)
Higher-Income Individuals	2.70 (2.37, 3.03)	2.79 (2.54, 3.05)	2.62 (2.33, 2.92)	2.83 (2.54, 3.13)	2.64 (2.26, 3.03)	2.66 (2.32, 3.00)	0.73	-0.04 (-0.51, 0.42)
							0.99 [b]	0.82 [b]
**Sugar-sweetened beverages**								
SNAP participants	4.44 (3.87, 5.00)	5.26 (4.63, 5.89)	4.87 (4.26, 5.48)	5.09 (4.68, 5.50)	5.82 (5.35, 6.29)	5.16 (4.43, 5.89)	0.04	0.68 (-0.13, 1.48)
Income-eligible nonparticipants	4.84 (4.29, 5.40)	5.53 (4.89, 6.17)	5.42 (4.84, 6.00)	5.94 (5.37, 6.50)	6.17 (5.68, 6.66)	6.85 (6.21, 7.48)	<0.001	1.97 (1.17, 2.77)
Higher-Income Individuals	5.98 (5.59, 6.36)	6.53 (6.23, 6.83)	6.49 (6.22, 6.76)	7.00 (6.78, 7.22)	6.96 (6.56, 7.36)	7.25 (6.92, 7.57)	<0.001	1.29 (0.80, 1.78)
							0.07 [b]	0.07 [b]
**Sodium**								
SNAP participants	4.26 (3.91, 4.61)	4.04 (3.52, 4.55)	3.79 (3.60, 3.99)	3.86 (3.64, 4.08)	3.87 (3.60, 4.14)	3.92 (3.58, 4.26)	0.23	-0.34 (-0.81, 0.14)
Income-eligible nonparticipants	3.87 (3.40, 4.35)	3.80 (3.43, 4.17)	3.77 (3.54, 4.00)	3.67 (3.42, 3.93)	3.55 (3.36, 3.75)	3.72 (3.39, 4.05)	0.35	-0.15 (-0.71, 0.41)
Higher-Income Individuals	3.66 (3.49, 3.84)	3.39 (3.25, 3.54)	3.42 (3.31, 3.52)	3.57 (3.41, 3.74)	3.83 (3.74, 3.93)	3.76 (3.62, 3.91)	0.003	0.09 (-0.13, 0.31)
							0.007 [b]	0.15 [b]

© 2018 Zhang FF et al. *JAMA Network Open.*

**Nuts, seeds and legumes**								
SNAP participants	4.02 (3.67, 4.37)	4.02 (3.23, 4.80)	3.73 (3.08, 4.38)	4.08 (3.68, 4.49)	4.14 (3.54, 4.74)	4.16 (3.75, 4.57)	0.38	0.16 (-0.41, 0.72)
Income-eligible nonparticipants	4.58 (4.08, 5.08)	3.97 (3.53, 4.41)	4.46 (3.87, 5.04)	4.42 (3.90, 4.94)	4.62 (3.99, 5.24)	4.77 (4.23, 5.31)	0.34	0.13 (-0.64, 0.91)
Higher-Income Individuals	4.43 (4.19, 4.66)	4.65 (4.33, 4.97)	4.72 (4.35, 5.09)	4.78 (4.53, 5.03)	5.47 (5.16, 5.77)	5.39 (5.10, 5.68)	<0.001	0.97 (0.61, 1.34)
							0.03 [b]	0.02 [b]
**Processed meat**								
SNAP participants	7.02 (6.68, 7.36)	6.12 (5.40, 6.85)	6.45 (6.00, 6.90)	6.35 (5.90, 6.80)	6.37 (5.85, 6.88)	6.11 (5.65, 6.57)	0.03	-0.90 (-1.45, -0.36)
Income-eligible nonparticipants	7.00 (6.52, 7.49)	6.77 (6.41, 7.12)	6.70 (6.13, 7.27)	6.69 (6.31, 7.08)	6.73 (6.43, 7.04)	6.67 (6.21, 7.13)	0.28	-0.36 (-1.01, 0.29)
Higher-Income Individuals	6.74 (6.56, 6.92)	6.49 (6.15, 6.83)	6.69 (6.47, 6.92)	6.65 (6.39, 6.92)	6.72 (6.40, 7.04)	6.86 (6.59, 7.14)	0.25	0.12 (-0.21, 0.45)
							0.01 [b]	0.001 [b]
**Saturated fat**								
SNAP participants	4.86 (4.30, 5.43)	4.61 (4.16, 5.07)	5.35 (4.83, 5.87)	5.35 (5.10, 5.59)	5.35 (5.03, 5.67)	5.09 (4.72, 5.45)	0.23	0.19 (-0.45, 0.84)
Income-eligible nonparticipants	5.11 (4.70, 5.52)	5.21 (4.96, 5.45)	5.69 (5.32, 6.06)	5.61 (5.34, 5.88)	5.56 (5.20, 5.92)	5.28 (5.00, 5.57)	0.29	0.15 (-0.33, 0.63)
Higher-Income Individuals	5.05 (4.80, 5.29)	4.90 (4.72, 5.09)	5.02 (4.81, 5.22)	5.35 (5.18, 5.52)	5.47 (5.21, 5.73)	5.06 (4.87, 5.25)	0.02	0.02 (-0.28, 0.32)
							0.94 [b]	0.72 [b]

Abbreviations: AHA, American Heart Association; SNAP: Supplemental Nutrition Assistance Program

[a] Survey-weighted mean scores were adjusted for age, sex, and race/ethnicity

[b] Survey-weighted P for interaction that assessed whether the trend in mean take or change in mean intake differed by SNAP participation status.

© 2018 Zhang FF et al. *JAMA Network Open.*

eTable 5. Adjusted Trends in Percentages of Having Poor, Intermediate, and Ideal Diet among U.S. Adults by SNAP Participation Status, 2003-2014[a]

Percentage of Having Poor, Intermediate, and Ideal Diet	Survey-Weighted % (95% CI)						P for trend	Change in % from 2003-2004 to 2013-2014
	2003-2004 (n=4066)	2005-2006 (n=4030)	2007-2008 (n=4654)	2009-2010 (n=4996)	2011-2012 (n=4288)	2013-2014 (n=4437)		
**Poor diet**								
SNAP participants	49.9 (47.0, 52.9)	49.6 (46.9, 52.3)	49.3 (46.5, 52.0)	49.1 (46.2, 51.9)	48.7 (45.9, 51.4)	48.3 (45.7, 51.0)	0.39	-1.6 (-1.9, -1.4)
Income-eligible nonparticipants	40.8 (38.0, 43.5)	40.4 (37.6, 43.2)	40.1 (37.5, 42.7)	39.9 (37.3, 42.5)	39.6 (37.0, 42.1)	39.2 (36.7, 41.7)	0.17	-1.5 (-1.8, -1.3)
Higher-Income Individuals	35.2 (33.4, 36.9)	34.8 (33.2, 36.5)	34.5 (32.9, 36.2)	34.3 (32.7, 35.9)	34.0 (32.3, 35.7)	33.7 (32.2, 35.2)	<0.001	-1.5 (-1.7, -1.2)
							0.70[b]	0.53[b]
**Intermediate diet**								
SNAP participants	49.3 (46.4, 52.2)	49.6 (47.0, 52.3)	49.9 (47.2, 52.7)	50.1 (47.3, 52.9)	50.5 (47.8, 53.2)	50.8 (48.1, 53.5)	0.53	1.5 (1.3, 1.7)
Income-eligible nonparticipants	58.0 (55.4, 60.6)	58.4 (55.7, 61.1)	58.7 (56.2, 61.2)	58.9 (56.4, 61.3)	59.2 (56.7, 61.6)	59.5 (57.1, 61.8)	0.29	1.4 (1.2, 1.7)
Higher-Income Individuals	62.9 (61.2, 64.6)	63.2 (61.7, 64.8)	63.5 (62.0, 65.1)	63.7 (62.2, 65.2)	63.9 (62.3, 65.6)	64.2 (62.8, 65.7)	<0.001	1.3 (1.0, 1.5)
							0.80[b]	0.64[b]
**Ideal diet**								
SNAP participants	0.8 (0.4, 1.1)	0.8 (0.4, 1.2)	0.8 (0.4, 1.2)	0.8 (0.4, 1.2)	0.8 (0.4, 1.2)	0.8 (0.4, 1.2)	0.11	0.0 (0.0, 0.1)
Income-eligible nonparticipants	1.2 (0.7, 1.7)	1.2 (0.7, 1.7)	1.2 (0.7, 1.7)	1.2 (0.7, 1.7)	1.3 (0.8, 1.8)	1.3 (0.8, 1.8)	0.15	0.1 (0.1, 0.2)
Higher-Income Individuals	1.9 (1.6, 2.2)	1.9 (1.6, 2.3)	2.0 (1.6, 2.3)	2.0 (1.7, 2.3)	2.0 (1.7, 2.4)	2.1 (1.8, 2.4)	0.01	0.2 (0.2, 0.2)
							0.50[b]	0.44[b]

Abbreviations: AHA, American Heart Association; SNAP: Supplemental Nutrition Assistance Program

[a.] Survey-weighted mean scores were adjusted for age, sex, and race/ethnicity

[b.] Survey-weighted P for interaction that assessed whether the trend in mean take or change in mean intake differed by SNAP participation status.

© 2018 Zhang FF et al. *JAMA Network Open.*

eTable 6. Adjusted Trends in Dietary Intake of Key Food Groups and Nutrients Among U.S. Adults by SNAP Participation Status, 1999-2014[a]

Dietary Intake	Survey-Weighted Mean Intake (95% CI)								P for trend	Mean change from 1999-2000 to 2013-2014 (95% CI)
	1999-2000 (n=4232)	2001-2002 (n=4744)	2003-2004 (n=4386)	2005-2006 (n=4473)	2007-2008 (n=5384)	2009-2010 (n=5710)	2011-2012 (n=4774)	2013-2014 (n=4993)		
**Total fruits, servings/d**										
SNAP participants	0.72 (0.54, 0.90)	0.77 (0.60, 0.94)	0.82 (0.68, 0.96)	0.81 (0.63, 0.99)	0.77 (0.63, 0.90)	0.86 (0.79, 0.94)	0.92 (0.81, 1.03)	0.72 (0.61, 0.82)	0.52	0 (-0.21, 0.21)
Income-eligible nonparticipants	0.98 (0.74, 1.21)	0.83 (0.63, 1.03)	0.88 (0.75, 1.02)	0.96 (0.91, 1.02)	0.86 (0.72, 1.00)	0.94 (0.83, 1.05)	0.99 (0.84, 1.13)	0.83 (0.69, 0.96)	0.81	-0.15 (-0.41, 0.11)
Higher-Income Individuals	1.04 (0.91, 1.17)	1.15 (1.05, 1.26)	1.04 (0.92, 1.15)	1.00 (0.92, 1.08)	1.02 (0.92, 1.12)	1.11 (1.06, 1.16)	1.05 (0.96, 1.14)	0.92 (0.86, 0.98)	0.08	-0.12 (-0.26, 0.02)
									0.20 [b]	0.44 [b]
**Whole fruits, servings/d**										
SNAP participants	0.40 (0.27, 0.54)	0.45 (0.36, 0.54)	0.43 (0.35, 0.52)	0.42 (0.32, 0.52)	0.50 (0.40, 0.61)	0.53 (0.48, 0.59)	0.59 (0.49, 0.69)	0.49 (0.39, 0.59)	0.03	0.07 (-0.1, 0.24)
Income-eligible nonparticipants	0.55 (0.40, 0.70)	0.55 (0.40, 0.70)	0.55 (0.45, 0.65)	0.62 (0.58, 0.66)	0.55 (0.44, 0.66)	0.62 (0.52, 0.72)	0.65 (0.58, 0.72)	0.58 (0.49, 0.68)	0.30	0.03 (-0.15, 0.2)
Higher-Income Individuals	0.63 (0.55, 0.72)	0.69 (0.63, 0.74)	0.65 (0.58, 0.73)	0.66 (0.60, 0.72)	0.73 (0.66, 0.80)	0.81 (0.77, 0.84)	0.78 (0.70, 0.85)	0.70 (0.65, 0.76)	0.001	0.07 (-0.03, 0.17)
									0.70 [b]	0.87 [b]
**100% fruit juices, servings/d**										
SNAP participants	0.33 (0.22, 0.43)	0.42 (0.25, 0.58)	0.39 (0.31, 0.47)	0.63 (0.13, 1.13)	0.31 (0.23, 0.38)	0.35 (0.31, 0.39)	0.37 (0.27, 0.47)	0.25 (0.20, 0.30)	0.05	-0.07 (-0.18, 0.04)
Income-eligible nonparticipants	0.47 (0.32, 0.61)	0.32 (0.25, 0.39)	0.35 (0.29, 0.42)	0.33 (0.3, 0.37)	0.35 (0.28, 0.42)	0.36 (0.3, 0.42)	0.35 (0.22, 0.47)	0.30 (0.23, 0.37)	0.12	-0.17 (-0.33, -0.01)
Higher-Income Individuals	0.43 (0.37, 0.49)	0.44 (0.39, 0.49)	0.39 (0.34, 0.45)	0.36 (0.33, 0.39)	0.30 (0.27, 0.34)	0.33 (0.29, 0.36)	0.27 (0.23, 0.31)	0.22 (0.20, 0.24)	<0.001	-0.21 (-0.27, -0.14)
									0.10 [b]	0.17 [b]
**Total vegetables, servings/d**										
SNAP participants	1.48 (1.22, 1.73)	1.37 (1.22, 1.52)	1.45 (1.31, 1.58)	1.40 (1.25, 1.56)	1.41 (1.25, 1.57)	1.29 (1.23, 1.35)	1.37 (1.29, 1.46)	1.22 (1.11, 1.32)	0.01	-0.26 (-0.53, 0.01)
Income-eligible nonparticipants	1.41 (1.27, 1.56)	1.45 (1.28, 1.62)	1.52 (1.43, 1.61)	1.52 (1.39, 1.65)	1.43 (1.31, 1.55)	1.5 (1.42, 1.58)	1.52 (1.41, 1.63)	1.31 (1.19, 1.42)	0.49	-0.11 (-0.3, 0.07)
Higher-Income Individuals	1.70 (1.61, 1.79)	1.64 (1.56, 1.72)	1.67 (1.63, 1.72)	1.66 (1.59, 1.72)	1.66 (1.60, 1.72)	1.68 (1.61, 1.75)	1.70 (1.61, 1.80)	1.55 (1.49, 1.62)	0.21	-0.15 (-0.26, -0.03)
									0.20 [b]	0.70 [b]
**Dark-green vegetables, servings/d**										
SNAP participants	0.06 (0.04, 0.09)	0.06 (0.04, 0.08)	0.05 (0.03, 0.07)	0.11 (0.07, 0.14)	0.07 (0.06, 0.09)	0.09 (0.08, 0.10)	0.09 (0.06, 0.12)	0.1 (0.06, 0.13)	0.02	0.03 (-0.01, 0.07)
Income-eligible nonparticipants	0.06 (0.04, 0.07)	0.08 (0.05, 0.10)	0.08 (0.06, 0.10)	0.11 (0.08, 0.13)	0.10 (0.06, 0.14)	0.10 (0.06, 0.13)	0.13 (0.08, 0.18)	0.11 (0.08, 0.14)	<0.001	0.06 (0.03, 0.09)
Higher-Income Individuals	0.14 (0.10, 0.18)	0.14 (0.11, 0.16)	0.13 (0.11, 0.14)	0.15 (0.13, 0.17)	0.14 (0.12, 0.16)	0.16 (0.14, 0.17)	0.18 (0.14, 0.21)	0.19 (0.16, 0.21)	0.003	0.04 (0, 0.09)
									0.76 [b]	0.69 [b]
**Tomatoes, servings/d**										
SNAP participants	0.25 (0.19, 0.31)	0.35 (0.28, 0.42)	0.34 (0.28, 0.40)	0.28 (0.24, 0.32)	0.27 (0.24, 0.30)	0.26 (0.23, 0.29)	0.29 (0.26, 0.32)	0.22 (0.20, 0.24)	0.005	-0.03 (-0.09, 0.04)
Income-eligible nonparticipants	0.27 (0.21, 0.33)	0.33 (0.27, 0.40)	0.34 (0.27, 0.41)	0.33 (0.28, 0.37)	0.26 (0.24, 0.29)	0.28 (0.25, 0.32)	0.31 (0.28, 0.33)	0.27 (0.24, 0.30)	0.27	0 (-0.07, 0.07)
Higher-Income Individuals	0.31 (0.28, 0.34)	0.34 (0.31, 0.38)	0.37 (0.33, 0.40)	0.32 (0.30, 0.34)	0.33 (0.31, 0.34)	0.31 (0.27, 0.34)	0.31 (0.29, 0.34)	0.28 (0.26, 0.29)	0.002	-0.04 (-0.07, 0)
									0.39 [b]	0.65 [b]

© 2018 Zhang FF et al. *JAMA Network Open.*

**Other red/orange vegetables, servings/d**										
SNAP participants	0.06 (0.04, 0.09)	0.07 (0.05, 0.09)	0.05 (0.03, 0.07)	0.06 (0.04, 0.08)	0.05 (0.04, 0.07)	0.07 (0.06, 0.08)	0.07 (0.05, 0.10)	0.08 (0.06, 0.10)	0.10	0.02 (-0.02, 0.05)
Income-eligible nonparticipants	0.07 (0.05, 0.09)	0.07 (0.06, 0.09)	0.08 (0.06, 0.10)	0.08 (0.07, 0.09)	0.09 (0.05, 0.13)	0.09 (0.08, 0.10)	0.08 (0.06, 0.11)	0.08 (0.06, 0.10)	0.15	0.01 (-0.01, 0.04)
Higher-Income Individuals	0.09 (0.08, 0.11)	0.09 (0.07, 0.11)	0.08 (0.07, 0.08)	0.09 (0.08, 0.10)	0.09 (0.07, 0.10)	0.11 (0.10, 0.12)	0.12 (0.11, 0.14)	0.11 (0.09, 0.12)	<0.001	0.02 (-0.01, 0.04)
									0.48 [b]	0.97 [b]
**White potatoes, servings/d**										
SNAP participants	0.49 (0.40, 0.58)	0.32 (0.23, 0.40)	0.47 (0.41, 0.52)	0.38 (0.31, 0.44)	0.40 (0.36, 0.45)	0.36 (0.33, 0.38)	0.34 (0.29, 0.39)	0.33 (0.30, 0.36)	0.002	-0.16 (-0.25, -0.06)
Income-eligible nonparticipants	0.38 (0.29, 0.47)	0.35 (0.27, 0.43)	0.42 (0.36, 0.49)	0.36 (0.30, 0.42)	0.37 (0.31, 0.44)	0.37 (0.31, 0.42)	0.37 (0.30, 0.45)	0.32 (0.27, 0.36)	0.32	-0.06 (-0.16, 0.04)
Higher-Income Individuals	0.39 (0.34, 0.43)	0.37 (0.35, 0.39)	0.37 (0.34, 0.41)	0.35 (0.32, 0.39)	0.37 (0.33, 0.40)	0.36 (0.34, 0.38)	0.32 (0.29, 0.34)	0.32 (0.29, 0.35)	0.001	-0.07 (-0.12, -0.01)
									0.12 [b]	0.049 [b]
**Other starchy vegetables, servings/d**										
SNAP participants	0.09 (0.05, 0.13)	0.09 (0.04, 0.15)	0.08 (0.05, 0.11)	0.08 (0.06, 0.11)	0.09 (0.07, 0.10)	0.08 (0.07, 0.10)	0.08 (0.04, 0.12)	0.07 (0.05, 0.09)	0.25	-0.02 (-0.07, 0.02)
Income-eligible nonparticipants	0.10 (0.06, 0.13)	0.10 (0.08, 0.13)	0.07 (0.05, 0.09)	0.08 (0.07, 0.10)	0.09 (0.07, 0.10)	0.09 (0.07, 0.11)	0.08 (0.05, 0.11)	0.06 (0.04, 0.08)	0.07	-0.04 (-0.08, 0)
Higher-Income Individuals	0.10 (0.08, 0.11)	0.10 (0.09, 0.11)	0.08 (0.07, 0.09)	0.09 (0.08, 0.11)	0.09 (0.08, 0.11)	0.09 (0.07, 0.10)	0.08 (0.07, 0.09)	0.07 (0.06, 0.08)	<0.001	-0.03 (-0.04, -0.01)
									0.89 [b]	0.88 [b]
**Vegetables, excluding potatoes/starchy, servings/d**										
SNAP participants	0.89 (0.67, 1.11)	0.96 (0.81, 1.11)	0.90 (0.80, 1.00)	0.94 (0.81, 1.08)	0.92 (0.80, 1.04)	0.85 (0.77, 0.93)	0.95 (0.88, 1.03)	0.82 (0.73, 0.91)	0.32	-0.08 (-0.31, 0.16)
Income-eligible nonparticipants	0.94 (0.81, 1.07)	0.99 (0.84, 1.14)	1.02 (0.91, 1.13)	1.08 (0.97, 1.18)	0.97 (0.85, 1.09)	1.04 (0.97, 1.11)	1.07 (0.95, 1.19)	0.93 (0.83, 1.03)	0.80	-0.02 (-0.17, 0.14)
Higher-Income Individuals	1.22 (1.10, 1.33)	1.17 (1.09, 1.25)	1.21 (1.16, 1.27)	1.21 (1.13, 1.28)	1.20 (1.12, 1.27)	1.23 (1.16, 1.30)	1.31 (1.20, 1.41)	1.16 (1.09, 1.24)	0.57	-0.05 (-0.19, 0.08)
									0.48 [b]	0.90 [b]
**Total grains, servings/d**										
SNAP participants	5.46 (4.97, 5.95)	6.48 (5.82, 7.13)	6.12 (5.85, 6.39)	5.93 (5.63, 6.23)	5.98 (5.75, 6.22)	6.24 (6.03, 6.45)	6.31 (5.97, 6.64)	5.47 (5.15, 5.78)	0.29	-0.03 (-0.59, 0.53)
Income-eligible nonparticipants	6.10 (5.74, 6.47)	6.71 (6.28, 7.13)	6.39 (6.08, 6.70)	6.16 (5.82, 6.49)	6.02 (5.72, 6.31)	6.37 (6.14, 6.61)	6.60 (6.11, 7.09)	5.87 (5.56, 6.18)	0.27	-0.27 (-0.76, 0.21)
Higher-Income Individuals	6.08 (5.86, 6.30)	6.32 (6.18, 6.47)	6.27 (6.09, 6.44)	6.18 (6.06, 6.29)	6.10 (5.98, 6.21)	6.15 (6.01, 6.29)	6.14 (5.98, 6.31)	5.91 (5.74, 6.09)	0.04	-0.15 (-0.43, 0.12)
									0.99 [b]	0.81 [b]
**Whole grains, servings/d**										
SNAP participants	0.48 (0.32, 0.63)	0.50 (0.33, 0.67)	0.54 (0.41, 0.67)	0.56 (0.43, 0.69)	0.54 (0.50, 0.58)	0.70 (0.61, 0.78)	0.83 (0.71, 0.96)	0.72 (0.64, 0.8)	<0.001	0.24 (0.06, 0.42)
Income-eligible nonparticipants	0.48 (0.40, 0.57)	0.62 (0.48, 0.75)	0.59 (0.50, 0.68)	0.69 (0.55, 0.84)	0.64 (0.51, 0.76)	0.84 (0.73, 0.95)	0.88 (0.79, 0.97)	0.79 (0.65, 0.94)	<0.001	0.33 (0.16, 0.49)
Higher-Income Individuals	0.62 (0.55, 0.69)	0.75 (0.68, 0.82)	0.69 (0.65, 0.74)	0.81 (0.74, 0.87)	0.79 (0.70, 0.88)	0.92 (0.86, 0.99)	1.05 (0.96, 1.14)	0.96 (0.91, 1.01)	<0.001	0.34 (0.25, 0.42)
									0.83 [b]	0.62 [b]
**Refined grains, servings/d**										
SNAP participants	4.98 (4.49, 5.47)	5.97 (5.27, 6.68)	5.58 (5.26, 5.9)	5.37 (5.10, 5.64)	5.45 (5.21, 5.68)	5.54 (5.36, 5.72)	5.48 (5.18, 5.78)	4.74 (4.46, 5.03)	0.01	-0.26 (-0.82, 0.29)
Income-eligible nonparticipants	5.62 (5.28, 5.95)	6.09 (5.67, 6.51)	5.80 (5.46, 6.14)	5.46 (5.13, 5.80)	5.38 (5.12, 5.64)	5.54 (5.28, 5.79)	5.72 (5.27, 6.17)	5.08 (4.8, 5.35)	0.003	-0.6 (-1.04, -0.16)

© 2018 Zhang FF et al. *JAMA Network Open.*

Higher-Income Individuals	5.46 (5.26, 5.65)	5.58 (5.41, 5.74)	5.57 (5.41, 5.73)	5.37 (5.23, 5.50)	5.31 (5.21, 5.40)	5.23 (5.08, 5.37)	5.10 (4.96, 5.24)	4.96 (4.80, 5.11)	<0.001	-0.49 (-0.74, -0.24)
									0.94[b]	0.68[b]
**Nuts and seeds, servings/d**										
SNAP participants	0.30 (0.19, 0.41)	0.41 (0.05, 0.78)	0.40 (0.30, 0.51)	0.41 (0.34, 0.48)	0.41 (0.26, 0.56)	0.40 (0.32, 0.47)	0.39 (0.29, 0.5)	0.40 (0.28, 0.51)	0.57	0.11 (-0.05, 0.26)
Income-eligible nonparticipants	0.54 (0.38, 0.69)	0.49 (0.27, 0.7)	0.53 (0.44, 0.63)	0.51 (0.39, 0.63)	0.5 0 (0.30, 0.70)	0.50 (0.42, 0.59)	0.43 (0.28, 0.59)	0.49 (0.41, 0.57)	0.54	-0.02 (-0.19, 0.14)
Higher-Income Individuals	0.46 (0.40, 0.53)	0.50 (0.42, 0.58)	0.56 (0.49, 0.63)	0.61 (0.55, 0.67)	0.60 (0.53, 0.66)	0.70 (0.63, 0.76)	0.83 (0.74, 0.92)	0.80 (0.74, 0.87)	<0.001	0.34 (0.25, 0.43)
									<0.001[b]	0.000[b]
**Legumes, servings/d**										
SNAP participants	0.12 (0.05, 0.18)	0.17 (0.08, 0.26)	0.12 (0.09, 0.14)	0.08 (0.05, 0.11)	0.09 (0.06, 0.11)	0.11 (0.09, 0.14)	0.11 (0.08, 0.15)	0.09 (0.08, 0.10)	0.17	-0.03 (-0.09, 0.04)
Income-eligible nonparticipants	0.11 (0.08, 0.14)	0.11 (0.08, 0.13)	0.10 (0.05, 0.14)	0.11 (0.08, 0.13)	0.12 (0.08, 0.17)	0.10 (0.07, 0.13)	0.14 (0.09, 0.19)	0.11 (0.09, 0.13)	0.51	-0.01 (-0.04, 0.03)
Higher-Income Individuals	0.10 (0.08, 0.12)	0.09 (0.08, 0.11)	0.09 (0.08, 0.10)	0.09 (0.08, 0.11)	0.10 (0.08, 0.11)	0.09 (0.08, 0.1)	0.10 (0.09, 0.12)	0.09 (0.08, 0.10)	0.84	-0.01 (-0.03, 0.01)
									0.31[b]	0.85[b]
**Total meat, serving/d**										
SNAP participants	1.26 (1.05, 1.47)	1.10 (0.99, 1.20)	1.10 (1.00, 1.19)	1.23 (1.12, 1.33)	1.22 (1.12, 1.31)	1.19 (1.13, 1.26)	1.10 (1.03, 1.17)	1.14 (1.07, 1.21)	0.56	-0.11 (-0.34, 0.11)
Income-eligible nonparticipants	1.04 (0.91, 1.17)	1.11 (1.00, 1.21)	1.04 (0.90, 1.18)	1.20 (1.10, 1.31)	1.12 (1.04, 1.20)	1.18 (1.10, 1.26)	1.15 (1.04, 1.25)	1.04 (0.97, 1.11)	0.51	-0.01 (-0.15, 0.12)
Higher-Income Individuals	1.14 (1.08, 1.20)	1.10 (1.07, 1.14)	1.18 (1.14, 1.21)	1.19 (1.15, 1.23)	1.20 (1.17, 1.23)	1.19 (1.14, 1.25)	1.14 (1.08, 1.20)	1.12 (1.08, 1.16)	0.64	-0.02 (-0.09, 0.06)
									0.50[b]	0.59[b]
**Processed meats, servings/d**										
SNAP participants	0.36 (0.26, 0.46)	0.24 (0.19, 0.29)	0.30 (0.26, 0.33)	0.33 (0.26, 0.40)	0.33 (0.28, 0.39)	0.33 (0.29, 0.38)	0.30 (0.25, 0.36)	0.34 (0.26, 0.41)	0.54	-0.02 (-0.15, 0.1)
Income-eligible nonparticipants	0.23 (0.16, 0.29)	0.29 (0.24, 0.33)	0.29 (0.24, 0.33)	0.27 (0.23, 0.32)	0.30 (0.26, 0.33)	0.32 (0.27, 0.36)	0.28 (0.26, 0.31)	0.29 (0.26, 0.32)	0.09	0.07 (0, 0.13)
Higher-Income Individuals	0.29 (0.25, 0.33)	0.30 (0.28, 0.33)	0.30 (0.27, 0.32)	0.31 (0.28, 0.33)	0.29 (0.27, 0.31)	0.30 (0.28, 0.32)	0.30 (0.27, 0.33)	0.27 (0.24, 0.30)	0.53	-0.02 (-0.06, 0.03)
									0.12[b]	0.13[b]
**Unprocessed red meats, servings/d**										
SNAP participants	0.55 (0.46, 0.63)	0.51 (0.43, 0.58)	0.48 (0.41, 0.54)	0.58 (0.48, 0.68)	0.53 (0.48, 0.58)	0.49 (0.46, 0.51)	0.46 (0.41, 0.51)	0.41 (0.37, 0.45)	0.005	-0.12 (-0.21, -0.03)
Income-eligible nonparticipants	0.48 (0.41, 0.55)	0.48 (0.41, 0.56)	0.43 (0.38, 0.49)	0.50 (0.45, 0.56)	0.42 (0.37, 0.47)	0.45 (0.40, 0.50)	0.47 (0.36, 0.57)	0.36 (0.33, 0.40)	0.02	-0.13 (-0.2, -0.05)
Higher-Income Individuals	0.48 (0.43, 0.53)	0.43 (0.39, 0.46)	0.48 (0.44, 0.51)	0.47 (0.44, 0.5)	0.46 (0.42, 0.49)	0.45 (0.41, 0.49)	0.44 (0.39, 0.49)	0.42 (0.40, 0.44)	0.07	-0.06 (-0.11, -0.01)
									0.16[b]	0.37[b]
**Poultry, servings/d**										
SNAP participants	0.36 (0.20, 0.52)	0.35 (0.25, 0.44)	0.33 (0.25, 0.40)	0.32 (0.25, 0.38)	0.35 (0.29, 0.41)	0.37 (0.31, 0.44)	0.34 (0.30, 0.38)	0.39 (0.34, 0.44)	0.46	0.03 (-0.14, 0.2)
Income-eligible nonparticipants	0.33 (0.26, 0.41)	0.34 (0.29, 0.39)	0.31 (0.25, 0.37)	0.42 (0.35, 0.50)	0.40 (0.35, 0.46)	0.41 (0.38, 0.45)	0.39 (0.36, 0.43)	0.38 (0.33, 0.44)	0.01	0.05 (-0.04, 0.14)
Higher-Income Individuals	0.37 (0.33, 0.42)	0.37 (0.34, 0.40)	0.40 (0.37, 0.44)	0.42 (0.39, 0.44)	0.45 (0.42, 0.49)	0.44 (0.40, 0.48)	0.40 (0.36, 0.44)	0.43 (0.40, 0.46)	0.005	0.06 (0, 0.12)
									0.81[b]	0.95[b]
**Fish and shellfish, servings/d**										

© 2018 Zhang FF et al. *JAMA Network Open.*

SNAP participants	0.17 (0.07, 0.26)	0.10 (0.05, 0.15)	0.12 (0.08, 0.16)	0.11 (0.08, 0.15)	0.12 (0.09, 0.16)	0.14 (0.12, 0.17)	0.16 (0.11, 0.21)	0.14 (0.08, 0.2)	0.35	-0.02 (-0.13, 0.09)
Income-eligible nonparticipants	0.18 (0.1, 0.26)	0.11 (0.09, 0.13)	0.12 (0.09, 0.15)	0.13 (0.08, 0.18)	0.15 (0.11, 0.19)	0.17 (0.12, 0.22)	0.16 (0.11, 0.21)	0.13 (0.10, 0.15)	0.99	-0.05 (-0.13, 0.02)
Higher-Income Individuals	0.16 (0.13, 0.18)	0.16 (0.13, 0.19)	0.16 (0.13, 0.18)	0.19 (0.16, 0.22)	0.16 (0.14, 0.18)	0.19 (0.16, 0.22)	0.18 (0.14, 0.21)	0.19 (0.15, 0.23)	0.08	0.03 (-0.01, 0.08)
									0.64 [b]	0.09 [b]
**Eggs, servings/d**										
SNAP participants	0.66 (0.46, 0.85)	0.42 (0.29, 0.54)	0.49 (0.41, 0.57)	0.48 (0.38, 0.57)	0.56 (0.49, 0.62)	0.52 (0.44, 0.61)	0.50 (0.42, 0.57)	0.43 (0.39, 0.48)	0.11	-0.23 (-0.43, -0.03)
Income-eligible nonparticipants	0.44 (0.37, 0.52)	0.47 (0.39, 0.55)	0.51 (0.41, 0.61)	0.50 (0.44, 0.56)	0.50 (0.46, 0.54)	0.51 (0.41, 0.61)	0.45 (0.37, 0.53)	0.50 (0.41, 0.59)	0.77	0.04 (-0.07, 0.16)
Higher-Income Individuals	0.43 (0.39, 0.47)	0.44 (0.41, 0.47)	0.47 (0.42, 0.51)	0.49 (0.46, 0.52)	0.50 (0.47, 0.53)	0.49 (0.45, 0.52)	0.51 (0.47, 0.55)	0.54 (0.49, 0.58)	<0.001	0.1 (0.05, 0.16)
									0.009 [b]	0.03 [b]
**Total dairy, servings/d**										
SNAP participants	1.40 (1.03, 1.76)	1.26 (1.13, 1.39)	1.50 (1.33, 1.67)	1.54 (1.3, 1.77)	1.33 (1.24, 1.42)	1.63 (1.51, 1.76)	1.67 (1.55, 1.78)	1.49 (1.38, 1.59)	0.06	0.08 (-0.29, 0.44)
Income-eligible nonparticipants	1.35 (1.21, 1.49)	1.37 (1.18, 1.55)	1.40 (1.29, 1.50)	1.53 (1.39, 1.68)	1.47 (1.3, 1.65)	1.59 (1.48, 1.69)	1.55 (1.36, 1.74)	1.49 (1.35, 1.62)	0.02	0.14 (-0.05, 0.32)
Higher-Income Individuals	1.52 (1.43, 1.61)	1.50 (1.43, 1.57)	1.45 (1.38, 1.52)	1.53 (1.47, 1.59)	1.52 (1.45, 1.59)	1.63 (1.58, 1.69)	1.49 (1.45, 1.54)	1.48 (1.42, 1.53)	0.64	-0.04 (-0.15, 0.06)
									0.03 [b]	0.24 [b]
**Milk, servings/d**										
SNAP participants	0.98 (0.73, 1.22)	0.77 (0.66, 0.87)	0.96 (0.79, 1.12)	0.93 (0.74, 1.12)	0.76 (0.70, 0.82)	0.95 (0.82, 1.08)	0.81 (0.68, 0.94)	0.67 (0.59, 0.76)	0.005	-0.3 (-0.55, -0.06)
Income-eligible nonparticipants	0.89 (0.74, 1.04)	0.88 (0.72, 1.04)	0.83 (0.70, 0.95)	0.93 (0.79, 1.08)	0.83 (0.73, 0.93)	0.85 (0.73, 0.96)	0.72 (0.61, 0.83)	0.61 (0.51, 0.71)	0.001	-0.28 (-0.46, -0.11)
Higher-Income Individuals	0.92 (0.84, 1.00)	0.92 (0.85, 0.98)	0.82 (0.77, 0.87)	0.86 (0.78, 0.93)	0.80 (0.74, 0.87)	0.82 (0.78, 0.86)	0.70 (0.65, 0.74)	0.63 (0.59, 0.66)	<0.001	-0.29 (-0.37, -0.2)
									0.77 [b]	0.99 [b]
**Cheese, servings/d**										
SNAP participants	0.42 (0.24, 0.59)	0.48 (0.36, 0.59)	0.53 (0.46, 0.59)	0.58 (0.45, 0.7)	0.50 (0.43, 0.57)	0.63 (0.57, 0.7)	0.79 (0.7, 0.89)	0.71 (0.65, 0.77)	<0.001	0.29 (0.11, 0.47)
Income-eligible nonparticipants	0.42 (0.34, 0.51)	0.44 (0.36, 0.52)	0.53 (0.43, 0.63)	0.52 (0.47, 0.57)	0.54 (0.46, 0.62)	0.65 (0.60, 0.69)	0.74 (0.63, 0.85)	0.75 (0.66, 0.83)	<0.001	0.32 (0.2, 0.44)
Higher-Income Individuals	0.56 (0.52, 0.61)	0.54 (0.50, 0.57)	0.59 (0.55, 0.64)	0.60 (0.56, 0.64)	0.63 (0.59, 0.67)	0.70 (0.66, 0.74)	0.70 (0.67, 0.73)	0.72 (0.68, 0.76)	<0.001	0.16 (0.1, 0.22)
									0.001 [b]	0.04 [b]
**Yogurt, servings/d**										
SNAP participants	0.02 (0.00, 0.03)	0.02 (0.01, 0.02)	0.03 (0.02, 0.05)	0.02 (0.01, 0.03)	0.03 (0.01, 0.04)	0.03 (0.02, 0.05)	0.04 (0.03, 0.05)	0.04 (0.02, 0.06)	0.005	0.02 (0, 0.05)
Income-eligible nonparticipants	0.03 (0.01, 0.05)	0.03 (0.01, 0.06)	0.02 (0.01, 0.03)	0.05 (0.03, 0.08)	0.04 (0.01, 0.07)	0.04 (0.03, 0.05)	0.06 (0.04, 0.07)	0.08 (0.05, 0.11)	0.0002	0.05 (0.02, 0.09)
Higher-Income Individuals	0.04 (0.02, 0.06)	0.06 (0.05, 0.07)	0.04 (0.03, 0.05)	0.05 (0.04, 0.06)	0.06 (0.05, 0.07)	0.07 (0.07, 0.08)	0.07 (0.06, 0.09)	0.08 (0.07, 0.09)	<0.001	0.04 (0.02, 0.06)
									0.30 [b]	0.34 [b]
**Sugar sweetened beverages, servings/d**										
SNAP participants	2.24 (1.65, 2.84)	2.16 (1.58, 2.73)	2.06 (1.72, 2.41)	1.94 (1.55, 2.33)	2.39 (1.91, 2.86)	2.06 (1.88, 2.23)	1.68 (1.43, 1.93)	1.76 (1.43, 2.09)	0.04	-0.43 (-1.09, 0.24)
Income-eligible nonparticipants	2.17 (1.71, 2.63)	1.99 (1.71, 2.27)	1.97 (1.76, 2.17)	1.86 (1.6, 2.12)	1.72 (1.36, 2.09)	1.54 (1.34, 1.73)	1.52 (1.36, 1.69)	1.13 (0.93, 1.32)	<0.001	-1.06 (-1.54, -0.57)
Higher-Income Individuals	1.56 (1.41, 1.70)	1.48 (1.3, 1.66)	1.48 (1.32, 1.65)	1.29 (1.17, 1.42)	1.29 (1.14, 1.44)	1.12 (1.02, 1.22)	1.16 (0.98, 1.34)	0.88 (0.8, 0.97)	<0.001	-0.68 (-0.84, -0.52)

© 2018 Zhang FF et al. *JAMA Network Open.*

									0.11 [b]	0.18 [b]
**Added sugars, tsp equivalents/d, servings/d**										
SNAP participants	23.5 (20.5, 26.5)	25.0 (19.8, 30.2)	20.8 (18.7, 23.0)	19.0 (17.0, 21.0)	22.3 (19.5, 25.1)	20.8 (19.8, 21.8)	18.6 (17.1, 20.2)	19.6 (17.8, 21.3)	0.002	-3.7 (-7.1, -0.4)
Income-eligible nonparticipants	24.2 (21.5, 26.9)	21.1 (19.3, 22.9)	20.7 (19.1, 22.3)	19.7 (17.8, 21.6)	20.3 (17.8, 22.7)	17.8 (16.5, 19.0)	17.6 (16.2, 19.0)	16.1 (14.8, 17.5)	<0.001	-8.0 (-10.9, -5.2)
Higher-Income Individuals	19.6 (18.4, 20.8)	18.7 (17.5, 19.8)	16.9 (16.2, 17.6)	16.1 (15.4, 16.8)	16.4 (15.5, 17.4)	15.5 (15.0, 16.1)	15.7 (14.9, 16.6)	14.1 (13.7, 14.5)	<0.001	-5.6 (-6.8, -4.3)
									0.12 [b]	0.08 [b]
**Total fat, % of energy**										
SNAP participants	32.2 (30.6, 33.7)	32.8 (30.9, 34.7)	33.7 (32.6, 34.9)	34.0 (33.0, 35.0)	33.5 (32.3, 34.6)	32.5 (31.9, 33.0)	33.3 (32.6, 33.9)	33.6 (32.9, 34.2)	0.31	1.5 (-0.2, 3.2)
Income-eligible nonparticipants	32.3 (31.2, 33.4)	33.4 (32.5, 34.4)	34.2 (33.5, 34.9)	32.9 (32.4, 33.5)	32.6 (31.5, 33.8)	32.2 (31.5, 32.9)	33.2 (32.3, 34.0)	34.1 (33.7, 34.6)	0.24	1.9 (0.7, 3.1)
Higher-Income Individuals	33.4 (32.5, 34.2)	33.6 (33.2, 34.1)	34.3 (33.6, 35.0)	34.1 (33.6, 34.5)	34.3 (33.9, 34.6)	33.6 (33.1, 34.1)	33.9 (33.3, 34.6)	35.1 (34.7, 35.5)	0.006	1.7 (0.8, 2.6)
									0.65 [b]	0.91 [b]
**Saturated fat, % of energy**										
SNAP participants	11.2 (10.5, 11.8)	10.8 (10.1, 11.6)	11.5 (10.9, 12.0)	11.9 (11.3, 12.5)	11.1 (10.7, 11.5)	11.0 (10.8, 11.2)	11.3 (11.0, 11.6)	11.2 (10.8, 11.5)	0.94	0.1 (-0.7, 0.8)
Income-eligible nonparticipants	10.5 (10.1, 11.0)	10.6 (10.3, 11.0)	11.4 (10.9, 11.8)	11.0 (10.8, 11.2)	10.8 (10.2, 11.3)	10.6 (10.3, 11.0)	10.9 (10.6, 11.3)	11.4 (11.1, 11.6)	0.04	0.8 (0.3, 1.4)
Higher-Income Individuals	11.2 (10.9, 11.5)	10.6 (10.4, 10.8)	11.3 (11.0, 11.5)	11.3 (11.2, 11.5)	11.3 (11.1, 11.5)	10.9 (10.7, 11.07)	10.8 (10.6, 11.1)	11.2 (11.1, 11.4)	0.72	0.1 (-0.3, 0.4)
									0.24 [b]	0.09 [b]
**Monounsaturated fat, % of energy**										
SNAP participants	18.7 (8.5, 28.9)	16.1 (12.4, 19.8)	14.0 (13.2, 14.9)	14.8 (13.4, 16.1)	15.6 (14.8, 16.5)	13.1 (12.7, 13.5)	13.4 (12.4, 14.4)	13.5 (12.9, 14.1)	0.08	-5.2 (-15.0, 4.5)
Income-eligible nonparticipants	15.47 (14.4, 16.6)	15.5 (14.7, 16.2)	15.0 (14.2, 15.9)	13.8 (12.6, 14.9)	14.7 (13.5, 16.0)	13.7 (12.9, 14.6)	12.8 (12.1, 13.4)	13.5 (12.8, 14.2)	<0.001	-2.1 (-3.4, -0.8)
Higher-Income Individuals	14.2 (13.5, 15.0)	13.4 (13.0, 13.8)	13.6 (13.1, 14.0)	13.6 (13.2, 14.0)	13.8 (13.4, 14.2)	13.0 (12.7, 13.4)	13.0 (12.4, 13.5)	13.5 (13.2, 13.8)	0.01	-0.8 (-1.6, 0.1)
									0.001 [b]	0.16 [b]
**Polyunsaturated fat, % of energy**										
SNAP participants	6.0 (5.7, 6.4)	6.4 (6.0, 6.9)	6.8 (6.4, 7.1)	6.7 (6.2, 7.2)	6.9 (6.6, 7.2)	6.8 (6.5, 7.1)	7.6 (7.4, 7.8)	7.7 (7.4, 7.9)	<0.001	1.6 (1.2, 2.1)
Income-eligible nonparticipants	7.0 (6.7, 7.3)	6.9 (6.6, 7.2)	7.1 (6.7, 7.6)	6.9 (6.7, 7.2)	7.0 (6.7, 7.2)	7.1 (6.9, 7.3)	7.9 (7.5, 8.2)	7.7 (7.5, 8.0)	<0.001	0.7 (0.4, 1.1)
Higher-Income Individuals	7.1 (6.9, 7.2)	6.9 (6.8, 7.0)	7.3 (7.2, 7.5)	7.3 (7.1, 7.5)	7.4 (7.3, 7.5)	7.6 (7.4, 7.7)	8.2 (8.0, 8.4)	8.3 (8.1, 8.4)	<0.001	1.2 (1.0, 1.5)
									0.008 [b]	0.02 [b]
**Seafood omega-3 fat, mg/d**										
SNAP participants	141 (72, 209)	95 (61, 129)	95 (72, 117)	116 (67, 165)	89 (66, 112)	94 (76, 112)	94 (72, 116)	88 (51, 125)	0.16	-54 (-129, 21)
Income-eligible nonparticipants	130 (80, 179)	94 (76, 111)	98 (77, 119)	89 (69, 109)	127 (90, 164)	115 (91, 139)	113 (81, 144)	69 (55, 83)	0.24	-59 (-105, -12)
Higher-Income Individuals	124 (110, 137)	130 (114, 147)	139 (116, 162)	163 (134, 191)	156 (140, 173)	125 (107, 142)	113 (94, 132)	116 (102, 130)	0.08	-7 (-27, 12)
									0.87 [b]	0.08 [b]
**Plant omega-3 fat, mg/d**										
SNAP participants	118 (106, 131)	129 (119, 139)	128 (124, 133)	127 (118, 135)	131 (125, 137)	134 (127, 141)	156 (152, 159)	147 (142, 152)	<0.001	28 (15, 42)
Income-eligible nonparticipants	127 (119, 135)	133 (124, 142)	144 (134, 154)	135 (130, 139)	135 (128, 142)	140 (135, 145)	164 (155, 172)	149 (144, 155)	<0.001	22 (13, 32)

© 2018 Zhang FF et al. *JAMA Network Open.*

Higher-Income	140	139	145	144	144	152	168	165		24
Individuals	(134, 146)	(136, 141)	(141, 149)	(139, 149)	(140, 147)	(149, 155)	(164, 173)	(160, 169)	<0.001	(17, 32)
									0.62 [b]	0.79 [b]
**Polyunsaturated: saturated fat ratio**										
SNAP	0.61	0.69	0.64	0.62	0.67	0.67	0.73	0.75		0.13
participants	(0.56, 0.66)	(0.64, 0.73)	(0.61, 0.68)	(0.55, 0.68)	(0.64, 0.7)	(0.63, 0.71)	(0.71, 0.75)	(0.71, 0.79)	<0.001	(0.07, 0.2)
Income-eligible	0.73	0.74	0.69	0.68	0.73	0.73	0.77	0.73	0.15	0.01
nonparticipants	(0.69, 0.78)	(0.69, 0.79)	(0.64, 0.74)	(0.65, 0.72)	(0.65, 0.81)	(0.69, 0.76)	(0.74, 0.8)	(0.7, 0.76)		(-0.05, 0.06)
Higher-Income	0.71	0.73	0.70	0.70	0.71	0.76	0.82	0.80		0.08
Individuals	(0.69, 0.74)	(0.71, 0.75)	(0.69, 0.72)	(0.69, 0.72)	(0.70, 0.73)	(0.74, 0.78)	(0.79, 0.84)	(0.78, 0.81)	<0.001	(0.05, 0.11)
									0.006 [b]	0.01 [b]
**Protein, % of energy**										
SNAP	15.5	14.5	14.9	15.7	15.3	15.3	15.3	15.5		-0.03
participants	(14.7, 16.3)	(13.7, 15.3)	(14.3, 15.5)	(14.9, 16.5)	(14.9, 15.8)	(15.0, 15.6)	(14.8, 15.9)	(14.9, 16.1)	0.21	(-1.0, 0.9)
Income-eligible	14.8	15.0	14.7	15.7	15.5	15.9	15.6	15.7		0.9
nonparticipants	(14.3, 15.3)	(14.4, 15.5)	(14.2, 15.3)	(14.8, 16.6)	(15.0, 15.9)	(15.4, 16.3)	(15.2, 16.0)	(15.1, 16.4)	<0.001	(0.1, 1.7)
Higher-Income	15.5	15.3	15.8	16.1	16.0	16.1	15.7	16.4		0.9
Individuals	(15.2, 15.8)	(15.0, 15.6)	(15.6, 16.0)	(15.9, 16.4)	(15.8, 16.2)	(15.8, 16.4)	(15.4, 16.0)	(16.1, 16.7)	<0.001	(0.5, 1.3)
									0.47 [b]	0.14 [b]
**Carbohydrate, % of energy**										
SNAP	50.4	52.4	50.1	48.3	50.2	50.91	49.8	49.5		-0.9
participants	(48.9, 51.9)	(50.1, 54.7)	(49.1, 51.1)	(45.9, 50.6)	(48.6, 51.8)	(50.1, 51.7)	(48.7, 51.0)	(48.5, 50.5)	0.11	(-2.6, 0.8)
come-eligible	52.5	51.0	50.1	50.3	50.2	49.8	50.4	48.5		-4.0
onparticipants	(51.4, 53.7)	(49.8, 52.3)	(49.3, 51.0)	(49.2, 51.4)	(49.1, 51.2)	(49.1, 50.5)	(49.4, 51.4)	(47.6, 49.4)	<0.001	(-5.5, -2.6)
Higher-Income	49.9	50.2	48.5	48.3	48.4	48.5	48.2	46.5		-3.4
Individuals	(49.1, 50.8)	(49.4, 51.0)	(47.7, 49.2)	(47.7, 49.0)	(47.9, 48.9)	(48.0, 49.0)	(47.4, 49.0)	(46.1, 47.0)	<0.001	(-4.4, -2.5)
									0.17 [b]	0.02 [b]
**Cholesterol, mg/d**										
SNAP	332	259	278	288	303	273	276	259		-73
participants	(292, 371)	(226, 291)	(263, 292)	(258, 318)	(290, 315)	(262, 284)	(259, 292)	(247, 272)	0.005	(-113, -32)
Income-eligible	269	274	277	278	278	281	263	263		-9.46
nonparticipants	(247, 292)	(254, 294)	(254, 300)	(258, 298)	(269, 287)	(254, 309)	(246, 280)	(239, 288)	0.40	(-42.38, 23.46)
Higher-Income	271	259	277	277	281	264	270	276		5.79
Individuals	(259, 283)	(250, 268)	(267, 287)	(269, 285)	(271, 291)	(257, 271)	(258, 282)	(268, 285)	0.37	(-8.45, 20.03)
									0.02 [b]	0.014 [b]
**Fiber, g/d**										
SNAP	13.1	14.2	13.3	13.7	14.0	14.8	16.1	13.7		0.5
participants	(11.4, 14.9)	(13.0, 15.5)	(12.5, 14.0)	(12.8, 14.5)	(13.1, 14.9)	(14.3, 15.4)	(14.9, 17.3)	(12.8, 14.6)	0.03	(-1.5, 2.5)
Income-eligible	14.8	14.6	14.3	15.4	14.9	16.0	17.27	14.99		0.1
nonparticipants	(13.8, 15.7)	(13.6, 15.7)	(13.7, 14.8)	(14.6, 16.2)	(13.5, 16.4)	(15.4, 16.6)	(16.4, 18.2)	(14.0, 16.0)	0.004	(-1.3, 1.5)
Higher-Income	15.6	15.7	15.4	16.0	16.5	17.5	17.7	16.6		1.1
Individuals	(14.8, 16.3)	(15.3, 16.2)	(14.9, 15.9)	(15.6, 16.5)	(15.8, 17.1)	(17.0, 17.9)	(17.1, 18.4)	(16.2, 17.0)	<0.001	(0.2, 1.9)
									0.63 [b]	0.55 [b]
**Sodium, mg/d**										
SNAP	3434	3371	3332	3359	3436	3396	3382	3166		-273
participants	(3135, 3732)	(3128, 3613)	(3236, 3428)	(3178, 3540)	(3342, 3529)	(3336, 3457)	(3259, 3504)	(3084, 3249)	0.07	(-571, 24)
Income-eligible	3237	3356	3415	3410	3356	3438	3502	3187		-53
nonparticipants	(3096, 3378)	(3232, 3480)	(3298, 3532)	(3264, 3556)	(3238, 3474)	(3318, 3558)	(3410, 3594)	(3083, 3291)	0.63	(-223, 117)
Higher-Income	3432	3338	3458	3566	3516	3456	3383	3296		-132
Individuals	(3363, 3501)	(3301, 3376)	(3414, 3503)	(3502, 3629)	(3466, 3566)	(3414, 3498)	(3337, 3428)	(3247, 3345)	0.05	(-216, -49)
									0.25 [b]	0.56 [b]
**Potassium, mg/d**										
SNAP	2538	2393	2496	2658	2473	2548	2541	2292		-253
participants	(2361, 2716)	(2240, 2547)	(2414, 2579)	(2550, 2766)	(2388, 2557)	(2488, 2608)	(2470, 2613)	(2225, 2358)	0.02	(-440, -66)

© 2018 Zhang FF et al. *JAMA Network Open.*

Income-eligible	2567	2429	2502	2623	2521	2639	2630	2364		-208
nonparticipants	(2479, 2655)	(2349, 2509)	(2421, 2584)	(2538, 2708)	(2410, 2632)	(2561, 2716)	(2556, 2704)	(2270, 2458)	0.75	(-338, -78)
Higher-Income	2742	2678	2644	2691	2656	2768	2731	2551		-189
Individuals	(2670, 2814)	(2618, 2738)	(2607, 2681)	(2634, 2748)	(2601, 2711)	(2730, 2806)	(2667, 2795)	(2505, 2597)	0.08	(-274, -104)
									0.24 [b]	0.81 [b]
**Magnesium, mg/d**										
SNAP	243	232	240	278	258	275	280	253		8
participants	(227, 260)	(217, 247)	(229, 251)	(266, 289)	(248, 269)	(267, 283)	(269, 291)	(241, 266)	0.0005	(-12, 28)
Income-eligible	258	251	250	280	283	292	284	271		12
nonparticipants	(246, 271)	(239, 263)	(241, 259)	(271, 290)	(261, 305)	(285, 300)	(275, 293)	(259, 283)	<0.001	(-5, 30)
Higher-Income	273.57	271	270	297	294	307	305	291		18
Individuals	(264.29, 282.85)	(263, 278)	(264, 275)	(291, 303)	(286, 302)	(302, 312)	(297, 314)	(286, 297)	<0.001	(8, 29)
									0.71 [b]	0.55 [b]
**Calcium, mg/d**										
SNAP	737	718	798	895	816	954	990	871		126
participants	(636, 838)	(678, 758)	(747, 849)	(818, 972)	(775, 858)	(924, 983)	(940, 1039)	(838, 904)	<0.001	(25, 228)
Income-eligible	733	758	790	888	905	949	981	894		161
nonparticipants	(695, 772)	(693, 823)	(749, 831)	(843, 933)	(837, 973)	(914, 984)	(906, 1057)	(852, 937)	<0.001	(104, 218)
Higher-Income	809	830	841	920	927	1003	940	917		109
Individuals	(775, 844)	(806, 853)	(818, 864)	(898, 941)	(901, 954)	(984, 1023)	(916, 964)	(890, 935)	<0.001	(71, 148)
									0.03 [b]	0.33 [b]

Abbreviations: AHA, American Heart Association; SNAP: Supplemental Nutrition Assistance Program

[a] Survey-weighted mean scores were adjusted for age, sex, and race/ethnicity

[b] Survey-weighted P for interaction that assessed whether the trend in mean take or change in mean intake differed by SNAP participation status

© 2018 Zhang FF et al. *JAMA Network Open.*

eTable 7. Crude and Adjusted Trends in the American Heart Association (AHA) 2020 Diet Score by SNAP Participation Status, 2003-2014[a]

	Survey-Weighted Mean Score (95% CI) [a]						P for Trend	Mean Change From 2003-2004 to 2013-2014 (95% CI)
	2003-2004 (n=4066)	2005-2006 (n=4030)	2007-2008 (n=4654)	2009-2010 (n=4996)	2011-2012 (n=4288)	2013-2014 (n=4437)		
**Total AHA diet score (unadjusted)**								
SNAP participants	31.5 (29.2, 33.8)	30.8 (28.6, 32.9)	31.1 (29.5, 32.8)	32.8 (31.2, 34.5)	33.8 (32.7, 34.8)	32.1 (30.6, 33.6)	0.11	0.57 (-2.18, 0.33)
Lower-income SNAP participants	30.6 (28.4,32.8)	30.4(27.6,33.3)	30.9(28.7,33.2)	32.5(31.3,33.7)	32.9(31.1,34.8)	31.7(30,33.4)	0.14	1.10 (-1.68, 3.88)
High-income SNAP participants	33.8 (30.6, 37.0)	31.3(28.9,33.7)	30.9(28.2,33.6)	33.8(29.7,37.9)	35.1(33.5,36.7)	32.2(29.8,34.6)	0.54	-1.56 (-5.51, 2.39)
Income-eligible nonparticipants	34.2 (32.6, 35.9)	34.7 (33.1, 36.3)	34.9 (32.7, 37.1)	36.3 (34.8, 37.9)	36.5 (35.2, 37.7)	36.8 (35.3, 38.3)	0.004	2.56 (0.36, 4.76)
Higher-income individuals	35.8 (34.6, 36.9)	36.4 (35.3, 37.4)	36.7 (35.5, 37.8)	38.7 (37.9, 39.4)	40.0 (38.5, 41.5)	39.6 (38.7, 40.5)	<0.001	3.84 (2.39, 5.29)
						P-interaction	0.02 [b]	0.04 [c]
**Total AHA diet score (adjusted for change in age, sex, and race/ethnicity)**								
SNAP participants	32.5 (30.4, 34.6)	31.9 (29.8, 34.1)	31.9 (30.4, 33.3)	33.3 (31.8, 34.8)	34.4 (33.4, 35.5)	32.5 (30.9, 34.1)	0.21	0.01 (-2.17, 2.20)
Lower-income SNAP participants	31.4(29.5,33.4)	31.4(28.6,34.3)	31.4(29.5,33.3)	32.5(31.4,33.5)	33.2(31.3,35.2)	31.8(30.1,33.5)	0.36	0.32 (-1.95, 2.58)
Higher-income SNAP participants	35.0(31.8,38.1)	32.3(29.9,34.7)	32.1(29.0,35.3)	35.0(30.9,39.1)	36.0(34.4,37.6)	33.0(30.5,35.5)	0.33	-1.00 (-4.60, 2.60)
Income-eligible nonparticipants	34.4 (32.8, 35.9)	34.4 (32.9, 36.0)	34.6 (32.2, 37.1)	36.3 (34.7, 38.0)	36.4 (35.0, 37.7)	36.7 (35.2, 38.3)	0.008	2.16 (0.01, 4.30)
Higher-income individuals	35.9 (34.8, 37.1)	36.5 (35.4, 37.5)	36.7 (35.5, 37.8)	38.5 (37.8, 39.2)	39.7 (38.1, 41.2)	39.2 (38.3, 40.2)	<0.001	3.33 (1.86, 4.80)
						P-interaction	0.01 [b]	0.02 [c]
**Total AHA diet score (adjusted for change in age, sex, race/ethnicity, and education)**								
SNAP participants	34.2 (32.2,36.1)	33.4(31.2,35.5)	33.7(32.3,35.1)	35(33.6,36.4)	35.8(34.7,36.8)	33.9(32.4,35.4)	0.35	-0.25 (-2.36, 1.89)
Lower-income SNAP participants	31.9(30.0,33.8)	31.8(29.0,34.7)	32.0(30.2,33.8)	32.9(31.9,33.9)	33.5(31.7,35.4)	32.0(30.4,33.7)	0.48	0.13 (-2.13,2.40)
Higher-income SNAP participants	36.7(33.7,39.8)	33.6(35.7,37.9)	33.8(30.9,36.7)	36.9(33.0,40.8)	37.1(35.5,38.8)	34.3(31.9,36.7)	0.55	-1.56 (-5.04,1.93)
Income-eligible nonparticipants	35.8 (34.3,37.3)	35.6(34.1,37.1)	35.9(33.8,38)	37.1(35.7,38.6)	37.3(36.2,38.4)	37.4(35.9,39)	0.04	1.51 (-0.54, 3.57)
Higher-income individuals	35.9 (34.9,36.8)	36.3(35.3,37.3)	36.5(35.5,37.4)	38.1(37.4,38.7)	38.8(37.3,40.2)	38.3(37.4,39.2)	<0.001	2.48 (1.23, 3.72)
						P-interaction	0.06 [b]	0.048 [c]
**Total AHA diet score (adjusted for change in age, sex, race/ethnicity, and income)**								
SNAP participants	34.8 (32.8, 36.9)	33.9 (31.7,36.1)	34.2(32.6,35.9)	35.7 (34.3,37.0)	36.6(35.4,37.7)	34.5(33,36)	0.27	-0.16 (-2.33, 2.01)
Income-eligible nonparticipants	37.0 (35.4, 38.6)	37.1 (35.5,38.7)	37.3(34.9,39.6)	39.0 (37.3,40.7)	39.1(37.6,40.6)	39.3(37.6,40.9)	<0.01	2.18 (0.04, 4.32)
Higher-income individuals	35.1 (34.0, 36.2)	35.6 (34.5,36.7)	35.8(34.5,37.1)	37.6(36.9,38.3)	38.9(37.3,40.5)	38.3(37.4,39.2)	<0.001	3.17 (1.78, 4.56)
						P-interaction	0.04 [b]	0.01 [c]

Abbreviations: AHA, American Heart Association; SNAP: Supplemental Nutrition Assistance Program

[a]. The total AHA diet score is the sum of the scores for 8 primary dietary components (fruits/vegetables, whole grains, fish/shellfish, sugar-sweetened beverages, and sodium, nuts/seeds/legumes, processed meat, and saturated fat).

[b]. P for interaction assessing potential heterogeneous trends in total AHA diet score by SNAP participation status (SNAP participants, income-eligible nonparticipants, and higher-income individuals)

[c]. P for interaction assessing potential heterogeneous change in total AHA diet score from 2003-2004 to 2013-2014 by SNAP participation status (SNAP participants, income-eligible nonparticipants, and higher-income individuals)

© 2018 Zhang FF et al. *JAMA Network Open.*

eTable 8. Mean Change in Dietary Components of the American Heart Association (AHA) 2020 Strategic Impact Goals among SNAP Participants from 1999-2002 to 2011-2014, by Age

Dietary Intake	Survey-Weighted Mean Intake or Change (95% CI)												P for Interaction
	**Age 20-34**		Mean Change (95% CI)	**Age 35-49**		Mean Change (95% CI)	**Age 50-64**		Mean Change (95% CI)	**Age ≥65**		Mean Change (95% CI)	
	1999-2002 (n=328)	2011-2014 (n=768)		1999-2002 (n=204)	2011-2014 (n=578)		1999-2002 (n=161)	2011-2014 (n=575)		1999-2002 (n=147)	2011-2014 (n=322)		
Fruits and vegetables, servings/d	1.08 (0.85, 1.31)	1.19 (1.06, 1.33)	0.11 (- 0.15, 0.38)	1.31 (0.97, 1.65)	1.27 (1.11, 1.43)	-0.04 (-0.41,0.33)	1.52 (1.28,1.76)	1.70 (1.4,2.0)	0.18 (-0.2,0.55)	2.00 (1.53, 2.47)	2.02 (1.73, 2.31)	0.02 (-0.52, 0.56)	0.91
Whole grains, servings/d	0.32 (0.21, 0.44)	0.66 (0.56, 0.77)	0.34*** (0.19, 0.49)	0.34 (0.21, 0.47)	0.60 (0.50, 0.70)	0.26** (0.10,0.42)	0.68 (0.43,0.92)	0.80 (0.69,0.91)	0.12 (-0.14,0.38)	0.59 (0.32, 0.87)	1.06 (0.89, 1.24)	0.47** (0.15, 0.79)	0.79
Fish and shellfish, servings/d	0.10 (0.04, 0.16)	0.14 (0.09, 0.18)	0.03 (-0.04, 0.11)	0.14 (0.04, 0.24)	0.24 (0.17, 0.31)	0.10 (-0.01,0.21)	0.10 (0.05,0.16)	0.17 (0.12,0.21)	0.06 (-0.01,0.13)	0.14 (0.05, 0.22)	0.19 (0.10, 0.28)	0.06 (-0.07, 0.18)	0.67
Sugar-sweetened beverages, servings/d	3.04 (2.40, 3.67)	2.29 (1.99, 2.59)	-0.75* (-1.43, -0.06)	2.38 (1.78, 2.98)	1.97 (1.70, 2.24)	-0.41 (-1.05,0.24)	1.86 (1.52,2.19)	1.41 (1.11,1.71)	-0.44* (-0.89,0)	0.94 (0.67, 1.21)	0.69 (0.48, 0.90)	-0.25 (-0.58, 0.08)	0.27
Sodium, mg/d	3138 (2945, 3332)	3125 (3045, 3205)	-13 (-219, 192)	3386 (2989, 3782)	3247 (3118, 3377)	-138 (-547,271)	3504 (3200,3809)	3293 (3186,3401)	-211 (-528,105)	3806 (3498, 4114)	3529 (3365, 3692)	-277 (-618, 64)	0.10
Nuts, seeds, and legumes, servings/d	0.23 (0.15, 0.31)	0.40 (0.32, 0.49)	0.17** (0.05, 0.29)	0.27 (0.17, 0.37)	0.38 (0.27, 0.48)	0.10 (-0.03,0.24)	0.34 (0.17,0.51)	0.46 (0.31, 0.61)	0.12 (-0.1, 0.35)	0.88 (-0.4, 2.17)	0.52 (0.4, 0.65)	-0.36 (-1.63, 0.9)	0.39
Processed meat, servings/d	0.26 (0.19, 0.32)	0.29 (0.27, 0.32)	0.03 (-0.03, 0.10)	0.34 (0.20, 0.47)	0.30 (0.25, 0.36)	-0.03 (-0.17, 0.11)	0.16 (0.10, 0.22)	0.33 (0.23, 0.42)	0.16** (0.06, 0.27)	0.21 (0.12, 0.31)	0.27 (0.2, 0.33)	0.06 (-0.06, 0.17)	0.19
Saturated fat, % of energy	11.0 (10.5, 11.5)	10.9 (10.6, 11.3)	-0.04 (-0.7, 0.6)	10.7 (9.9, 11.6)	10.8 (10.4, 11.1)	0.02 (-0.9, 0.9)	10.3 (9.5, 11.1)	10.8 (10.3, 11.2)	0.47 (-0.5, 1.4)	10.0 (9.0, 10.9)	10.9 (10.3, 11.4)	0.9 (-0.2, 2.0)	0.08

Abbreviations: AHA, American Heart Association; SNAP: Supplemental Nutrition Assistance Program

a. The mean change from 1999-2002 to 2011-2014 was statistically significant (*p<0.05; ** p<0.01; *** p<0.001)

© 2018 Zhang FF et al. *JAMA Network Open.*

eTable 9. Mean Change in Dietary Components of the American Heart Association (AHA) 2020 Strategic Impact Goals among SNAP Participants from 1999-2002 to 2011-2014, by Gender

Dietary Intake	Survey-Weighted Mean Intake or Change (95% CI)						P for Interaction
	**Male**		Mean Change (95% CI)	**Female**		Mean Change (95% CI)	
	1999-2002 (n=313)	2011-2014 (n=1011)		1999-2002 (n=527)	2011-2014 (n=1232)		
Fruits and vegetables, servings/d	1.32 (1.09, 1.55)	1.21 (1.09, 1.33)	-0.11 (-0.36,0.14)	1.32 (1.15, 1.50)	1.61 (1.41, 1.80)	0.28* (0.03, 0.54)	0.01
Whole grains, servings/d	0.44 (0.31, 0.57)	0.66 (0.57, 0.74)	0.22** (0.06,0.37)	0.41 (0.29, 0.53)	0.78 (0.69, 0.86)	0.37*** (0.22, 0.51)	0.14
Fish and shellfish, servings/d	0.13 (0.06, 0.20)	0.19 (0.14, 0.23)	0.06 (-0.03,0.14)	0.11 (0.06, 0.16)	0.17 (0.13, 0.21)	0.06 (-0.01, 0.13)	0.92
Sugar-sweetened beverages, servings/d	2.33 (1.98, 2.69)	1.96 (1.74, 2.19)	-0.37 (-0.78,0.04)	2.47 (2.14, 2.80)	1.71 (1.48, 1.93)	-0.76*** (-1.15, -0.37)	0.06
Sodium, mg/d	3341 (3176, 3506)	3139 (3057, 3222)	-202* (-383, -21)	3346 (3146, 3546)	3325 (3251, 3398)	-22 (-230, 187)	
Nuts, seeds, and legumes, servings/d	0.28 (0.20, 0.35)	0.42 (0.32, 0.51)	0.14* (0.02, 0.26)	0.37 (0.12, 0.62)	0.43 (0.33, 0.52)	0.06 (-0.21, 0.32)	0.20
Processed meat, servings/d	0.32 (0.24, 0.41)	0.38 (0.31, 0.44)	0.05 (-0.05, 0.16)	0.22 (0.17, 0.26)	0.24 (0.21, 0.27)	0.02 (-0.03, 0.07)	0.55
Saturated fat, % of energy	10.7 (10.3, 11.2)	10.7 (10.5, 11.0)	-0.01 (-0.5, 0.5)	10.6 (10.1, 11.1)	10.9 (10.6, 11.2)	0.3 (-0.3, 0.9)	0.56

Abbreviations: AHA, American Heart Association; SNAP: Supplemental Nutrition Assistance Program

a. The mean change from 1999-2002 to 2011-2014 was statistically significant (*p<0.05; ** p<0.01; *** p<0.001)

© 2018 Zhang FF et al. *JAMA Network Open.*

eTable 10. Mean Change in Dietary Components of the American Heart Association (AHA) 2020 Strategic Impact Goals among SNAP Participants from 1999-2002 to 2011-2014, by Race/Ethnicity

Dietary Intake	Survey-Weighted Mean Intake or Change (95% CI)									P for Interaction
	**Non-Hispanic White**		Mean Change (95% CI)	**Non-Hispanic Black**		Mean Change (95% CI)	**Hispanic**		Mean Change (95% CI)	
	1999-2002 (n=233)	2011-2014 (n=773)		1999-2002 (n=300)	2011-2014 (n=784)		1999-2002 (n=276)	2011-2014 (n=512)		
Fruits and vegetables, servings/d	1.39 (1.06, 1.71)	1.40 (1.18, 1.62)	0.02 (-0.37, 0.40)	1.18 (1.00, 1.36)	1.23 (1.08, 1.38)	0.05 (-0.18, 0.28)	1.34 (1.14, 1.54)	1.65 (1.50, 1.80)	0.31* (0.07, 0.56)	0.25
Whole grains, servings/d	0.49 (0.31, 0.68)	0.72 (0.61, 0.84)	0.23* (0.02, 0.44)	0.35 (0.23, 0.47)	0.70 (0.59, 0.81)	0.35*** (0.19, 0.51)	0.37 (0.20, 0.54)	0.66 (0.51, 0.81)	0.29* (0.06, 0.51)	0.64
Fish and shellfish, servings/d	0.06 (0.02, 0.11)	0.12 (0.07, 0.16)	0.06 (-0.01, 0.12)	0.29 (0.19, 0.38)	0.26 (0.18, 0.34)	-0.03 (-0.15, 0.09)	0.06 (0.02, 0.10)	0.18 (0.13, 0.24)	0.12*** (0.06, 0.19)	0.09
Sugar-sweetened beverages, servings/d	2.39 (1.75, 3.03)	2.01 (1.70, 2.32)	-0.38 (-1.07, 0.31)	2.53 (2.25, 2.80)	1.77 (1.62, 1.92)	-0.76*** (-1.07, -0.45)	2.11 (1.41, 2.81)	1.45 (1.23, 1.67)	-0.66 (-1.38, 0.06)	0.64
Sodium, mg/d	3433 (3111, 3755)	3237 (3134, 3340)	-196 (-527, 135)	3285 (3146, 3424)	3271 (3167, 3375)	-14 (-183, 156)	3173 (3036, 3311)	3194 (3074, 3315)	21 (-158, 200)	0.59
Nuts, seeds, and legumes, servings/d	0.28 (0.17, 0.38)	0.43 (0.31, 0.55)	0.16* (0, 0.31)	0.48 (-0.16, 1.13)	0.36 (0.29, 0.42)	-0.13 (-0.76, 0.5)	0.35 (0.18, 0.51)	0.40 (0.29, 0.51)	0.05 (-0.14, 0.25)	0.55
Processed meat, servings/d	0.27 (0.18, 0.36)	0.34 (0.27, 0.42)	0.07 (-0.04, 0.19)	0.32 (0.27, 0.37)	0.31 (0.28, 0.33)	-0.01 (-0.06, 0.04)	0.18 (0.13, 0.23)	0.21 (0.18, 0.25)	0.03 (-0.03, 0.09)	0.26
Saturated fat, % of energy	11.2 (10.5, 11.9)	11.4 (11.1, 11.8)	0.2 (-0.5, 1.0)	10.1 (9.6, 10.5)	10.7 (10.3, 11.1)	0.6* (0.1, 1.2)	10.3 (9.4, 11.2)	10.1 (9.6, 10.6)	-0.2 (-1.2, 0.8)	0.29

Abbreviations: AHA, American Heart Association; SNAP: Supplemental Nutrition Assistance Program

a. The mean change from 1999-2002 to 2011-2014 was statistically significant (*p<0.05; ** p<0.01; *** p<0.001)

© 2018 Zhang FF et al. *JAMA Network Open.*

eTable 11. Mean Change in Dietary Components of the American Heart Association (AHA) 2020 Strategic Impact Goals among SNAP Participants from 1999-2002 to 2011-2014, by Education

Dietary Intake	Survey-Weighted Mean Intake or Change (95% CI)									P for Interaction
	**< High School**		Mean Change (95% CI)	**High School Graduate or Equivalent**		Mean Change (95% CI)	**Some College or Above**		Mean Change (95% CI)	
	1999-2002 (n=485)	2011-2014 (n=790)		1999-2002 (n=184)	2011-2014 (n=638)		1999-2002 (n=168)	2011-2014 (n=813)		
Fruits and vegetables, servings/d	1.35 (1.15, 1.55)	1.29 (1.15, 1.42)	-0.06 (-0.31,0.18)	1.28 (1.01, 1.56)	1.28 (1.11, 1.45)	0 (-0.32,0.32)	1.30 (1.05, 1.54)	1.63 (1.39, 1.87)	0.34* (0,0.67)	0.06
Whole grains, servings/d	0.34 (0.22, 0.47)	0.63 (0.5, 0.76)	0.29** (0.11,0.47)	0.38 (0.24, 0.52)	0.72 (0.63, 0.82)	0.34*** (0.18,0.51)	0.60 (0.39, 0.81)	0.79 (0.69, 0.89)	0.19 (-0.04,0.42)	0.55
Fish and shellfish, servings/d	0.14 (0.07, 0.21)	0.19 (0.14, 0.25)	0.05 (-0.03,0.14)	0.06 (0.02, 0.10)	0.15 (0.10, 0.20)	0.09** (0.03,0.15)	0.14 (0.06, 0.22)	0.19 (0.14, 0.23)	0.05 (-0.04,0.14)	0.87
Sugar-sweetened beverages, servings/d	2.19 (1.94, 2.44)	1.84 (1.54, 2.14)	-0.35 (-0.73,0.03)	2.72 (1.80, 3.63)	2.03 (1.82, 2.25)	-0.68 (-1.6,0.24)	2.57 (1.92, 3.22)	1.66 (1.4, 1.92)	-0.91** (-1.59,-0.23)	0.09
Sodium, mg/d	3436 (3248, 3625)	3150 (3055, 3246)	-286** (-493, -79)	3332 (3044, 3620)	3273 (3176, 3370)	-59 (-356, 238)	3186 (2860, 3512)	3283 (3174, 3393)	97 (-240, 434)	0.09
Nuts, seeds, and legumes, servings/d	0.42 (0.11, 0.74)	0.34 (0.21, 0.47)	-0.09 (-0.42,0.25)	0.19 (0.10, 0.29)	0.40 (0.30, 0.49)	0.21** (0.08,0.34)	0.32 (0.19, 0.46)	0.50 (0.39, 0.62)	0.18* (0.01,0.35)	0.12
Processed meat, servings/d	0.22 (0.18, 0.27)	0.26 (0.22, 0.3)	0.03 (-0.02,0.09)	0.30 (0.19, 0.41)	0.31 (0.25, 0.38)	0.01 (-0.11,0.14)	0.27 (0.14, 0.41)	0.33 (0.27, 0.38)	0.05 (-0.09,0.19)	0.92
Saturated fat, % of energy	10.5 (10.1, 10.8)	10.5 (10.2, 10.9)	0.1 (-0.4, 0.6)	11.2 (10.2, 12.3)	10.8 (10.4, 11.2)	-0.4 (-1.5, 0.7)	10.5 (9.7, 11.4)	11.1 (10.8, 11.4)	0.6 (-0.3, 1.4)	0.44

Abbreviations: AHA, American Heart Association; SNAP: Supplemental Nutrition Assistance Program

a. The mean change from 1999-2002 to 2011-2014 was statistically significant (*p<0.05; ** p<0.01; *** p<0.001)

© 2018 Zhang FF et al. *JAMA Network Open.*

eFigure 1a. Percent Change in Intake of AHA Dietary Components and Key Food Groups Among U.S. Adults by SNAP Participation Status, From 1999-2000 to 2013-2014

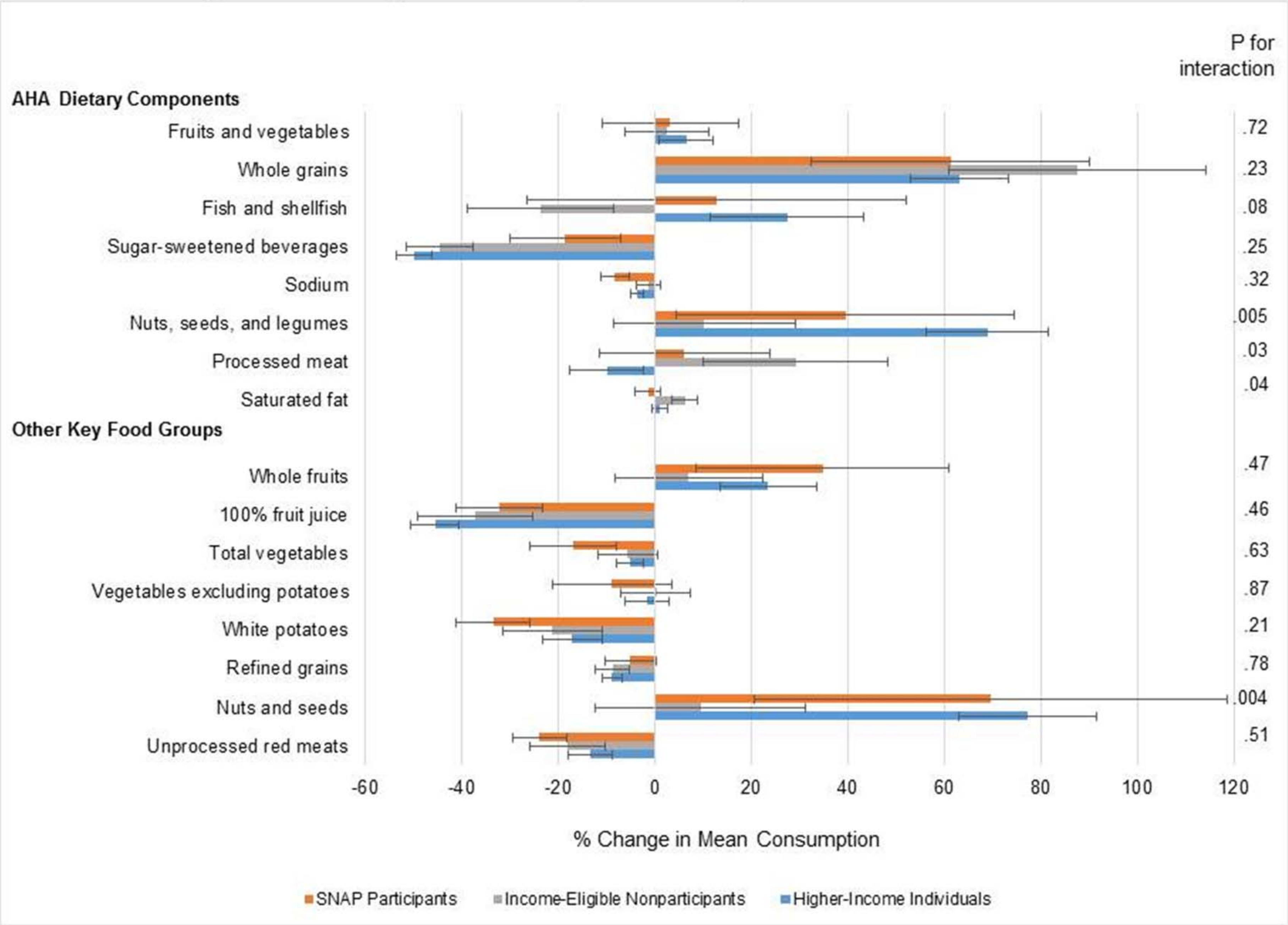

© 2018 Zhang FF et al. *JAMA Network Open.*

eFigure 1b. Percent Change in Dietary Intake of Other Key Nutrients Among U.S. Adults by SNAP Participation Status, From 1999-2000 to 2013-2014

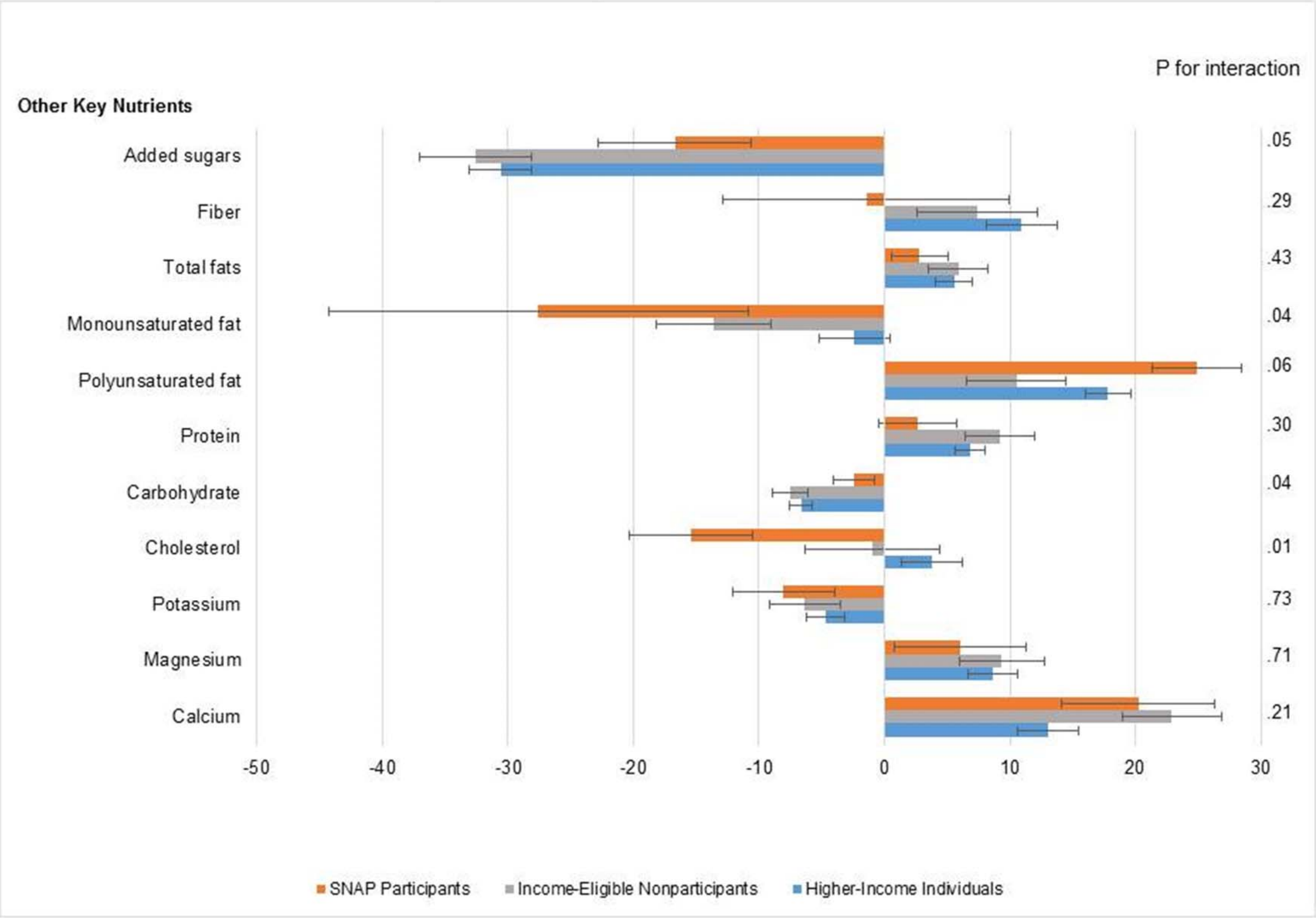

© 2018 Zhang FF et al. *JAMA Network Open.*

eFigure 2a. Percent of US Adults Meeting Dietary Recommendations for AHA 2020 Strategic Goals by SNAP Participation Status, 2013-2014

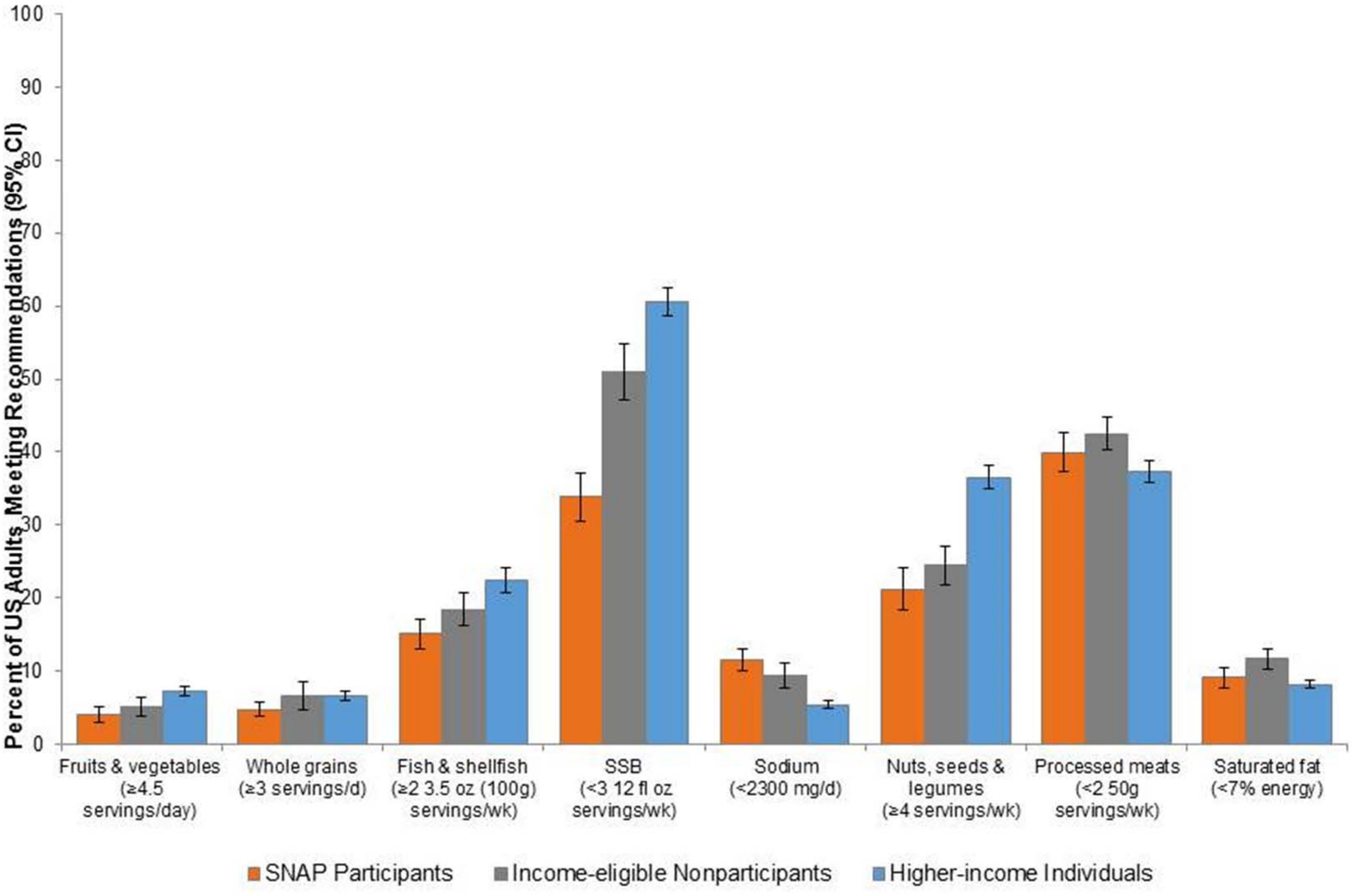

© 2018 Zhang FF et al. *JAMA Network Open.*

eFigure 2b. Percent of US Adults Meeting Dietary Recommendations for Other Key Food Groups and Nutrients by SNAP Participation Status, 2013-2014

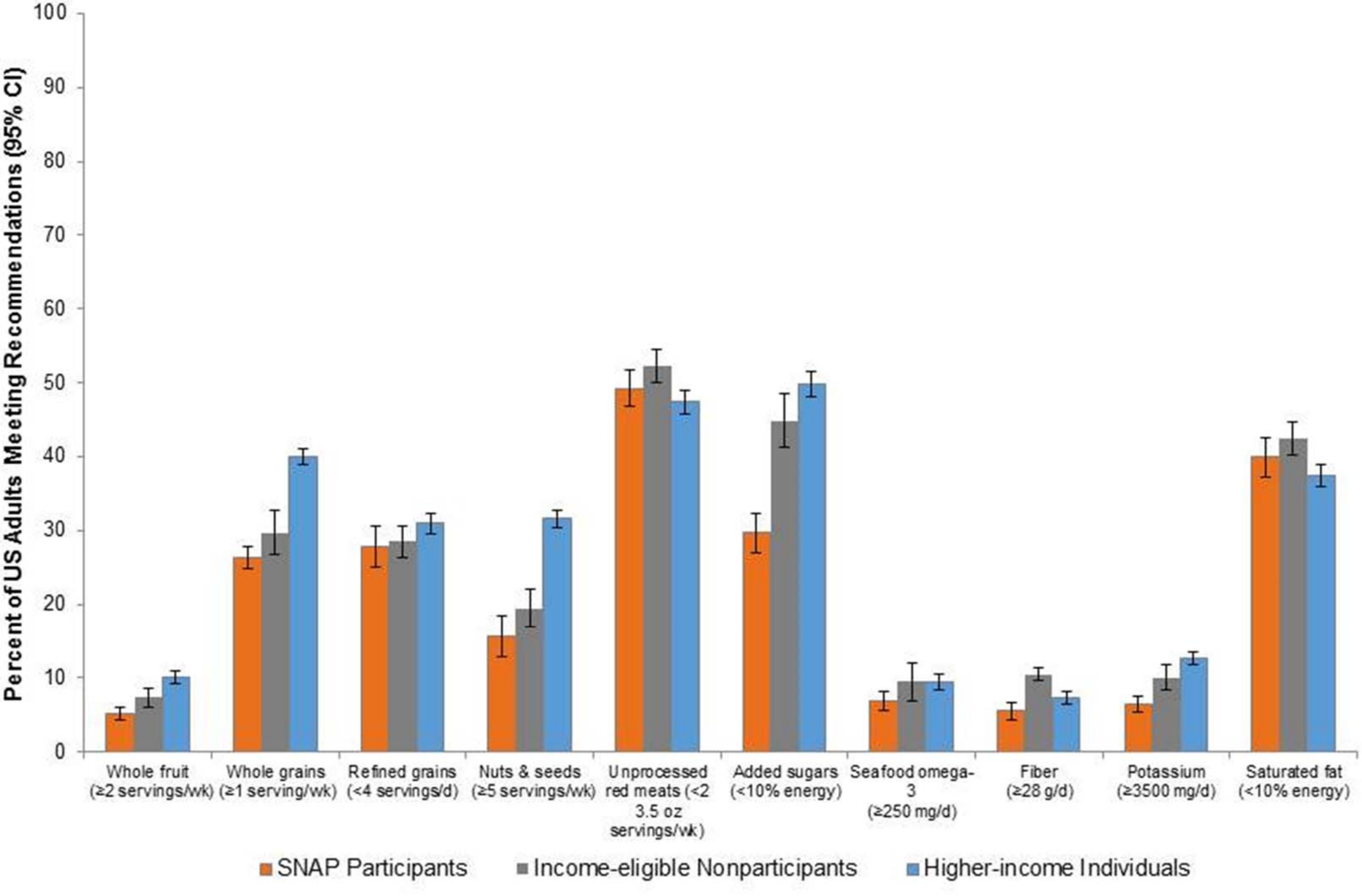

© 2018 Zhang FF et al. *JAMA Network Open.*
